# In Vitro Acquisition of Specific Small Interfering RNAs Inhibits the Expression of Some Target Genes in the Plant Ectoparasite *Xiphinema index*

**DOI:** 10.3390/ijms20133266

**Published:** 2019-07-03

**Authors:** Aurélie Marmonier, Laetitia Perfus-Barbeoch, Corinne Rancurel, Sylvaine Boissinot, Bruno Favery, Gérard Demangeat, Véronique Brault

**Affiliations:** 1 SVQV, INRA, Université de Strasbourg, 68000 Colmar, France; 2 ISA, INRA, Université Côte d’Azur, CNRS, 06900 Sophia-Antipolis, France

**Keywords:** plant nematode, RNA interference, *Xiphinema index*, siRNA

## Abstract

*Xiphinema index* is an important plant parasitic nematode that induces direct damages and specifically transmits the *Grapevine fanleaf virus*, which is particularly harmful for grapevines. Genomic resources of this nematode species are still limited and no functional gene validation technology is available. RNA interference (RNAi) is a powerful technology to study gene function and here we describe the application of RNAi on several genes in *X. index*. Soaking the nematodes for 48 h in a suspension containing specific small interfering RNAs resulted in a partial inhibition of the accumulation of some targeted mRNA. However, low reproducible silencing efficiency was observed which could arise from *X. index* silencing pathway deficiencies. Indeed, essential accustomed proteins for these pathways were not found in the *X. index* proteome predicted from transcriptomic data. The most reproducible silencing effect was obtained when targeting the *piccolo* gene potentially involved in endo-exocytosis of synaptic molecules. This represents the first report of gene silencing in a nematode belonging to the *Longidoridae* family.

## 1. Introduction

*Xiphinema index* is an ectoparasite soil-borne nematode belonging to the *Longidoridae* family. It has been mentioned as 1 of the 10 most economically important plant-parasitic nematodes [1]. This nematode of about 3 mm in length migrates from plant to plant through the soil. It infects roots of grapevine and fig, two economically important crops [2,3]. Large *X. index* populations can be responsible for reducing the plant growth by direct damages on roots [4]. At the cellular level, *X. index* induces the formation of multinucleated cells [5,6] but little is known about the *X. index* effectors responsible for plant parasitism. A few candidates have been identified by sequencing and by in situ hybridization of mRNA extracted from dissected basal bulbs [7]. In addition to direct damage, this nematode is the exclusive vector of *Grapevine fanleaf virus* (GFLV, *Secoviridae* family), one of the most detrimental virus on grapevines causing a fanleaf degeneration disease [8,9,10]. While feeding on an infected grapevine, *X. index* acquires GFLV particles that are retained externally along the alimentary tract at specific sites along the ondotophore, esophagus, and esophageal bulb [11]. Virus retention is long-lasting and *X. index* has the ability to remain viruliferous for several years [12]. Identification of the viral determinants involved in the specific transmission of GFLV particles by *X. index* has greatly benefited from the reconstitution of the atomic structure of the virus combined with reverse genetic experiments on the GFLV coat protein sequence [8,13,14,15,16]. Edges of a depression on the surface of the GFLV capsid are likely the GFLV particles binding site to still unknown receptors in *X. index*.

In order to decipher the molecular interactions responsible for plant parasitism and for virus transmission by *X. index*, it is crucial to have genomic resources and functional validation tools adapted to this nematode species. Genome assemblies and annotations of *X. index* are not yet available but expressed sequence tags (EST) [7], transcriptome, and predicted proteome are available [17]. In contrast, no functional validation technique is available for this organism.

RNA interference (RNAi) represents an attractive approach as a reverse genetics tool, especially when dealing with nematode species refractory to genetic transformation or difficult to culture and manipulate at a genetic level. RNAi has been first developed for the free living nematode *Caenorhabditis elegans* [18] and has then been applied to parasitic nematodes with however some difficulties [19]. The efficacy of RNAi was demonstrated for cyst nematodes like *Globodera pallida* [20,21] and the root-knot nematode *Meloidogyne incognita* [22,23,24] using long dsRNA. Ingestion of small interfering RNA (siRNA) was also shown to be efficient in reducing the expression of target genes in *M. incognita*, *G. pallida*, and *Trichostrongylus colubrifirmis* [25,26,27,28]. Different strategies have been set up to deliver the silencing molecules to the nematodes namely by soaking, by feeding on transgenic plants constitutively expressing dsRNA or on plants infected with a recombinant virus producing the dsRNA in the course of its replication [23,26,27,29,30,31,32,33,34]. These techniques induced silencing of the target genes with some variability in the silencing efficiency between samples and experiments [25]. Despite these significant progresses in functional gene validation in nematodes, the protocols cannot straightforwardly be transferable to *X. index* which belongs to a different phylogenetic clade than the root-knot and cyst nematodes [35]. In order to apply this technology to *X. index*, we evaluated the performance of siRNA in targeting genes expressed in this ectoparasite nematode. A soaking procedure was set up to optimize in vitro acquisition of a fluorescent-siRNA by *X. index*. The efficiency of in vitro acquisition of siRNA on the reduction of the targeted mRNA was evaluated by reverse transcription-quantitative polymerase chain reaction (RT-qPCR). Our results show that siRNA acquisition can induce partial but statistically significant and specific knockdown of the *piccolo* gene in *X. index*. However, expression of two other selected genes, *cysteine rich-venom protein* and *laminin*, was not reproducibly reduced after ingestion of siRNA showing the limitations of the technique for *X. index*. Using comparative proteome annotation strategy, we identified the putative proteins involved in *X. index* silencing pathways. Remarkably, we could not identify all the proteins typically involved in silencing pathways. Especially, neither worm-specific AGO (WAGO) nor RNA-dependent RNA polymerase (RdRP) orthologs were found, which could account for the limited silencing reproducibility in *X. index*.

## 2. Results

### 2.1. Optimization of In Vitro Acquisition of siRNA by X. index

**Figure 1:**
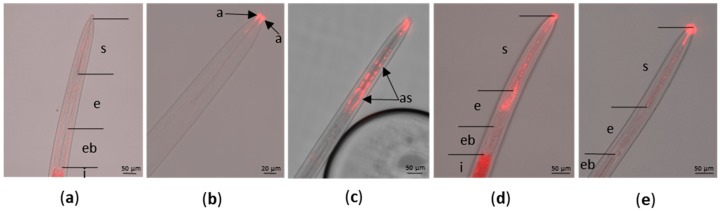
Fluorescence microscopy observations showing the Cy3-siRNA uptake by *X. index*. (**a**) control nematode incubated for 48 h without Cy3-siRNA; (**b**–**e**) nematode soaked with Cy3-siRNA; (**b**–**d**) 48 h incubation; (**e**) 1 h incubation; all the incubations were done in mineral water. a: amphids; s: stylet; e: esophagus; eb: esophageal bulb; i: intestine; as: amphid sheath.

Nematodes in the *Longidoridae* family have never been used for gene functional validation assay. In vitro rearing or in vitro acquisition of exogenous molecules has been thus far unsuccessful [6]. Therefore, the first challenge was to set up an efficient method for the in vitro uptake of siRNA by *X. index*. L4 or adult stages of *X. index* were incubated in an aqueous solution containing a fluorescent siRNA labeled with Cyanine 3 (Cy3) to evaluate both siRNA ingestion and nematode viability in an artificial medium. The nematode alimentary canal starts with the stylet which is composed of two parts: the odontostyle and the odontophore. The odontophore is then followed by the esophagus, the esophageal bulb and finally the intestine (Figure 1a). In the nematode’s head, lateral located sensilla (amphids), representing the largest chemosensory organs of nematodes, are open to the outside. These sensilla are composed of three types of cells including the sheath cell forming a true tube (or channel) starting at the tip of the nematode’s head. The amphids together with the lip region represent entry points for exogenous molecules present in the artificial medium. Following in vitro acquisition of Cy3-siRNA the red fluorescence was visualized predominantly at two different locations: (i) at the tip of the amphid on the border to the stylet aperture (Figure 1b) or extending to the amphid sheath (Figure 1c); or (ii) in the stylet and the esophagus lumen (Figure 1d,e). In contrast, the fluorescence was not reproducibly observed in the esophageal bulb (Figure 1d,e). Labeling of the intestine was uncertain due to autofluorescence observed after incubation of nematodes in a solution deprived of Cy3-siRNA (Figure 1a). These observations suggest two possible, but exclusive, entry routes of the Cy3-siRNA, the amphids or the stylet. siRNA acquisition from the amphids is likely to be more efficient since 1 h after Cy3-siRNA acquisition suspended in mineral water, 45% of the amphid sheath showed fluorescence (Table 1). In contrast, in similar conditions, only 15% of the alimentary tracts were labeled (Table 1; Figure 1e). Extending Cy3-siRNA incubation in mineral water up to 48 h did not strongly increase the number of nematodes labeled (53% of amphid sheath and 23% of alimentary tract labeled) but resulted in a more intense fluorescence of the alimentary tract (Table 1; Figure 1d).

Interestingly, ingestion of siRNA in mineral water had no effect on nematode mobility and viability, even after 48 h of artificial feeding. Addition of reagents in the Cy3-siRNA preparations generally used to improve transfection of animal cells (Cellfectin II or TransIT-insect transfection reagents) had a reverse effect and inhibited the acquisition of the labeled-siRNA (Table 1). Moreover, these compounds had a detrimental effect on the nematode viability when the incubation was extended beyond 1 h (Table 1). These results showed that siRNA can access the lumen of the alimentary track and the inner tissues of L4 or adult stages of *X. index* during soaking in water without any additional compounds.

### 2.2. Selection of X. index Target Genes for RNAi Experiments

**Figure 2:**
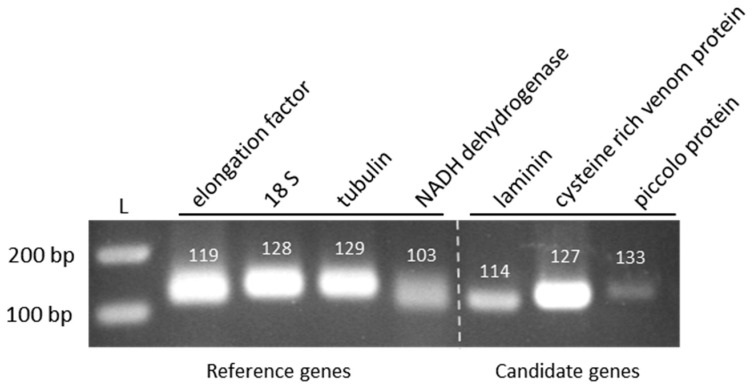
Control of expression of the selected reference and candidate genes in *X. index* by RT-PCR. Amplification of the specific mRNA was performed on total RNA extracted from *X. index*. The amplified DNA fragments were loaded on a 2% agarose gel and visualized by UV after ethidium bromide staining. The expected size of each fragment (in base pair, bp) is mentioned. L: ladder in bp.

Since the complete genome sequence of *X. index* is not yet available, selection of the target genes was based on the available sequences [7,17] that had to meet the following criteria: (i) genes were unique in the available sequences from *X. index*; (ii) genes encoded known proteins and the available gene sequence was over 100 nucleotides. Based on these criteria, three genes were selected that encode the laminin protein, piccolo protein, and a cysteine-rich venom protein (Table S1). No information on the tissue specificity was available for the selected genes. The venom protein is a potential effector produced in secretory organs of root-knot nematodes [36]. Laminins are glycoproteins that form a major polymer within basement membranes [37]. The piccolo protein is involved in synaptic endo/exocytosis but its implication in plant-parasitic nematode interactions is still hypothetical. Specific primers were designed for each gene (Table S2) to control their expression in *X. index* by RT-PCR on total RNA extraction. DNA fragments of expected sizes were obtained and further sequenced to verify specificity of the amplification (Figure 2).

### 2.3. Selection of Reference Genes for Assessing RNAi Efficiency in X. index

To evaluate the efficiency of gene expression inhibition in *X. index* a selection of appropriate reference genes for the RT-qPCR experiments was conducted. The selection first relied on reference genes that had been previously used in RT-qPCR experiments with other nematode species (*tubulin* and *GAPDH* in *M. javanica* [31]; *18S rRNA* in *M. incognita* [38]; *18S rRNA* and *GAPDH* in *M. chitwoodi* [39]; *18S rRNA* and *elongation factor 1 alpha* in *M. javanica* [40]; *GAPDH* in *M. incognita* [25]). Among the four selected reference genes, the *elongation factor*, the *18S rRNA* and the *tubulin* genes exhibit high sequence homology with their counterpart in *M. incognita* or *M. javanica* whereas the NADH dehydrogenase gene of *X. index* was highly divergent from the orthologous genes of *M. incognita* (Table 2 and Table S3).

**Figure 3:**
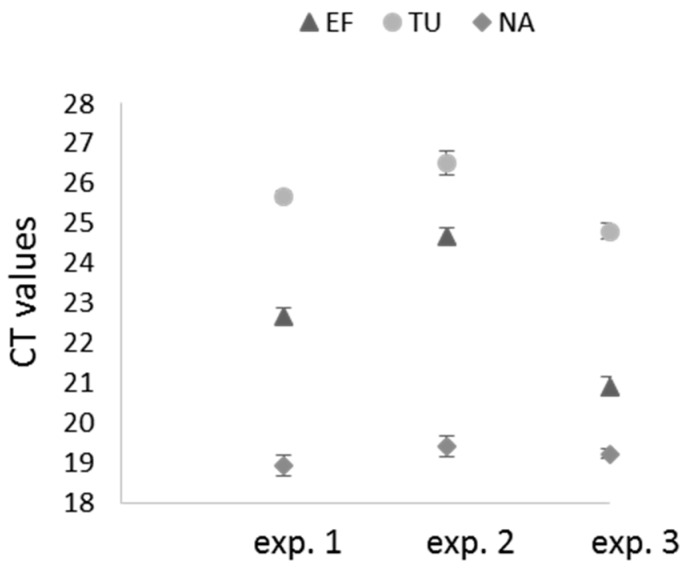
Expression stability of three reference genes in *X. index*. *X. index* genes were tested for their expression stability in three independent RT-qPCR experiments: the *elongation factor* (EF), the *tubulin* (TU), and the *NADH dehydrogenase* (NA) genes. Each point represents the mean of the CT values of 4 pools (Exp. 1 and 3) or 3 pools (Exp. 2) of 30 nematodes. Errors bars represent standard deviation of the mean.

Specific primers were designed for the four potential reference genes (Table S2) and their efficacy in amplifying the specific RNAs was addressed by RT-PCR. As shown in Figure 2, DNA fragments of expected size were amplified from *X. index* total RNA extracts. These DNA fragments were further sequenced to verify the primers specificity. These primers were used in a preliminary RT-qPCR to control the reaction efficiency (E) and the linear correlation coefficient (r^2^). All of them gave expected results (*elongation factor* E = 85.7% and r^2^ = 1; *tubulin* E = 86.3% and r^2^ = 0.997; *NADH dehydrogenase* E = 93.5% and r^2^ = 1) except the primers designed for *18S rRNA* amplification for which PCR reaction efficiency did not reach expectation (E = 69.8% and r^2^ = 1). This potential reference gene was therefore not retained in our analysis. To further evaluate the expression stability of the three remaining potential reference genes, three independent RT-qPCR on 3 to 4 pools of 30 nematodes (mainly at L4 and adult stages) were performed. As shown in Figure 3, the three reference genes were stably expressed between the biological replicates but mRNA expression varied between the three experiments and particularly for the *elongation factor* and the *tubulin* genes. The three reference genes were however included in each RT-qPCR experiment but their stability was always controlled before analyzing the effect of siRNA ingestion on the targeted mRNAs (Figure S1).

### 2.4. Real-Time RT-PCR Analyses of Target Genes in X. index after siRNA Acquisition

Three siRNA were selected for each of the three selected genes, *laminin*, *cystein rich-venom protein*, and *piccolo protein* (Table S4). All siRNA had a sufficient score rate to be used in RT-qPCR analyses. The siRNA targeting the *laminin*-mRNA were referred to as L1, L2, and L3, those targeting the *cystein rich-venom protein*-mRNA as V1, V2, and V3 and those directing against the *piccolo protein*-mRNA as P1, P2, and P3 (Table S4). As control, we used a randomized siRNA with no similarity within the available *X. index* assembled transcriptome (Table S4). To evaluate the effect of the in vitro siRNA ingestion by the nematode on the target mRNA accumulation, batches of 90 *X. index* were soaked in an aqueous solution containing 2 µg of a mixture of the three siRNA designed for each gene for an incubation of 48 h at room temperature. These experimental conditions were previously determined as optimal for fluorescent siRNA uptake (see above) and three independent experiments using siRNA targeting the three candidate genes were performed. Then, total RNA was extracted from a pool of 90 nematodes for each experimental set-up and mRNA accumulation of the target gene was evaluated by RT-qPCR.

**Figure 4:**
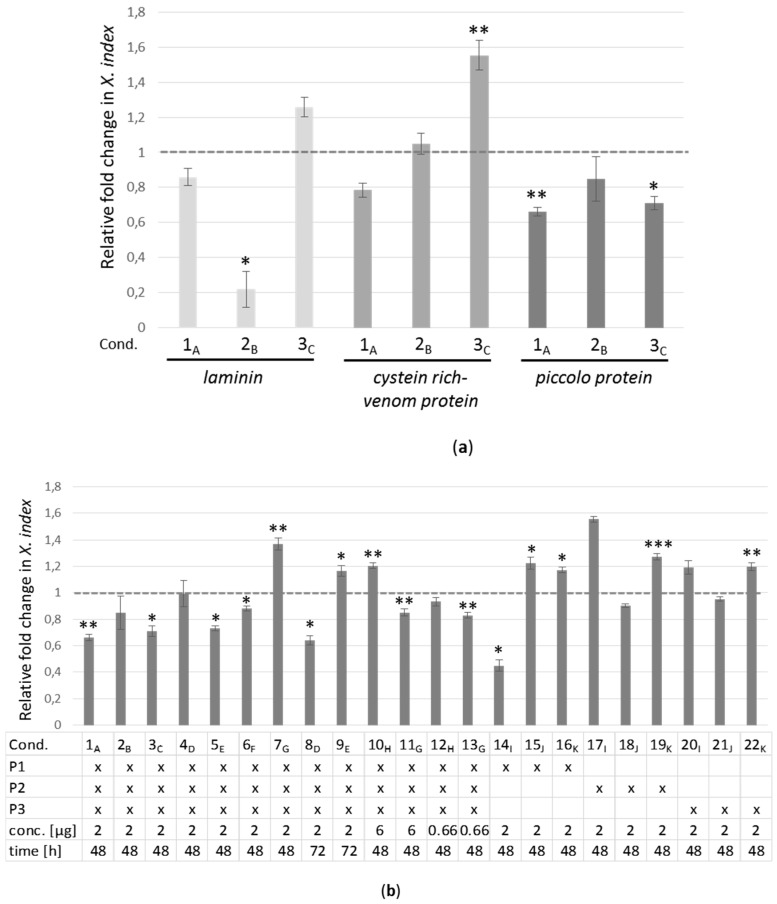
Silencing efficiency of *X. index* targeted genes after siRNA in vitro acquisition. (**a**) Silencing of *laminin*, *cysteine rich venom protein*, and *piccolo protein* genes after siRNA acquisition (2 µg of siRNA) for 48 h. (**b**) Silencing of the *piccolo protein* gene in different experimental conditions (Cond.). The first three conditions are those presented in (a). The conditions varied depending on the siRNA used (P1, P2, and P3 are the three siRNA targeting *piccolo*-mRNA used in mixture or individually), the concentration (conc.) of siRNA used in the artificial medium and the time (h) of incubation with the nematodes. Letters in subscript (A-K) represent independent experiments. Each bar represents the relative fold change of the gene expression in a pool of 90 nematodes ± standard deviation of technical triplicates. Asterisks indicate significant differences between nematodes fed with the negative-control siRNA and nematodes that have acquired specific siRNA molecules (Student’s *t*-test). * *p*-value < 0.05; ** *p*-value < 0.01; *** *p*-value < 0.001. The reference genes stability was controlled in each experiment and is shown in Figure S1.

As shown in Figure 4a, a reduction in the accumulation of the *laminin* mRNA was observed in two out of three experiments (Exp. 1_A_ & 2_B_, Figure 4a) but the observed reduction of the mRNA accumulation was statistically significant in one experiment (~80% reduction in Exp. 2_B_ for laminin, Figure 4a and Table S5). No significant reduction in the accumulation of the *cysteine rich venom protein* mRNA was observed in the three experiments performed and for the *piccolo* candidate gene, a statistically significant reduction of its expression was observed in two out of the first three experiments performed (Exp. 1_A_ and 3_C_; Figure 4a; and Table S5). In order to analyze the reproducibility of the expression inhibition of the *piccolo* gene, the silencing experiment was reproduced 4 more times in similar conditions (2 µg of siRNA and acquisition of 48 h, Exp. 1_A_ to 7_G_; Figure 4b; and Table S5). Overall, a statistically significant inhibition was observed in 4 out of the 7 experiments (Figure 4b, Exp. 1_A_, 3_C_, 5_E_, and 6_F_; and Table S5). In order to see whether a higher and more consistent silencing of the *piccolo* gene could be obtained, the incubation time of the *X. index* in the siRNA solution was extended to 72 h to potentially increase siRNA uptake (Exp. 8_D_ and 9_E_; Figure 4b; and Table S5) and one out of two experiments resulted in a significant decrease of *piccolo*-mRNA accumulation. This suggests that increasing the incubation time does not consistently enhance siRNA internalization into the nematode. The failure to obtain a consistent silencing of the *piccolo* gene was not due to a degradation of the siRNA after a prolonged incubation time as shown in Figure S2. Attempts to elevate the silencing rate of the *piccolo* gene were further addressed by changing the siRNA concentration in the artificial feeding medium (Exp. 10_H_, 11_G_, 12_H_, and 13_G_; Figure 4b; and Table S5). Again, increasing or reducing the concentration of siRNA in the medium did not seem to result in a higher or more consistent reduction of *piccolo*-mRNA accumulation. Lastly, the efficiency of each siRNA taken individually was evaluated on *piccolo* gene silencing. A significant reduction of 55% of the *piccolo*-mRNA accumulation was observed in one experiment when using P1-siRNA alone (Exp. 14_l_; P1 alone; Figure 4b; and Table S5).

Among the 28 experimental conditions used to target the three candidate genes, a significant overexpression of the target gene was also monitored in eight of them (Figure 4a,b and Table S5). This phenomenon may result from a regulatory mechanism to counteract the action of the siRNA. In all the different experimental set-up, a high viability of the nematodes was observed (Table S5).

Thus, the gene silencing experiments performed showed that a consistent and reproducible silencing could not be obtained for the three selected candidate genes. However, a reduction of the expression of the *piccolo protein*-mRNA ranging from −12 % to −55% was obtained in 8 out of 22 experimental conditions (representing 11 independent experiments) using a single or a mixture of three siRNA.

### 2.5. Annotation of X. index Putative Proteins Involved in Gene Silencing

Because gene silencing experiments were not consistent and reproducible for all the three selected candidates, we questioned the wholeness and/or the upkeep of the gene silencing regulatory machinery in *X. index*. To this end we looked in *X. index* predicted proteome for the main proteins involved in the biogenesis routes of the three major families of small non-coding RNAs (small ncRNA): microRNAs (miRNA), small interfering RNAs (siRNA), and Piwi-interacting RNAs (piRNA). Despite their different origins, these small ncRNA that play a pivotal role in the gene silencing machinery, share specific steps in their biogenesis pathways. They are key regulators of genomic output either at the transcriptional or at the post-transcriptional levels. Regulatory control by small ncRNA requires two main groups of proteins: (i) enzymes with nuclease activity able to excise small RNAs from specific RNA transcripts; and (ii) a diverse cohort of RNA-binding proteins responsible for the stabilization, transport, and regulatory activity of the small ncRNA [41]. To identify these proteins in *X. index*, we combined OrthoFinder groupings with the presence of Argonaute domains (PF02170 and PF02171) since Argonaute protein family is a major protein player of the gene silencing pathway [42]. Based on orthology with *C. elegans* annotated proteins, a total of 28 putative proteins potentially involved in the miRNA, siRNA, or piRNA pathways were identified in *X. index* (Table 3 and Table S6, Figure S3). The functional annotation was completed with the identification of Argonaute domains in 12 additional *X. index* proteins that however did not group with *C. elegans* proteins (Table S6). Orthologous sequences of AGO proteins, with the two miRNA-specific Argonautes, ALG-1 and ALG-2, the ERI/DICER complex (ERI-1/3/5 and DCR-1), PASH-1 (processing of pri-miRNA), PRG-1 (member of the Piwi clade of AGO), and Xrn-2 (a ribonuclease) were identified in the predicted proteome of *X. index*. However, siRNA main components (ERGO-1; RRF-1/EGO-1; CSR-1 and RdRP) were noticeably absent in this analysis of *X. index* predicted proteome. Moreover, no WAGO nor Sid-1 and -2, involved in systemic RNA interference, were identified in *X. index* predicted proteome by this analysis.

## 3. Discussion

*X. index* is an economically important plant parasitic nematode for which limited genomic resources is available and no functional validation tool. Here, we addressed whether RNAi-based techniques could be applied to *X. index* to specifically inhibit gene expression. We observed that soaking nematodes with siRNA targeting specific genes resulted in expression inhibition with however some limitations: the reduction of expression could not be observed for all the candidate genes. The RNAi silencing efficiency can be influenced by the level of transcripts accumulation, by the mRNA stability and turn-over, and by the secondary structure of the RNA which can impair hybridization of the siRNA. It is therefore easy to understand why all expressed genes will not be silenced in the same extent. Moreover, the site of gene expression can also impact gene silencing efficiency and genes expressed in sites accessible to the external environment (like the intestine, amphids, and excretory cells) are potentially more efficiently targeted by RNAi as was demonstrated in *Haemonchus contortus* and *Schistosoma mansoni* [43,44]. For amphids, the specific labelling after ingestion of fluorescent tracer was reported for cyst and root nematodes [21,23]. Another limitation of the technique was an important variability between experiments. Non-reproducible silencing has already been reported in several studies [43,45]. Despite the lack of extensive studies to unravel the source of variability in the amplitude of nematode response to RNAi silencing, some assumptions can already be made: the silencing efficiency relies on a fully efficient RNAi pathway which had not yet been described for *X. index*. We have addressed whether the essential genes of the silencing pathways could be found in *X. index* assembled transcriptome. We have shown that AGO genes (ALG-1/2 and ALG-3/4 for instance) and Dicer (DCR-1) are present whereas no WAGO orthologs could be identified. The lack of WAGO in nematode species other than *C. elegans* has already been reported and it has been suggested that WAGO might be important for the specific lifestyle of particular species [46]. In addition, no RdRP orthologs were identified in *X. index* predicted proteome. RdRP have been shown to catalyze the biogenesis of abundant secondary siRNAs using the target mRNA as template. In the nematode model *C. elegans*, gene inactivation by RNAi achieves remarkable potency due to the amplification by RdRP of the initial silencing [47,48]. The silencing efficiency is also impacted by the uptake and the systemic movement of the RNAi molecules which relies on the transmembrane proteins SID-1 and SID-2 [49,50]. Although SID-1 protein sequences seem to be conserved in different nematode species, neither proteins were found in *X. index* predicted proteome [51,52]. It is possible that other proteins are fulfilling the function of SID proteins for the uptake of dsRNA. Another reason for inconsistent silencing could be attributed to the expression of RNAi inhibitory genes like the *ERI-1* and *Xrn-2* genes in *C. elegans* [53,54]. Orthologous sequences of these former proteins were found in *X. index* predicted proteome. The comparative proteome analysis revealed that not all essential genes in the silencing pathways found in other nematode species were identified in *X. index*. Incomplete transcriptome or/and divergence in sequence identity of orthologous proteins may explain the lack of identification but alternately it is conceivable that *X. index* is not fully equipped for RNAi resulting in a low and non-reproducible silencing efficiency.

We observed in our study that siRNA uptake by *X. index* resulted in some cases in significant overexpression of the targeted genes. This phenomenon has already been mentioned in several studies after acquisition of dsRNA [25,27,43,55] or siRNA by plant parasitic nematodes [30,56,57]. This may reflect homeostatic feedback and should be particularly the case for genes encoding transcription factors that are tightly regulated by sophisticated mechanisms.

RNAi in *X. index* still requires adaptations before being used for large scale reverse genetics of genes involved in plant parasitism or in virus transmission. However, beside its application in functional validation studies, RNAi is also a promising approach to control plant parasitic nematodes through the expression of nematode-specific dsRNA in transgenic plants targeting essential genes [31,32,34,58,59]. It has already been shown that *in planta* silencing of 16D10 a candidate effector from *M. incognita* could be applied to protect crop plants such as potatoes [39,60] and transgenic grape hairy roots [61]. This technique may therefore provide opportunities to develop new environmentally friendly approaches to fight against nematodes and limit their capacity to transmit viruses. These innovative strategies could replace the chemical nematicides that have been banned for toxicity reasons.

Another alternative to conduct functional validation in *X. index* would be to apply the CRISPR/Cas9 technology to edit the genome. This technology has been successfully developed on *C. elegans* and other non-model nematodes to knockout gene expression [62,63]. However, at present, no data is available on the feasibility of this strategy in plant parasitic nematodes, particularly because of the lack of a genetic transformation system.

## 4. Materials and Methods

### 4.1. Nematode Rearing

*X. index* were reared on fig trees (*Ficus carica*) or grapevines (*Vitis vinifera*) under controlled greenhouse conditions [14]. Nematodes were extracted from soil samples using the sieving method described by Flegg [64], counted and isolated under a binocular microscope.

### 4.2. siRNA Design and Synthesis

The siRNAs were designed following the Silencer siRNA Construction Kit instructions (Ambion, Austin, TX, USA). One important criteria was the presence of the dinucleotide (UU) at the 3′ siRNA extremity. These siRNA are supposed to be more effective than the unmodified siRNA [65]. Three online software products were used to select the target sequence and the specific siRNA. RNAfold server tool on http://rna.tbi.univie.ac.at/ was used to predict minimum free energy structures and secondary structures of targeted mRNA. The targeted sequence belongs to a region for which the MFE (minimum free energy) was high. Starting from the identified sequence, siRNA were designed using RNAxs server tool (http://rna.tbi.univie.ac.at/) and siDirect2 (http://sidirect2.rnai.jp/). The siRNA with more than 16 contiguous base pair identity with sequences in the *X. index* assembled transcriptome were eliminated. Twenty-one nucleotide-long siRNA were synthesized using the Silencer siRNA Construction Kit (Ambion, Austin, TX, USA) following the instructions. Briefly, a pair of complementary oligonucleotide templates were first synthesized corresponding to 19 nucleotides of the target sequence, the dinucleotide and 8-nucleotide leader sequence. The leader sequence of each template enabled the hybridization of a T7 promoter-related sequence which was extended by the Klenow DNA Polymerase to produce two dsDNA containing promoter sequences located at opposite ends. The transcription of these dsDNA molecules by the T7 RNA Polymerase resulted in the synthesis of complementary RNA molecules of 19 nucleotides with 3′ overhanging UU dinucleotides referred thereafter as siRNA. Three siRNA were designed for each candidate gene. One negative-control siRNA was designed with no similarity within the *X. index* genome. siRNA sequences used in this study are listed in Table S1. To optimize the conditions for the in vitro ingestion of siRNA by *X. index*, a synthetic oligonucleotide labeled with Cyanine 3 (Cy3) was used (block-it alexa fluor red fluorescent control, ThermoFisher Scientific, Waltham, Massachusetts, MA, USA).

### 4.3. Nematode Soaking

Adults or L4 larva stages of *X. index* were isolated from soil samples and were incubated with siRNA as followed: pools of 30 *X. index* were incubated with 2 µL Cy3-siRNA (20 µM) in 200 µL of mineral water for 1 h, 24 h, or 48 h at room temperature in darkness to set up the conditions for siRNA ingestion by the nematodes. Cellfectin II reagent (Thermofisher) or TransIT-insect transfection reagent (Mirus) was eventually added to the siRNA solution. After soaking, the nematodes were washed three times with mineral water. Fluorescent Cy3-siRNA uptake was controlled using Axio imager M2 microscope (Zeiss, Marly-le-Roi, France) with appropriate filters. For the RNA silencing experiments, pools of 90 *X. index* were incubated into siRNA solutions prepared in RNase free water and ranging from 3 ng/µL to 30 ng/µL for 24 h, 48 h, or 72 h. After soaking, the nematodes were washed four times with mineral water. Nematode viability and motility were controlled under a binocular.

### 4.4. RNA Extraction and Quantitative RT-PCR (RT-qPCR)

Total RNA was extracted from pools of 30 or 90 nematodes using an RNeasy minikit (Qiagen, Hilden, Germany). The RNA extracts were treated with RQ1 RNase-free DNase I (Promega Corporation, Madison, WI, USA). RNA was quantified at 260 nm with the Nanodrop 2000 (Thermo Fisher Scientific, Waltham, MA, USA). First-strand cDNA was synthesized from 8 ng to 20 ng of RNA using Moloney murine leukemia virus (MMLV) reverse transcriptase (Promega Corporation, Madison, WI, USA) according to the manufacturer’s instructions, with oligo(dT)_18_ as the primer. The quantitative PCR (qPCR) reaction was performed using GoTaq Flexi DNA Polymerase (Promega Corporation, Madison, WI, USA) and specific primers designed by Beacon Designer 3 software (Biosoft, Palo Alto, CA, USA) (Table S2). RT-PCR products were loaded on a 2% agarose gel and visualized by UV after ethidium bromide staining. The PCR-amplified DNA products were sequenced to control primers specificity.

The RT-qPCR analyses were performed, in triplicate in 96-well optical plates. Each plate systematically contained the three reference genes samples and the negative control samples together with the target gene samples. Each reaction was performed after mixing 8 to 20 ng of cDNA with 0.8 µL of each primer at 10 mM, and 10 µL of SYBR^TM^ Green Supermix (Bio-rad, Hercules, CA, USA) in a final volume of 20 µL. The RT-qPCR reactions, conducted on a CFX cycler (Bio-rad, Hercules, CA, USA), were initiated with a 3 min incubation at 95 °C followed by 40 cycles of amplification (10 s at 95 °C, 30 s at 60 °C). Melt curve analysis was performed from 60 °C to 95 °C with 5 s of 0.5 °C increments. Threshold cycle (CT) values were calculated using Bio-rad CFX Manager^TM^ software (Bio-rad, Hercules, CA, USA). Expression levels were normalized with three reference genes encoding elongation factor, tubulin, and NADH dehydrogenase from *X. index*. The primer specificity was assessed by melting curves analyses of PCR products. Amplification efficiency was determined by a 5-fold dilution series of cDNA (from 1/5 to 1/3125) corresponding to reference and target genes. Both analyses were conducted using Bio-rad CFX Manager^TM^ software. The relative expression levels were calculated using the ∆∆CT method [66]. The results were analysed for significant differences with Student’s *t*-test.

### 4.5. Comparative Functional Annotation

For comparative annotation of putative proteins involved in *X. index* silencing pathways, we selected a total of 11 other nematode species from publicly available protein databases, including 9 plant-parasitic nematodes, some of which proteomes were already functionally annotated (Table S7). Because *X. index* genome assemblies are not yet available, we based the annotations on *X. index* protein sequences translated from transcriptome assembly provided by Danchin et al. [17]. The model species, *C. elegans*, was used as a reference because of its genome completeness, annotation quality and the existence of experimental characterization of the silencing pathways. *X. index* proteins were identified thanks to a custom pipeline consisting in the identification of both (i) orthology links between the 12 nematode proteomes by using OrthoFinder version 1.1.4 under standard parameters (for instance, MLC inflation parameter [Default = 1.5]) [67] and (ii) identification of specific PfamA [68] Argonaute protein domains (PF02170 and PF02171) assigned by Interproscan [69] to explore and analyse homology clusters we used a web server called Family-Companion [70].

## Figures and Tables

**Table 1:** Efficiency of Cy3-siRNA uptake by *X. index* after in vitro acquisition

Cy3-siRNA Solution	Acquisition Period	% of Viable Nematodes	Labeling of Amphid Sheath ^1^	Labeling of Alimentary Tract ^1^
Mineral water	1 h	100%	9/20 (45%)	3/20 (15%)
	24 h	100%	16/30 (53%)	6/30 (20%)
	48 h	100%	16/30 (53%)	7/30 (23%)
Mineral water + Cellfectin II	1 h	100%	0/26 (0%)	0/26 (0%)
	24 h	100%	0/29 (0%)	0/29 (0%)
	48 h	50%	0/26 (0%)	0/26 (0%)
Mineral water + TransIT-insect transfection reagent	1 h	100%	0/23 (0%)	0/23 (0%)
	24 h	10%	0/20 (0%)	0/20 (0%)
	48 h	0%	nd ^2^	nd

^1^ numbers represent nematodes in which fluorescence was observed/total number of nematodes observed. In brackets, percentage of fluorescent nematodes.^2^ nd, not determined.

**Table 2:** Selection of reference genes for RNAi experiments in *X. index*.

Reference Genes	Number of EST in *X. index*	% of Sequence Identity with Orthologous Sequences in Root-Knot Nematodes
*tubulin*	19	70 to 75% ^1^
*elongation factor*	2	81% ^1^
*18S*	10	70 to 76% ^1^
*NADH dehydrogenase*	40	nd ^2^

^1^ range of sequence identity between the *X. index* EST and the EST from the root-knot nematode *M. javanica*. ^2^ nd, not determined because of high divergent sequences between *X. index* and *M. incognita*.

**Table 3:** Identification of *X. index* putative proteins involved in gene silencing.

Name of Family or Complex Involved in *C. Elegans* Gene Silencing	Name ^1^	*C. elegans* Wormbase Accession Number ^2^	Presence/Absence of Putative *X. index* Proteins in Ortholog Group ^3^
AGO	ALG-1	WBGene00000105	Presence of AGO ortholog
	ALG-2	WBGene00000106	
	HPO-24/ALG-5	WBGene00011945	
	ALG-3	WBGene00011910	
	ALG-4	WBGene00006449	
	RDE-1	WBGene00004323	
	ZK218.8	WBGene00013942	
PIWI	ERGO-1	WBGene00019971	No *X. index* putative prot.
	PRG-1	WBGene00004178	Presence of PRG-1 ortholog
WAGO	WAGO-1	WBGene00011061	No *X. index* putative prot.
	WAGO-2	WBGene00018862	
	WAGO-4	WBGene00010263	
	WAGO-5	WBGene00022877	
	SAGO-1	WBGene00019666	
	SAGO-2	WBGene00018921	
	PPW-1	WBGene00004093	
	PPW-2	WBGene00004094	
	HRDE-1	WBGene00007624	
	HRDE-2	WBGene00011324	
	NRDE-3	WBGene00019862	
	WAGO-10	WBGene00020707	
	WAGO-11	WBGene00021711	
	C14B1.7	WBGene00007578	
	CSR-1	WBGene00017641	
	C04F12.1	WBGene00007297	
ERI/DICER complex	ERI-1	WBGene00001332	Presence of ERI ortholog
	ERI-3	WBGene00021103	
	ERI-5	WBGene00021419	
	DCR-1 (Dicer)	WBGene00000939	Presence of DCR-1 ortholog
DICER related complex	PASH1	WBGene00011908	Presence of PASH1 ortholog
	DRH1	WBGene00001090	No *X. index* putative prot.
	DRH3	WBGene00008400	
	DRSH1 (Drosha)	WBGene00009163	
RdRP	RRF-3	WBGene00004510	No *X. index* putative prot.
	EGO-1	WBGene00001214	
	RRF-1	WBGene00004508	
	RRF-2	WBGene00004509	
Systemic RNA interference defective	Sid-1	WBGene00004795	No *X. index* putative prot.
	Sid-2	WBGene00004796	
ribonuclease	Xrn-2	WBGene00006964	Presence of Xrn-2 ortholog

^1^ names of genes are indicated for the nematode model species, *C. elegans*. ^2^ wormbase accession numbers of protein coding genes. ^3^ Presence/absence of ortholog in *X. index* proteome, predicted from transcriptomic data, is indicated for each family or complex known to be involved in *C. elegans* gene silencing.

## Supplementary Materials

Supplementary materials can be found at https://www.mdpi.com/1422-0067/20/13/3266/s1. Figure S1: Stability of the three reference genes used in the RT-qPCR analyses in *X. index*; Figure S2: Over time stability of siRNA used to inhibit *piccolo* gene expression in *X. index*; Figure S3: Alignments of putative proteins involved in gene silencing; Table S1: Selection of candidate genes for RNAi experiments in *X. index*; Table S2: Primer sequences used for the RT-qPCR analysis; Table S3: Sequences in *X. index* displaying sequence homology with orthologous sequences in *M. javanica* or *M. incognita*; Table S4: Primer sequences used for siRNA synthesis; Table S5: Silencing efficiency of *laminin*, *cysteine rich venom protein*, or *piccolo protein* genes in *X. index*; Table S6: List of *X. index* putative proteins involved in gene silencing; Table S7: List of the 12 species used for functional comparative annotation of *X. index* putative proteins involved in gene silencing.

## Abbreviations

RNAi	RNA interference
RT-qPCR	Reverse transcription quantitative polymerase chain reaction
WAGO	Worm-specific argonaute
RdRP	RNA dependent RNA polymerase
Cy3	Cyanine 3
