# Supplementary material for: In Vitro Acquisition of Specific Small Interfering RNAs Inhibits the Expression of Some Target Genes in the Plant Ectoparasite Xiphinema index
Source: Int J Mol Sci. 2019 Jul 3;20(13):3266. doi: 10.3390/ijms20133266 (PMC6651894; doi:10.3390/ijms20133266)
Supplement: Supplementary file 1 [file ijms-20-03266-s001.zip › Figure S1 Marmonier IJMS revised MS.pptx]

## Slide 1
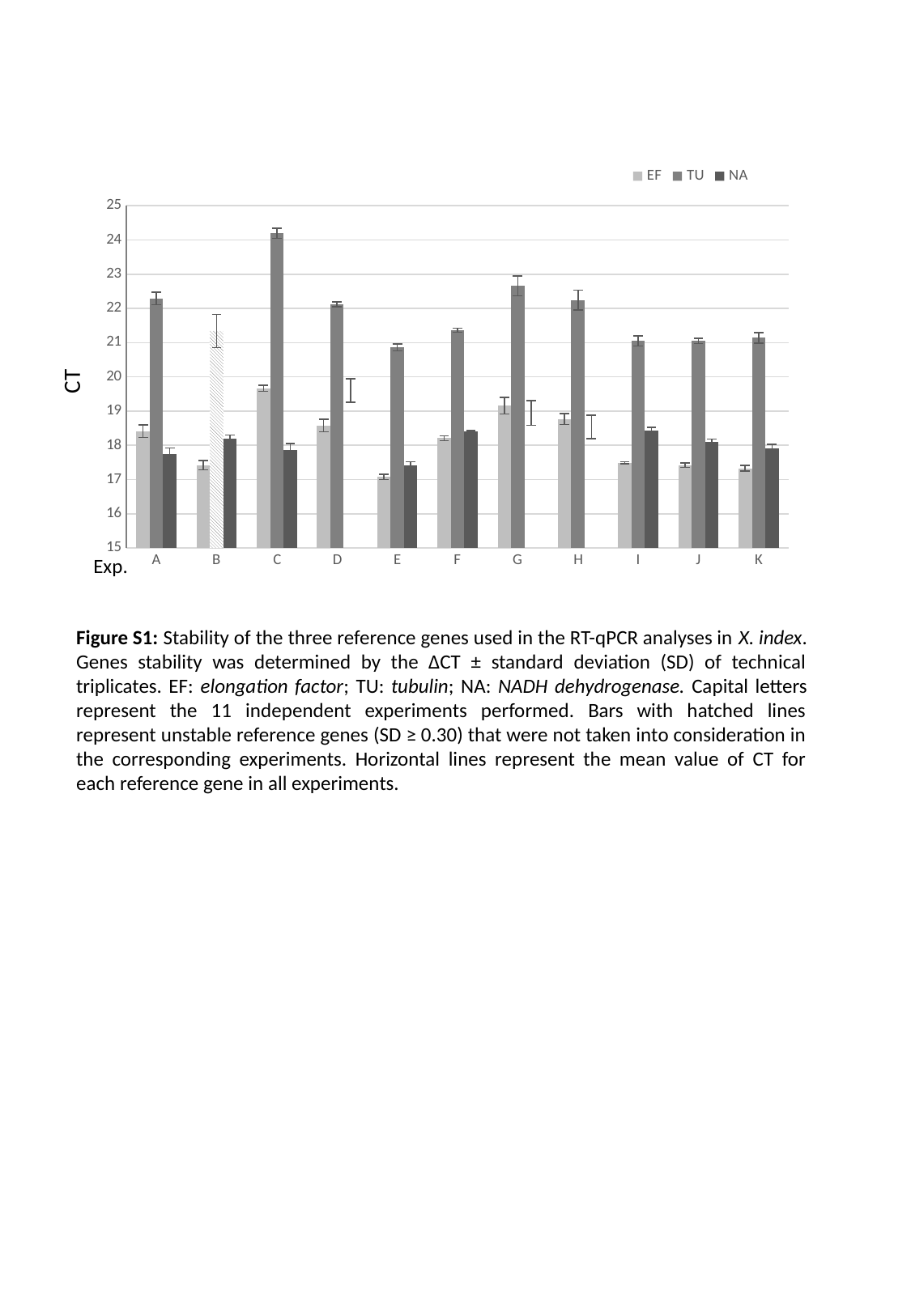

### Chart
| Category | EF | TU | NA |
|---|---|---|---|
| A | 18.41 | 22.29 | 17.75 |
| B | 17.42 | 21.34 | 18.19 |
| C | 19.67 | 24.19 | 17.86 |
| D | 18.58 | 22.12 | 19.6 |
| E | 17.08 | 20.86 | 17.41 |
| F | 18.21 | 21.36 | 18.4 |
| G | 19.16 | 22.66 | 18.94 |
| H | 18.77 | 22.24 | 18.54 |
| I | 17.49 | 21.05 | 18.43 |
| J | 17.42 | 21.05 | 18.1 |
| K | 17.33 | 21.14 | 17.91 |CT
Exp.
Figure S1: Stability of the three reference genes used in the RT-qPCR analyses in X. index. Genes stability was determined by the ∆CT ± standard deviation (SD) of technical triplicates. EF: elongation factor; TU: tubulin; NA: NADH dehydrogenase. Capital letters represent the 11 independent experiments performed. Bars with hatched lines represent unstable reference genes (SD ≥ 0.30) that were not taken into consideration in the corresponding experiments. Horizontal lines represent the mean value of CT for each reference gene in all experiments.
