# Supplementary material for: In Vitro Acquisition of Specific Small Interfering RNAs Inhibits the Expression of Some Target Genes in the Plant Ectoparasite Xiphinema index
Source: Int J Mol Sci. 2019 Jul 3;20(13):3266. doi: 10.3390/ijms20133266 (PMC6651894; doi:10.3390/ijms20133266)
Supplement: Supplementary file 1 [file ijms-20-03266-s001.zip › Figure S2 Marmonier IJMS revised MS.pptx]

## Slide 1
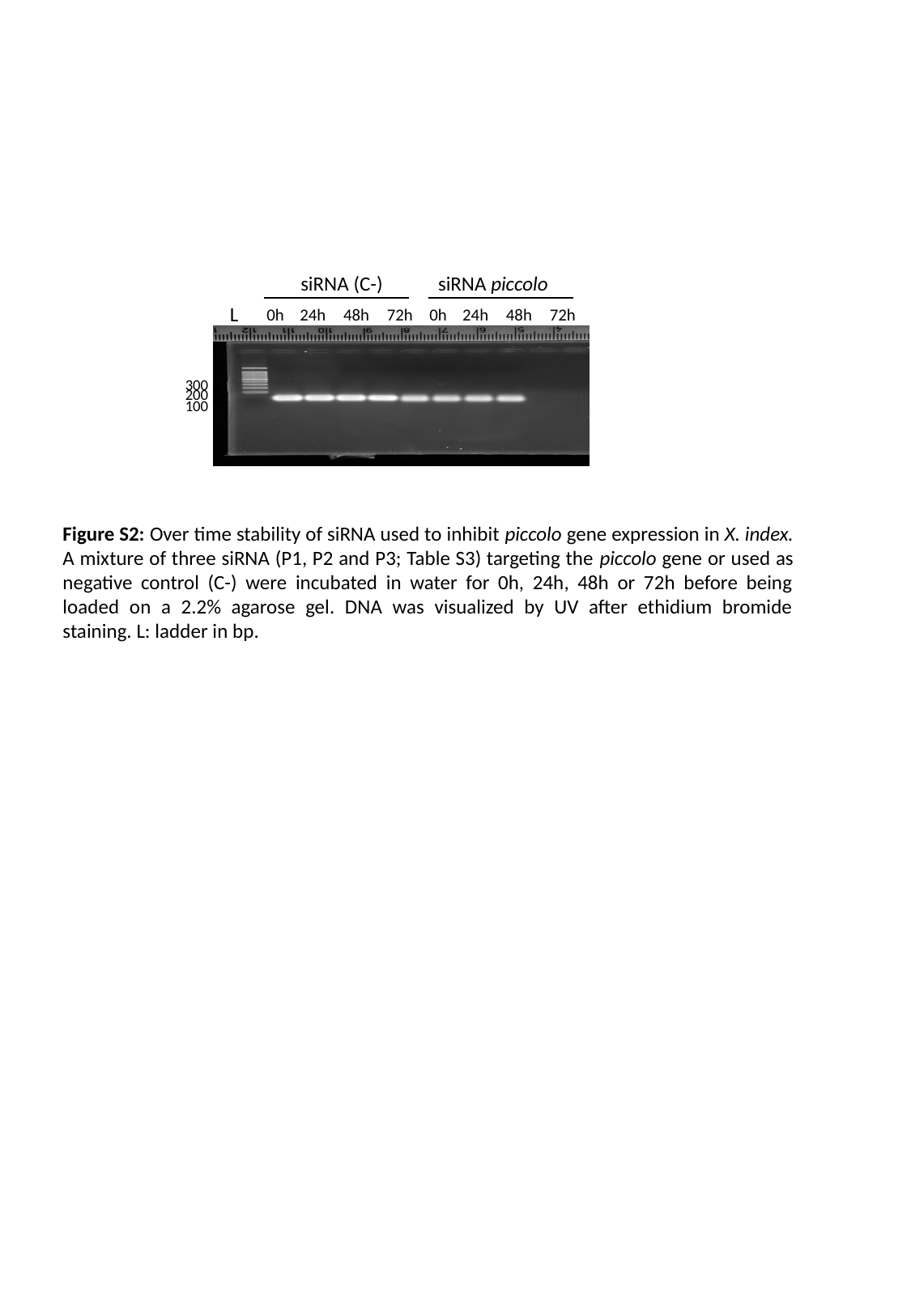

siRNA (C-)
siRNA piccolo
L
0h
24h
48h
72h
0h
24h
48h
72h
300
200
100
Figure S2: Over time stability of siRNA used to inhibit piccolo gene expression in X. index. A mixture of three siRNA (P1, P2 and P3; Table S3) targeting the piccolo gene or used as negative control (C-) were incubated in water for 0h, 24h, 48h or 72h before being loaded on a 2.2% agarose gel. DNA was visualized by UV after ethidium bromide staining. L: ladder in bp.
