# Supplementary material for: In Vitro Acquisition of Specific Small Interfering RNAs Inhibits the Expression of Some Target Genes in the Plant Ectoparasite Xiphinema index
Source: Int J Mol Sci. 2019 Jul 3;20(13):3266. doi: 10.3390/ijms20133266 (PMC6651894; doi:10.3390/ijms20133266)
Supplement: Supplementary file 1 [file ijms-20-03266-s001.zip › Figure S3 Marmonier IJMS revised MS.docx]

**Figure S3:** Alignments of putative proteins involved in gene silencing. Alignment obtained with CLUSTAL O(1.2.4) multiple sequence alignment.

AGO

ALG1_WBGene00000105_Celegans MSGGPQYLPGVMNSTIQQQPQSATSSFLPSGPISSTSTS-SQVVPTSGATQQPPFPSAQA

ALG2_Cel_WBGene00000106_Celegans ------------------------------------------------------------

HPO24_WBGene00011945_Celegans -------------------------------------------------------MEDQW

ALG3_WBGene000119108Celegans ------------------MSRRNATNFVDNNTLTSS---GSG--S-----LSPPITSRPA

ALG4_WBGene00006449_Celegans ------------------MSRRNATSFVDNNTLTSSGISGSG--S-----MSPPITSRPA

RDE1_WBGene00004323_Celegans ------------------------------------------------------------

ZK218.8l_WBGene00013942_Celegans ------------------------------------------------------------

Mhap1s0264g09247_Mhapla ------------------------------------------------------------

Mhap1s0000g00097_Mhapla ------------------------------------------------------------

XIPH00220_m.1024_Xindex ------------------------------------------------------------

XIPH14718_m.27510_Xindex ------------------------------------------------------------

XIPH03015_m.8656_Xindex ------------------------------------------------------------

XIPH03061_m.8764_Xindex ------------------------------------------------------------

XIPH17950_m.31169_Xindex ------------------------------------------------------------

XIPH03137_m.8937_Xindex ------------------------------------------------------------

XIPH00171_m.824_Xindex ------------------------------------------------------------

XIPH13915_m.26514_Xindex ------------------------------------------------------------

XIPH01240_m.4301_Xindex ------------------------------------------------------------

XIPH28640_m.40226_Xindex ------------------------------------------------------------

XIPH00682_m.2641_Xindex ------------------------------------------------------------

XIPH01763_m.5725_Xindex ------------------------------------------------------------

XIPH01058_m.3772_Xindex ------------------------------------------------------------

XIPH01422_m.4808_Xindex ------------------------------------------------------------

XIPH11895_m.23873_Xindex ------------------------------------------------------------

XIPH11139_m.22857_Xindex ------------------------------------------------------------

XIPH05696_m.14049_Xindex ------------------------------------------------------------

XIPH00358_m.1579_Xindex ---------------------------MSSDDKKAGGRRGAGRGRRQTPAQEPPIPSRGI

XIPH00282_m.1279_Xindex ----------------------------MADSSDNGGSS---------------------

ALG1_WBGene00000105_Celegans AASTALQNDLEEIFNSPPTQPQTFSDVPQRQAGSLAPGVPIG-NTSV-SIGEPANTLGGG

ALG2_Cel_WBGene00000106_Celegans ------------MFPLPVHNGPRLGKLSI----FEMPGDSLT-SSSFMPDGGAETSSSSQ

HPO24_WBGene00011945_Celegans LLSAIYDDDLVEKLKVRSST-------SSRSTSINVPS---L-ENEFLSS-----SSGSR

ALG3_WBGene000119108Celegans ---------------------S-------------GQASPLSSNGSLSPPV--DDQGSVS

ALG4_WBGene00006449_Celegans ---------------------S-------------GQASPLTSNGSLSPPQYADDQGSVS

RDE1_WBGene00004323_Celegans ----MSS-----NF---------------------------------------PE-----

ZK218.8l_WBGene00013942_Celegans ------------------------------------------------------------

Mhap1s0264g09247_Mhapla ------------------------------------------------------------

Mhap1s0000g00097_Mhapla ------------------------------------------------------------

XIPH00220_m.1024_Xindex ------------------------------------------------------------

XIPH14718_m.27510_Xindex ------------------------------------------------------------

XIPH03015_m.8656_Xindex ------------------------------------------------------------

XIPH03061_m.8764_Xindex ------------------------------------------------------------

XIPH17950_m.31169_Xindex ------------------------------------------------------------

XIPH03137_m.8937_Xindex ------------------------------------------------------------

XIPH00171_m.824_Xindex ------------------------------------------------------------

XIPH13915_m.26514_Xindex ------------------------------------------------------------

XIPH01240_m.4301_Xindex ------------------------------------------------------------

XIPH28640_m.40226_Xindex ------------------------------------------------------------

XIPH00682_m.2641_Xindex ------------------------------------------------------------

XIPH01763_m.5725_Xindex ------------------------------------------------------------

XIPH01058_m.3772_Xindex ------------------------------------------------------------

XIPH01422_m.4808_Xindex ------------------------------------------------------------

XIPH11895_m.23873_Xindex ------------------------------------------------------------

XIPH11139_m.22857_Xindex ------------------------------------------------------------

XIPH05696_m.14049_Xindex ------------------------------------------------------------

XIPH00358_m.1579_Xindex PESPMAP-----SVPGEIAGGR-------------GRGRAVVTEPQIAPSEGSPE-----

XIPH00282_m.1279_Xindex ------------------PMGR-------------GRGRGVRRDKAGTRPGGNDTSSSVT

ALG1_WBGene00000105_Celegans LPGGAPG------QLPGGNQSGIQFQCPRRPNHGV----EGRSILLRANHFAVRIPG--G

ALG2_Cel_WBGene00000106_Celegans LGGSAHG------AIGTKPDAGVQFQCPVRPNHGV----EGRSILLRANHFAVRIPG--G

HPO24_WBGene00011945_Celegans V--SDDL------YLHPIEENREPFKLIGKPLPST----TGRFLSLLANHFQITCNG--S

ALG3_WBGene000119108Celegans YNSDSPRDLSP--LLLSELACLNMREVVARPGLGT----IGRKIPVKSNFFAVDLKNPKM

ALG4_WBGene00006449_Celegans YNLDSPRDLSP--LLLSELACLNMREVVARPGLGT----IGRQIPVKSNFFAMDLKNPKM

RDE1_WBGene00004323_Celegans L----EKGF-YRHSLDPEMK------WLARPTGKCDGKFYEKKVLLLVNWFKFSSKIYDR

ZK218.8l_WBGene00013942_Celegans ------------------------------------------------------------

Mhap1s0264g09247_Mhapla ------------------------------------------------------------

Mhap1s0000g00097_Mhapla ------------------------------------------------------------

XIPH00220_m.1024_Xindex ------------------------------------------------------------

XIPH14718_m.27510_Xindex ------------------------------------------------------------

XIPH03015_m.8656_Xindex ------------------------------------------------------------

XIPH03061_m.8764_Xindex ------------------------------------------------------------

XIPH17950_m.31169_Xindex ------------------------------------------------------------

XIPH03137_m.8937_Xindex ------------------------------------------------------------

XIPH00171_m.824_Xindex ------------------------------------------------------------

XIPH13915_m.26514_Xindex ------------------------------------------------------------

XIPH01240_m.4301_Xindex ------------------------------------------------------------

XIPH28640_m.40226_Xindex ------------------------------------------------------------

XIPH00682_m.2641_Xindex ------------------------------------------------------------

XIPH01763_m.5725_Xindex ------------------------------------------------------------

XIPH01058_m.3772_Xindex ------------------------------------------------------------

XIPH01422_m.4808_Xindex ------------------------------------------------------------

XIPH11895_m.23873_Xindex ------------------------------------------------------------

XIPH11139_m.22857_Xindex ------------------------------------------------------------

XIPH05696_m.14049_Xindex ------------------------------------------------------------

XIPH00358_m.1579_Xindex --AGSPKGSPSGSDVDDKMKNLEIIGLAPRPGFGV----SGNKTQVLANYFEVAMQK-EL

XIPH00282_m.1279_Xindex VQGGGSSASSTTVRSESSAKTHFSDTLVKRPGFGA----AGRKVNLRTNNFVINCSG-SL

ALG1_WBGene00000105_Celegans TIQHYQVDVTPDKCPRR----------VNR----EIISCLISAFSKYF--TNIRPVYDGK

ALG2_Cel_WBGene00000106_Celegans SVQHYQIDVFPDKCPRR----------VNR----EVIGCLISSFSKYF--TNIRPVYDGK

HPO24_WBGene00011945_Celegans IIHQYYIRFDPDIPSKK----------LNR----TILRTLQEQNPGLI---ECPLVFDGI

ALG3_WBGene000119108Celegans VVVQYHVEVHHPGC-RK----------LDKDEMRIIFWKAVSDHPNIFH-NKFALAYDGA

ALG4_WBGene00006449_Celegans VVIQYHVEIHHPGC-RK----------LDKDEMRIIFWKAVSDHPNIFH-NKFALAYDGA

RDE1_WBGene00004323_Celegans EYYEYEVKMTKEVLNRKPGKPFPKKTEIPIPDRAKLFWQHLRHEKKQTDFILEDYVFDEK

ZK218.8l_WBGene00013942_Celegans ------------------------------------------------------------

Mhap1s0264g09247_Mhapla --------------------------------------------MNLV--VCQLPVYDGK

Mhap1s0000g00097_Mhapla ----------PDKCPRK----------VNR----EIVNTMVDS-CKLF--TGFKPVYDGK

XIPH00220_m.1024_Xindex ---HYEINVSPEKCPRR----------VNR----EIVSTMVRAYNRIF--NNLRPVYDGK

XIPH14718_m.27510_Xindex ------------------------------------------------------------

XIPH03015_m.8656_Xindex ------------------------------------------------------------

XIPH03061_m.8764_Xindex ------------------------------------------------------------

XIPH17950_m.31169_Xindex ------------------------------------------------------------

XIPH03137_m.8937_Xindex ------------------------------------------------------------

XIPH00171_m.824_Xindex ------------------------------------------------------------

XIPH13915_m.26514_Xindex ------------------------------------------------------------

XIPH01240_m.4301_Xindex ------------------------------------------------------------

XIPH28640_m.40226_Xindex ------------------------------------------------------------

XIPH00682_m.2641_Xindex ---HYEINVSPEKCPRR----------VNR----EIVSTMVRAYNRIF--NNLRPVYDGK

XIPH01763_m.5725_Xindex ------------------------------------------------------------

XIPH01058_m.3772_Xindex ------------------------------------------------------------

XIPH01422_m.4808_Xindex ------------------------------------------------------------

XIPH11895_m.23873_Xindex ------------------------------------------------------------

XIPH11139_m.22857_Xindex ------------------------------------------------------------

XIPH05696_m.14049_Xindex ------------------------------------------------------------

XIPH00358_m.1579_Xindex TVTQYRVEVKHERM--K----------LNRDEKQRIFWTCVRENPTVFQ-NHTRLVYDGE

XIPH00282_m.1279_Xindex KLHQYQIDISHEFRQKK----------LKKEDSYIIFFELL---KTLVT-DKYSVLYDGG

ALG1_WBGene00000105_Celegans RNMYTREPLPIGRERMDFDVT-------LPGDS------AVERQFSVSLKWVGQVSLSTL

ALG2_Cel_WBGene00000106_Celegans RNMYTREPLPIGTEPMNFEVT-------LPGDS------AVERKFSVTMKWIGQVCLSAL

HPO24_WBGene00011945_Celegans HTVYSTELINVKEVNNSV-IN-------VAGVV---NTKESPNLFKLYLTHVDSFLLD--

ALG3_WBGene000119108Celegans HQLYTVARLEFPDDQGSVRL---DCEATLPKDNRDRT------RCAISIQNVGPVLLEMQ

ALG4_WBGene00006449_Celegans HQLYTVARLEFPDDQGSVRL---DCEASLPKDNRDRT------RCAISIQNVGPVLLEMQ

RDE1_WBGene00004323_Celegans DTVYSVCRLNTVTSKMLVSEKV----VKKDSEKKDEKDLEKKILYTMILTYRKKFHLNFS

ZK218.8l_WBGene00013942_Celegans ------------------------------------------------------------

Mhap1s0264g09247_Mhapla KNLYTKEALLFGQERIELDVV-------M-RDS------AVDRKFRVALKPC---SLQAL

Mhap1s0000g00097_Mhapla KNLYTKEALPFGQERIELDVV-------MPGDS------AVDRKFRVALKLVSRVSLQAL

XIPH00220_m.1024_Xindex KNMYTRDPLPIGKERMELEVT-------LPGDS------AVERQFRVSIKWVSQVSLTLL

XIPH14718_m.27510_Xindex ------------------------------------------------------------

XIPH03015_m.8656_Xindex ------------------------------------------------------------

XIPH03061_m.8764_Xindex ------------------------------------------------------------

XIPH17950_m.31169_Xindex ------------------------------------------------------------

XIPH03137_m.8937_Xindex ------------------------------------------------------------

XIPH00171_m.824_Xindex ------------------------------------------------------------

XIPH13915_m.26514_Xindex ---------------------LFQVKLRETSV--LVRDNNKQYYFTLEVKHVSAITVDDV

XIPH01240_m.4301_Xindex ------------------------------------------------------------

XIPH28640_m.40226_Xindex ------------------------------------------------------------

XIPH00682_m.2641_Xindex KNMYTRDPLPIGKERMELEVT-------LPGDS------AVERQFRVSIKWVSQVSLTLL

XIPH01763_m.5725_Xindex ------------------------------------------------------------

XIPH01058_m.3772_Xindex ------------------------------------------------------------

XIPH01422_m.4808_Xindex ------------------------------------------------------------

XIPH11895_m.23873_Xindex ------------------------------------------------------------

XIPH11139_m.22857_Xindex ------------------------------------------------------------

XIPH05696_m.14049_Xindex ------------------------------------------------------------

XIPH00358_m.1579_Xindex SICASLDRLTFGNGPGSDVGGTYEIRIRLNRMDRPE-------PMKVIFSGTGLIHLNMR

XIPH00282_m.1279_Xindex YICFSLIPFNLKD------QNVFTVRVSLPSYDRPS-------DYKVSIKETIKPQFDLR

ALG1_WBGene00000105_Celegans EDAMEGRV-RQVPFEAVQAMDVILRHL--------------PSLKYTPVGRSFFSPPVPN

ALG2_Cel_WBGene00000106_Celegans DDAMEGRV-RQVPHEAVQSIDVILRHL--------------PSLKYTPVGRSFFTPPGVM

HPO24_WBGene00011945_Celegans TKIITGNQDQNQKLRMMHAIDTVFRQT--------------STGNFHAVLQSFFSIAQNS

ALG3_WBGene000119108Celegans RTRT-NNLD-ERVLTPIQILDIICRQSLTCPLLK------NS-ANFYTWKSSCYRIPT--

ALG4_WBGene00006449_Celegans RTRT-NNLD-ERVLTPIQILDIICRQSLTCPLLK------NS-ANFYTWKSSCYRIPT--

RDE1_WBGene00004323_Celegans REN--PEKD-EEANRSYKFLKNVMTQKVRYAPFVNEEIKVQFAKNFVYDNNSILRVPESF

ZK218.8l_WBGene00013942_Celegans ------------------------------------------------------------

Mhap1s0264g09247_Mhapla EDAMAG------------------------------------------LGRSFFSLTVSG

Mhap1s0000g00097_Mhapla EDAMAGRI-RQIPPESVQAMDVILRHL--------------PSMKYTPVGRSFFSSPPLA

XIPH00220_m.1024_Xindex EEAMEGRV-RTIPYESVQAMDVILRHL--------------PSLKFTPVGRSFFSPPAG-

XIPH14718_m.27510_Xindex ------------------------------------------------------------

XIPH03015_m.8656_Xindex ------------------------------------------------------------

XIPH03061_m.8764_Xindex ------------------------------------------------------------

XIPH17950_m.31169_Xindex ------------------------------------------------------------

XIPH03137_m.8937_Xindex ------------------------------------------------------------

XIPH00171_m.824_Xindex ------------------------------------------------------------

XIPH13915_m.26514_Xindex RQAYNENLN-ERERQQIQFLELLFRQGRY------------FNDHLIPAGRSFYMRAH--

XIPH01240_m.4301_Xindex ------------------------------------------------------------

XIPH28640_m.40226_Xindex -------------------MDVILRHL--------------PSLKYTPVGRSFFSPPTP-

XIPH00682_m.2641_Xindex EEAMEGRV-RTIPYESVQAMDVILRHL--------------PSLKYTPVGRSFFSPPT--

XIPH01763_m.5725_Xindex ------------------------------------------------------------

XIPH01058_m.3772_Xindex ------------------------------------------------------------

XIPH01422_m.4808_Xindex ------------------------------------------------------------

XIPH11895_m.23873_Xindex ------------------------------------------------------------

XIPH11139_m.22857_Xindex ------------------------------------------------------------

XIPH05696_m.14049_Xindex ------------------------------------------------------------

XIPH00358_m.1579_Xindex EVRSAGGTG-DRYTTPLQVFDLIMRQRRTYLLDE------VTAREWFTFANTVYKIPP--

XIPH00282_m.1279_Xindex DTSSMGK------QSTLAVLDLVFRQWYNLPAAE------DAPKKFYPFGRSFFYPTS--

ALG1_WBGene00000105_Celegans ASGVMAGSCPPQASGAVAGGAHSAGQYHAESKLGGGREVWFGFHQSVRPSQ-WKMMLNID

ALG2_Cel_WBGene00000106_Celegans KPGM---------------------QMHQESKLGGGREVWFGFHQSVRPSQ-WKMMLNID

HPO24_WBGene00011945_Celegans AI---------EPSHG---------LGWGTVNLGVGREVCYGFYQNVVETF-DTLTMNLD

ALG3_WBGene000119108Celegans -------------------------AAGQALDLEGGKEMWTGFFSSAHIASNYRPLLNID

ALG4_WBGene00006449_Celegans -------------------------AAGQALDLEGGKEMWTGFFSSAHIASNYRPLLNID

RDE1_WBGene00004323_Celegans HD---------------------PNRFEQSLEVAPRIEAWFGIYIGIKELFDGEPVLNFA

ZK218.8l_WBGene00013942_Celegans ------------------------------------------------------------

Mhap1s0264g09247_Mhapla -----------------------------GVGLVGGREVCF-------------------

Mhap1s0000g00097_Mhapla L-----GAQHITVSGGIGGGGGGPQGVDKLGGLGGGREVWFGFHQSVRPSQ-WKMMLNID

XIPH00220_m.1024_Xindex --------------------------MYPDSKLGGGREVWFGFHQSVRPSQ-WKMMLNID

XIPH14718_m.27510_Xindex ------------------------------------------------------------

XIPH03015_m.8656_Xindex ------------------------------------------------------------

XIPH03061_m.8764_Xindex ------------------------------------------------------------

XIPH17950_m.31169_Xindex ------------------------------------------------------------

XIPH03137_m.8937_Xindex ------------------------------------------------------------

XIPH00171_m.824_Xindex ------------------------------------------------------------

XIPH13915_m.26514_Xindex --------------------------KNDSLDLGCGRELLRGFYLSTRLIDNWQLSLNVD

XIPH01240_m.4301_Xindex ------------------------------------------------------------

XIPH28640_m.40226_Xindex ---------------------------YPDSKLGGGREVWFGFHQSVRPSQ-WKMMLNID

XIPH00682_m.2641_Xindex --------------------------PYPDSKLGGGREVWFGFHQSVRPSQ-WKMMLNID

XIPH01763_m.5725_Xindex ------------------------------------------------------------

XIPH01058_m.3772_Xindex ------------------------------------------------------------

XIPH01422_m.4808_Xindex ------------------------------------------------------------

XIPH11895_m.23873_Xindex ------------------------------------------------------------

XIPH11139_m.22857_Xindex ------------------------------------------------------------

XIPH05696_m.14049_Xindex ------------------------------------------------------------

XIPH00358_m.1579_Xindex --------------------------GGRGPDLTCGRVIWQGLFSAARVGQGYTPYINFD

XIPH00282_m.1279_Xindex ------------------------VGDRLGFDLDAGRQVWGGYFYGVKWGANSVPIINID

ALG1_WBGene00000105_Celegans VSATAFYR--SMPVIEFIAEVLELPVQALAE-----------------------------

ALG2_Cel_WBGene00000106_Celegans VSATAFYR--AMPVIEFVAEVLELPVQALAE-----------------------------

HPO24_WBGene00011945_Celegans VATTTFYR--PVALVEFLAEILEVPLATVTD-----------------------------

ALG3_WBGene000119108Celegans VAHTAFYK-TRITVLQFMCDVLNERTSKPNRNNPRGPG---APGGYRGGRGGARGGSYQN

ALG4_WBGene00006449_Celegans VAHTAFYK-TRITVLQFMCDVLNERTSKPNRNNPRGPGGPGGPGGYRGGRGGGRGGSYGN

RDE1_WBGene00004323_Celegans IVDKLFYNAPKMSLLDYLLLIVDPQSCNDDVRKDLKTKLMAGKMTI--------------

ZK218.8l_WBGene00013942_Celegans ------------------------------------------------------------

Mhap1s0264g09247_Mhapla --CTAFYR--KMPVINFMAEVLELPMQALND-----------------------------

Mhap1s0000g00097_Mhapla VSATAFYR--KMPVINFMAEVLELPMQALND-----------------------------

XIPH00220_m.1024_Xindex VSATAFYR--SMPVIEFMAEVLELPMAGLND-----------------------------

XIPH14718_m.27510_Xindex ------------------------------------------------------------

XIPH03015_m.8656_Xindex ------------------------------------------------------------

XIPH03061_m.8764_Xindex ------------------------------------------------------------

XIPH17950_m.31169_Xindex ------------------------------------------------------------

XIPH03137_m.8937_Xindex ------------------------------------------------------------

XIPH00171_m.824_Xindex ------------------------------------------------------------

XIPH13915_m.26514_Xindex WNVTAFYE--PEHLIEFAAKFLTAYYKQRYNPHDEKP-----------------------

XIPH01240_m.4301_Xindex ------------------------------------------------------------

XIPH28640_m.40226_Xindex VSATAFYR--SMPVIEFMAEVLELPMAGLND-----------------------------

XIPH00682_m.2641_Xindex VSATAFYR--SMAVTEFLAEVLEMPMGQLND-----------------------------

XIPH01763_m.5725_Xindex ------------------------------------------------------------

XIPH01058_m.3772_Xindex ------------------------------------------------------------

XIPH01422_m.4808_Xindex -----------------------------ND-----------------------------

XIPH11895_m.23873_Xindex ------------------------------------------------------------

XIPH11139_m.22857_Xindex ------------------------------------------------------------

XIPH05696_m.14049_Xindex ------------------------------------------------------------

XIPH00358_m.1579_Xindex VSHSGFYK-PQ-SVLEYMVDILNATQVNPRDRHAT-------------------------

XIPH00282_m.1279_Xindex VANSVFIK-KQ-SVLQFMIDVFNQETGKPL------------------------------

ALG1_WBGene00000105_Celegans -----------------------------RRAL--SDAQRVKFTKEIRGLKIEITHCG--

ALG2_Cel_WBGene00000106_Celegans -----------------------------RRAL--SDAQRVKFTKEIRGLKIEITHCG--

HPO24_WBGene00011945_Celegans -----------------------------GRSL--SDVQKKKFNREVAGLKVETRHCS--

ALG3_WBGene000119108Celegans FGNRGPPGANVRDDFGGNGLTFTMDTLSRDTQL--SSFETRIFGDSIRGMKIRATHRP--

ALG4_WBGene00006449_Celegans FGNRGPPGANVRDDFGGNGLTFTMDTLSRDTQL--SSFETRIFGDAIRGMKIRAAHRP--

RDE1_WBGene00004323_Celegans ---RQAA-------------RPRIRQLLENLKLKCAEVWDN----EMSR-----------

ZK218.8l_WBGene00013942_Celegans ------------------------------------------------------------

Mhap1s0264g09247_Mhapla -----------------------------RRNL--SDLQRVKFTKEIRGLKIEITHCG--

Mhap1s0000g00097_Mhapla -----------------------------RRNL--SDPQRVKFTKEIRGLKIEITHCG--

XIPH00220_m.1024_Xindex -----------------------------RRPL--SDAQRVKFTKEIRGLKIEITHCG--

XIPH14718_m.27510_Xindex ------------------------------------------------------------

XIPH03015_m.8656_Xindex ------------------------------------------------------------

XIPH03061_m.8764_Xindex ------------------------------------------------------------

XIPH17950_m.31169_Xindex ------------------------------------------------------------

XIPH03137_m.8937_Xindex ------------------------------------------------------------

XIPH00171_m.824_Xindex ------------------------------------------------------------

XIPH13915_m.26514_Xindex --------------------------------PNLTDDCRRLLERELKDVKFITDHTN--

XIPH01240_m.4301_Xindex ------------------------------------------------------------

XIPH28640_m.40226_Xindex -----------------------------RRPL--SDAQRVKFTKEIRGLKIEITHCG--

XIPH00682_m.2641_Xindex -----------------------------RRPL--SDAQRVKFTKEIRGLKIEITHCG--

XIPH01763_m.5725_Xindex ------------------------------------------------------------

XIPH01058_m.3772_Xindex ------------------------------------------------------------

XIPH01422_m.4808_Xindex -----------------------------RRPL--SDAQRVKFTKEIRGLKIEITHCG--

XIPH11895_m.23873_Xindex ------------------------------------------------------------

XIPH11139_m.22857_Xindex ------------------------------------------------------------

XIPH05696_m.14049_Xindex ------------------------------------------------------------

XIPH00358_m.1579_Xindex --------------------MFNVNQLDQNTSL--QQHWLLPVDKGMRGLKVRMQVRKVD

XIPH00282_m.1279_Xindex ---------------------YSMEQLKPSAPFTMSPQQLQILTKAIKGLLVTTK--TV-

ALG1_WBGene00000105_Celegans --QMRRKYRVCNVTRRPAQTQTFPLQLETGQ---TIECTVAKYFYDKYRIQLKYPHLPCL

ALG2_Cel_WBGene00000106_Celegans --AVRRKYRVCNVTRRPAQTQTFPLQLETGQ---TIECTVAKYFFDKYRIQLKYPHLPCL

HPO24_WBGene00011945_Celegans --CP-RRFRVARCTWKPTENISFHLSETAGN-QDSKPLSLVEYYKRRYNIDLTYKHLPCI

ALG3_WBGene000119108Celegans --NAIRVYKVNSL-QLPADKLMFQGIDEEGR---QVVCSVADYFSEKYG-PLKYPKLPCL

ALG4_WBGene00006449_Celegans --NAIRVYKVNSL-QLPADKLMFQGIDEEGR---QVVCSVADYFSEKYG-PLKYPKLPCL

RDE1_WBGene00004323_Celegans --LTERHLTFLDLCEENSLVYKVTGKSDRGRNAKKYDTTLFKIYEENKK-FIEFPHLPLV

ZK218.8l_WBGene00013942_Celegans ------------------------------------------------------------

Mhap1s0264g09247_Mhapla --QMRRKYRVCNVTRKPAQTQTFIFL----------------------------------

Mhap1s0000g00097_Mhapla --QMRRKYRVCNVTRKPAQTQTFPLLLENGL---SIDCTVLKYFNDKYHMQLKYPHLPCL

XIPH00220_m.1024_Xindex --TMRRKYRVCNVTRRPAQTQTFPLQLENGQ---TIECTVAKYFFDKYQMQLKYPHLPCL

XIPH14718_m.27510_Xindex ------------------------------------------------------------

XIPH03015_m.8656_Xindex ------------------------------------------------------------

XIPH03061_m.8764_Xindex ----------DNVTRRPAQTQTFPLQLENGQ---TIECTVAKYFFDKYHMQLKYPHLPCL

XIPH17950_m.31169_Xindex ------------------------------------------------------------

XIPH03137_m.8937_Xindex ------------------------------------------------------------

XIPH00171_m.824_Xindex ------------------------------------------------------------

XIPH13915_m.26514_Xindex -----ETFKPAVFAAATARTQQFHWEDRDID---HGMVTIEEYFKRRYNRQLRYPGWPLL

XIPH01240_m.4301_Xindex ------------------------------------------------------------

XIPH28640_m.40226_Xindex --TMRRKYRVCNVTRRPAQTQTRSEERFSRN---AE------------------------

XIPH00682_m.2641_Xindex --TMRRKYRVCNVTRRPAQTQTFPLQLENGQ---TIECTVAKYFFDKYHMQLKYPHLPCL

XIPH01763_m.5725_Xindex -------------------------------------CTVAKYFFDKYKMKLKYTNLPCL

XIPH01058_m.3772_Xindex ------------------------------------------------------------

XIPH01422_m.4808_Xindex --TMRRKYRVCNVTRRPAQTQTFPLQLENGQ---TIECTVAKYFFDKYQMQLKYPHLPCL

XIPH11895_m.23873_Xindex ------------------------------------------------------------

XIPH11139_m.22857_Xindex ------------------------------------------------------------

XIPH05696_m.14049_Xindex ------------------------------------------------------------

XIPH00358_m.1579_Xindex GELMEREYTVNNVVKDTATTHSFPQRQPDGT---ERSITVATYFAERYG-PLRYPNLQLL

XIPH00282_m.1279_Xindex ---SGRAQKVLSVTTQSAEKMTFEWKQREGE---PRTINVAEYFSQRYG-NLRLPFLPCL

ALG1_WBGene00000105_Celegans QVGQEQKHTYLPPEVCNIV-PGQRCIK-KLTDVQTSTMIKATARSAPEREREISNLVRKA

ALG2_Cel_WBGene00000106_Celegans QVGQEQKHTYLPPEVCDIV-PGQRCLK-KLTDVQTSTMIKATARSAPEREREICKLVSKA

HPO24_WBGene00011945_Celegans EVGRTR-ECILPLELCYVV-SGQRCIK-KLNEQQIANLIRATSRNATERQNAVMSLQNRL

ALG3_WBGene000119108Celegans HVGPPTRNIFLPMEHCLID-SPQKYNK-KMTEKQTSAIIKAAAVDATQREDRIKQLAAQA

ALG4_WBGene00006449_Celegans HVGPPTRNIFLPMEHCLID-SPQKYNK-KMSEKQTSAIIKAAAVDATQREDRIKQLAAQA

RDE1_WBGene00004323_Celegans KVKSGAKEYAVPMEHLEVHEKPQRYKN-RIDLVMQDKFLKRATRKPHDYKENTLKMLKEL

ZK218.8l_WBGene00013942_Celegans ------------------------------------------------------------

Mhap1s0264g09247_Mhapla ------------------------------------------------------------

Mhap1s0000g00097_Mhapla QVGQEQKHTYLPLEVCEIV-SGQRCIK-KLSDTQTSTMIKVTARNAPDREKEISALVRRA

XIPH00220_m.1024_Xindex QVGQEQKHTYLPPEVCNIV-PGQRCIK-KLTDTQTSTMIRATARSAPEREREIANLVRKA

XIPH14718_m.27510_Xindex ------------------------------------------------------------

XIPH03015_m.8656_Xindex ------------------------------------------------------------

XIPH03061_m.8764_Xindex QVGQEQKHTYLPPEVCNIV-PGQRCIK-KLTDTQTSTMIRATARTAPEREREIANLVRKA

XIPH17950_m.31169_Xindex ------------------------------------------------------------

XIPH03137_m.8937_Xindex ------------------------------------------------------------

XIPH00171_m.824_Xindex ------------------------------------------------------------

XIPH13915_m.26514_Xindex QRGKGQNVRYTPMEVCTIA-PGQRVKNKDITDDQHKTMVLQTAVTPFQRRLNIL------

XIPH01240_m.4301_Xindex ------------------------------------------------------------

XIPH28640_m.40226_Xindex ------------------------------------------------------------

XIPH00682_m.2641_Xindex QVGQEQKHTYLPPEVCNIV-PGQRCIK-KLTDTQTSTMIRATARTAPEREREIANLVRKA

XIPH01763_m.5725_Xindex QVGQEQKHTYLPLEVCNIV-PGQRCLK-KLSDTQTSTMIKATARSAPDRELDINELVKRA

XIPH01058_m.3772_Xindex ------------------------------------------------------------

XIPH01422_m.4808_Xindex QVGQEQKHTYLPPEVCNIV-PGQRCIK-KLTDTQTSTMIRATARSAPEREREIANLVRKA

XIPH11895_m.23873_Xindex ------------------------------------------------------------

XIPH11139_m.22857_Xindex ------------------------------------------------------------

XIPH05696_m.14049_Xindex ------------------------------------------------------------

XIPH00358_m.1579_Xindex HVGPRNKKCYVPMEHCTLA-YNQRVR--KLNPTETSIMIKAAAIDAPTRKGKINDLVKQS

XIPH00282_m.1279_Xindex EVGSKRKVAYLPLEVCEMY-EPQRVAG-RLTEAQTSAMIKGVCTDATEREARIRNVIQKA

ALG1_WBGene00000105_Celegans EFS--ADPFAHEFGITINPAMTEVKGRVLSAPKLLYGGRT----RA-TALPNQGVWDMRG

ALG2_Cel_WBGene00000106_Celegans ELS--ADPFAHEFGITINPAMTEVKGRVLSAPKLLYGGRH----RATTALPNQGVWDMRG

HPO24_WBGene00011945_Celegans KMD--NDVNAVKFGLKVEAQLLKIEGRVLPVPRLLYRSPNLK-RQECVTVPNNGTWDMRG

ALG3_WBGene000119108Celegans SFG--TDPFLKEFGVAVSSQMIETSARVIQPPPIMFGGN--NRSINPVVFPKDGSWSMDH

ALG4_WBGene00006449_Celegans SFG--TDPFLKEFGVAVSSQMIQTTARVIQPPPIMFGGN--NRSVNPVVFPKDGSWTMDN

RDE1_WBGene00004323_Celegans DFSSEELNFVERFGLCSKLQMIECPGKVLKEPMLVNSVNEQI-KMTPVI----RGFQEKQ

ZK218.8l_WBGene00013942_Celegans ------------------------------------------------------------

Mhap1s0264g09247_Mhapla ------------------------------------------------------------

Mhap1s0000g00097_Mhapla EIV--HDPFAQEFGISINSNMTEVKGRVLNAPKLLYGGRT----KA-TALPNQGVWDMRG

XIPH00220_m.1024_Xindex EFN--NDPFAHEFGIAINQAMTEVKGRVLVAPKLLYGGRT----KA-TAVPSQGVWDMRG

XIPH14718_m.27510_Xindex ------------------------------------------------------------

XIPH03015_m.8656_Xindex -------------GVSVDMKMIQFNGRVLDAPVVEYSGRK-------SLIPDTGEWNMRG

XIPH03061_m.8764_Xindex EFN--NDPFAHEFGIAINQTMTEVKGRVLVAPKLLYGGRT----KA-TAVPSLGVWDMRG

XIPH17950_m.31169_Xindex ------------------------------------------------------------

XIPH03137_m.8937_Xindex -------------GVSVDMKMIQFNGRVLDAPVVEYSGRK-------SLIPDTGEWNMRG

XIPH00171_m.824_Xindex ---------------------------------------------------------MRG

XIPH13915_m.26514_Xindex ------------------------------------------------------------

XIPH01240_m.4301_Xindex ------------------------------------------------------------

XIPH28640_m.40226_Xindex ------------------------------------------------------------

XIPH00682_m.2641_Xindex EFN--NDPFAHEFGIAINQTMTEVKGRVLVAPKLLYGGRT----KA-TAVPSLGVWDMRG

XIPH01763_m.5725_Xindex DFN--NDPFAREFGIEVSPTMAEVYGRVLAPPKLLYGGRT----RQ-TATPSKGVWDMRG

XIPH01058_m.3772_Xindex ---------------------------------------------------------MRG

XIPH01422_m.4808_Xindex EFN--NDPFAHEFGIAINQAMTEVKGRVLVAPKLLYGGRT----KA-TAVPSQGVWDMRG

XIPH11895_m.23873_Xindex ------------------------------------------------------------

XIPH11139_m.22857_Xindex ------------------------------------------------------------

XIPH05696_m.14049_Xindex ------------------------------------------------------------

XIPH00358_m.1579_Xindex NFV--QDAWLQQFGVRVNTEMKKVDARVLQPPKIQYAQGGGGGRQGPIAEVRDGKWDMRG

XIPH00282_m.1279_Xindex DLD--RDPFAQKFGVQISLKMMETQGRVMTAPSLEYAGSA-------RVSPRDGVWNMGQ

ALG1_WBGene00000105_Celegans KQFHT--GIDVRVWAIACFAQQQHVKENDLRMFTNQLQRISNDAGMPIVGNPC----FCK

ALG2_Cel_WBGene00000106_Celegans KQFHT--GMEVRTWAIACFAQQSHVKENDLRMFTTQLQRISTDAGMPIIGTPM----FCK

HPO24_WBGene00011945_Celegans KNFYS--GIQIREWAIVCFASPEIIGEASMRSFVRNLVNVASEIGMPFLEEHR----FCR

ALG3_WBGene000119108Celegans QTLYM--PATCRSYSMI--ALVDPRDQTSLQTFCQSLTMKATAMGMNFPR-WP---DLVK

ALG4_WBGene00006449_Celegans QTLYM--PATCRSYSMI--ALVDPRDQTSLQTFCQSLTMKATAMGMNFPR-WP---DLVK

RDE1_WBGene00004323_Celegans LNVVPEKELCCAVFVVNETAGNPCLEENDVVKFYTELIGGCKFRGIRIGANENRGAQSIM

ZK218.8l_WBGene00013942_Celegans ------------------------------------------------------------

Mhap1s0264g09247_Mhapla ------------------------------------------------------------

Mhap1s0000g00097_Mhapla KQFHT--GIEVKTWAIACFAQQNHVKEADLRNFTGHLQKISADAGMPIQGQPC----FCK

XIPH00220_m.1024_Xindex KQFHT--GVEIKVWAIACFAQQQHVKDSDLRGFTAQLQRISSDAGMPVIGQPC----FCK

XIPH14718_m.27510_Xindex ------------------------------------------------------------

XIPH03015_m.8656_Xindex TKVIR--GCEIGDWAVL--CADMNMDEHQVQEFCQGLVRIGGAMNVHFRSPRP---FHIE

XIPH03061_m.8764_Xindex KQFHT--GVEIKVWAIACFAQQQHVKDSDLRSFTAQLQRISNDAGMPVIGQPC----FCK

XIPH17950_m.31169_Xindex ------------------------------------------------------------

XIPH03137_m.8937_Xindex TKVIR--GCEIGDWAVL--CADMNMDEHQVQEFCQGLVRIGGAMNVHFRSPRP---FHIE

XIPH00171_m.824_Xindex KQFHT--GVEIKVWAIACFAQQQHVKDSDLRGFTAQLQRISSDAGMPVIGQPC----FCK

XIPH13915_m.26514_Xindex ------------------------------------------------------------

XIPH01240_m.4301_Xindex ------------------------------------------------------------

XIPH28640_m.40226_Xindex ------------------------------------------------------------

XIPH00682_m.2641_Xindex KQFHT--GVEIKVWAIACFAQQQHVKDSDLRSFTAQLQRISNDAGMPVIGQPC----FCK

XIPH01763_m.5725_Xindex KQFHT--GVEIRYWAIACFAQQFHCREDSIRNFTQMLQKVSNDAGMPITGQPC----FCK

XIPH01058_m.3772_Xindex KQFHT--GVEIKVWAIACFAQQQHVKDSDLRGFTAQLQRISSDAGMPVIGQPC----FCK

XIPH01422_m.4808_Xindex KQFHT--GVEIKVWAIACFAQQQHVKDSDLRGFTAQLQRISSDAGMPVIGQPC----FCK

XIPH11895_m.23873_Xindex ------------------------------------------------------------

XIPH11139_m.22857_Xindex ------------------------------------------------------------

XIPH05696_m.14049_Xindex ------------------------------------------------------------

XIPH00358_m.1579_Xindex QKFYL--GASCNKIAAL--ITEDRDIPAY-MDFFGFLLQTCREFGMNVPANIH---QSAV

XIPH00282_m.1279_Xindex AKLFF--SASIASYGLV--SFINRNQQGELETFCKHLMSMAVQMGMRVATPFP---DSVQ

ALG1_WBGene00000105_Celegans YAVGV-----EQVE-----------------PMFKY-LKQNYSGIQLVVVVLPGK-----

ALG2_Cel_WBGene00000106_Celegans YASGV-----EQVE-----------------PMFKY-LKQTYSAIQLIVVVLPGK-----

HPO24_WBGene00011945_Celegans YAEPD-----QTV------------------KLLEH-LNEQY-NLQLVLCIVPGK-----

ALG3_WBGene000119108Celegans YGRSK-----EDVCTLFTEIA--------------DEYRVTNTVCDCIIVVLQSKN----

ALG4_WBGene00006449_Celegans YGRSK-----EDVCTLFTEIA--------------DEYRVTNTVCDCIIVVLQSKN----

RDE1_WBGene00004323_Celegans YDATKNEYAFYKNCTLNTGIGRFEIAATEAKNMFERLPDKEQKVLMFIIISKRQLN----

ZK218.8l_WBGene00013942_Celegans ------------------------------------------------------------

Mhap1s0264g09247_Mhapla ------------------------------------------------------------

Mhap1s0000g00097_Mhapla YAVGV-----DQVE-----------------PMFKY-LKQNYPGLQLVCVVLPGK-----

XIPH00220_m.1024_Xindex YAAGV-----DQVE-----------------PMFKY-LKQTFQGIQLIVVVLPGK-----

XIPH14718_m.27510_Xindex ------------------------------------------------------------

XIPH03015_m.8656_Xindex YHYDPV----EGAKPALVGLK--------------ASATRRSLKLDLVLVILPDGKGYEI

XIPH03061_m.8764_Xindex YAEGP-----DQVE-----------------PMFRF-LKGQYQTLQLILVILPGK-----

XIPH17950_m.31169_Xindex ------------------------------------------------------------

XIPH03137_m.8937_Xindex YHYDPV----EGAKPALVGLK--------------ASATRRSLKLDLVLVILPDGKGYEI

XIPH00171_m.824_Xindex YAAGV-----DQVE-----------------PMFKY-LKQTFQGIQLIVVVLPGK-----

XIPH13915_m.26514_Xindex ------------------------------------------------------------

XIPH01240_m.4301_Xindex ------------------------------------------------------------

XIPH28640_m.40226_Xindex ------------------------------------------------------------

XIPH00682_m.2641_Xindex YATGV-----DQVE-----------------PMFKY-LKQTFTGVQLIVVVLPGK-----

XIPH01763_m.5725_Xindex YAEGP-----DQVE-----------------PMFRF-LKGQYQTLQLILVILPGK-----

XIPH01058_m.3772_Xindex YAAGV-----DQVE-----------------PMFKY-LKQTFQGIQLIVVVLPGK-----

XIPH01422_m.4808_Xindex YAAGV-----DQVE-----------------PMFKY-LKQTFQGIQLIVVVLPGK-----

XIPH11895_m.23873_Xindex ------------------------------------------------------------

XIPH11139_m.22857_Xindex ------------------------------------------------------------

XIPH05696_m.14049_Xindex ------------------------------------------------------------

XIPH00358_m.1579_Xindex LTVPT-----NNIG-AIEESL----------------TRLKNMGAEFVIVTLPQKG----

XIPH00282_m.1279_Xindex YIRSA-----HEVKEAMTEIV--------------RDCGSKGKKCEFILVAMPTKN----

ALG1_WBGene00000105_Celegans TPVYAEVKRVGDTVLGIATQCVQAKNAI---------RTTPQTLSNLCLKMNVKLGGVNS

ALG2_Cel_WBGene00000106_Celegans TPIYAEVKRVGDTVLGIATQCVQAKNAI---------RTTPQTLSNLCLKMNVKLGGVNS

HPO24_WBGene00011945_Celegans SVVYGELKRKGE-LLGLTTQCVRSQNVS---------KASPHTLSNLCMKINSKLGGINV

ALG3_WBGene000119108Celegans SDIYMTVKEQSDIVHGIMSQCVLMKNVS---------RPTPATCANIILKLNMKMGGINS

ALG4_WBGene00006449_Celegans SDIYMTVKEQSDIVHGIMSQCVLMKNVS---------RPTPATCANIVLKLNMKMGGINS

RDE1_WBGene00004323_Celegans --AYGFVKHYCDHTIGVANQHITSETVTKALASLRHEKGSKRIFYQIALKINAKLGGINQ

ZK218.8l_WBGene00013942_Celegans ------------------------------------------------------------

Mhap1s0264g09247_Mhapla ------------------------------------------------------------

Mhap1s0000g00097_Mhapla TPVYAEVKRVGDTVLGIATQCVQAKNAT---------KTTPQTLSNLCLKMNVKLGGVNS

XIPH00220_m.1024_Xindex TPVYAEVKRVGDTVLGVATQCVQAKNVI---------RPNPQTLSNLCLKINVKLGGVNS

XIPH14718_m.27510_Xindex ------------------------------------------------------------

XIPH03015_m.8656_Xindex DHYYASVKSAGDVDVGLNTQCVKRGNVVG----RGGQGASPSLLANVCLKINAKLGGANN

XIPH03061_m.8764_Xindex TPVYAEVKRIGDTVLGVATQCVQTKNVN---------KPTTQTVSNLCLKINVKLGGVNC

XIPH17950_m.31169_Xindex ------------------------------------------------------------

XIPH03137_m.8937_Xindex DHYYASVKSAGDVDVGLNTQCVKRGNVVG----RGGQGASPSLLANVCLKINAKLGGANN

XIPH00171_m.824_Xindex TPVYAEVKRVGDTVLGVATQCVQAKNVI---------RPNPQTLSNLCLKINVKLGGVNS

XIPH13915_m.26514_Xindex ------------------------------------------------------------

XIPH01240_m.4301_Xindex -----------------------------------------QTLSNLCLKINVKLGGVNS

XIPH28640_m.40226_Xindex ------------------------------------------------------------

XIPH00682_m.2641_Xindex TPVYAEVKRVGDTVLGLATQCVQAKNVI---------RPNPQTLSNLCLKINVKLGGVNS

XIPH01763_m.5725_Xindex TPVYAEVKRIGDTVLGVATQCVQTKNVN---------KPTTQTVSNLCLKINVKLGGVNC

XIPH01058_m.3772_Xindex TPVYAEVKRVGDTVLGVATQCVQAKNVI---------RPNPQTLSNLCLKINVKLGGVNS

XIPH01422_m.4808_Xindex TPVYAEVKRVGDTVLGVATQCVQAKNVI---------RPNPQTLSNLCLKINVKLGGVNS

XIPH11895_m.23873_Xindex ------------------------------------------------------------

XIPH11139_m.22857_Xindex ------------------------------------------------------------

XIPH05696_m.14049_Xindex ------------------------------------------------------------

XIPH00358_m.1579_Xindex CPHYNEVKRVAEIVLGLMTQCVVKGNIMK----C-MQKRDRMTTGNIALKMNMKMGGVNS

XIPH00282_m.1279_Xindex TENYAAVKCAAEKNIGIITQVVIQRNIR---------QPKPQTCGNIVLKINAKLGGINW

ALG1_WBGene00000105_Celegans ILLPNVR---------PRIFNEPVIFFGCDITHPPAGDS---------RKPSIAAVVGSM

ALG2_Cel_WBGene00000106_Celegans ILLPNVR---------PRIFNEPVIFLGCDITHPAAGDT---------RKPSIAAVVGSM

HPO24_WBGene00011945_Celegans ILSS-PP---------QSLNSEPVLFIGCHLTRSSLASSSDSTSSIAHCDSSIACLVGSM

ALG3_WBGene000119108Celegans RIVADQIT-------NKYLVDQPTMVVGIDVTHPTQAEM-------RMNMPSVAAIVANV

ALG4_WBGene00006449_Celegans RIVADKIT-------NKYLVDQPTMVVGIDVTHPTQAEM-------RMNMPSVAAIVANV

RDE1_WBGene00004323_Celegans ELDWSEIAEISPEEKERRKTMPLTMYVGIDVTHPTSYSG---------IDYSIAAVVASI

ZK218.8l_WBGene00013942_Celegans ------------------------------------------------------------

Mhap1s0264g09247_Mhapla ------------------------------------------------------------

Mhap1s0000g00097_Mhapla ILLPAVR---------PRIFNEPIIFLGADITHPPAGDS---------RKPSIAGVVGSM

XIPH00220_m.1024_Xindex ILLPNVR---------PRVFNEPVIFIGADITHPPAGDS---------KKPSIAAVVGSM

XIPH14718_m.27510_Xindex ------------------------------------------------------------

XIPH03015_m.8656_Xindex TLLEDSRK-------EVKQFMKPILILGADVNHPAPTDS-------K-TAPSVAAVVGCL

XIPH03061_m.8764_Xindex ILLPTVR---------PKVFNEPVIFMGADMTHPPAGDN---------KKPSIAALVGSM

XIPH17950_m.31169_Xindex ------------------------------------------------------------

XIPH03137_m.8937_Xindex TLLEDSRK-------EVKQFMKPILILGADVNHPAPTDS-------K-TAPSVAAVVGCL

XIPH00171_m.824_Xindex ILLPNVR---------PRVFNEPVIFIGADITHPPAGDS---------KKPSIAAVVGSM

XIPH13915_m.26514_Xindex ------------------------------------------------------------

XIPH01240_m.4301_Xindex ILLPNIR---------PRIFNEPVIFIGADITHPPAGDS---------KKPSIAAVVGSM

XIPH28640_m.40226_Xindex ------------------------------------------------------------

XIPH00682_m.2641_Xindex ILLPNVR---------PRVFNEPVIFIGADITHPPAGDS---------KKPSIAAVVGSM

XIPH01763_m.5725_Xindex ILLPTVR---------PKVFNEPVIFMGADMTHPPAGDN---------KKPSIAALVGSM

XIPH01058_m.3772_Xindex ILLPNVR---------PRVFNEPVIFIGADITHPPAGDS---------KKPSIAAVVGSM

XIPH01422_m.4808_Xindex ILLPNVR---------PRVFNEPVIFIGADITHPPAGDS---------KKPSIAAVVGSM

XIPH11895_m.23873_Xindex ------------------------------------------------------------

XIPH11139_m.22857_Xindex ----DHSA-------CRTYIDDY----------LSVVYS---------RFPKTDPITEKV

XIPH05696_m.14049_Xindex ------------------------------------------------------------

XIPH00358_m.1579_Xindex KAQADDLA-------RRTLLEVPTLVLGIDVTHPSATEK---------KMPSISSVVGNV

XIPH00282_m.1279_Xindex KLTADEVA-------KKYLVNASTLVLGIDVTHPAPTDR---------RAPSIGAAVGNI

ALG1_WBGene00000105_Celegans DAHPSRYAATVRVQQ--------------HRQEIISDLTYMVRELLVQFYRNTRFKPARI

ALG2_Cel_WBGene00000106_Celegans DAHPSRYAATVRVQQ--------------HRQEIITDLTYMVRELLVQFYRNTRFKPARI

HPO24_WBGene00011945_Celegans DGHPTQFSPIFRTQP--------------RHQRTIVDMCEMTREAIINFRKSTGFKPHKI

ALG3_WBGene000119108Celegans DLLPQSYGANVKVQKKCRESVVYLL--DAIRERIIT------------FYRHTKQKPARI

ALG4_WBGene00006449_Celegans DLLPQSYGANVKVQKKCRESVVYLL--DAIRERIIT------------FYRHTKQKPARI

RDE1_WBGene00004323_Celegans NPGGTIYRNMIVTQEECRPGERAVAHGRERTD-ILE-AKFVKLLREFAENNDNRAPA-HI

ZK218.8l_WBGene00013942_Celegans ----------------------------------MD-KSHV----------SYKPPTTRL

Mhap1s0264g09247_Mhapla ------------------------------------------------------------

Mhap1s0000g00097_Mhapla DAHPSRYAATVRVQQ--------------HRHEIISELTFMVRELLIQFYRNTRFKPTRI

XIPH00220_m.1024_Xindex DAHPSRYAATVRIQQQ-------------QRQEIITDLANMVRELLIQFYRSTRFKPTRI

XIPH14718_m.27510_Xindex ------------------------------------------------------------

XIPH03015_m.8656_Xindex DRELATYAVRIMVQQRRVEFITDLK--TAVHQML------------DMYLARNRALPERI

XIPH03061_m.8764_Xindex DAHPSRYGASVRIQYPRRVNDERTGKQKDERSERIEELALMVRELLIQFYQSTRFKPTRI

XIPH17950_m.31169_Xindex ------------------------------------------------------------

XIPH03137_m.8937_Xindex DRELATYAVRIMVQQRRVEFITDLK--TAVHQML------------DMYLARNRALPERI

XIPH00171_m.824_Xindex DAHPSRYAATVRIQQQ-------------QRQEIITDLANMVRELLIQFYRSTRFKPTRI

XIPH13915_m.26514_Xindex ------------------------------------------------------------

XIPH01240_m.4301_Xindex DAHPSRYAATVRIQQ--------------HRHEIITDLANMIRELLIQFYRSTRFKPTRI

XIPH28640_m.40226_Xindex ------------------------------------------------------------

XIPH00682_m.2641_Xindex DAHPSRYAATVRIQQQ-------------QRQEIITDLANMVRELLIQFYRSTRFKPTRI

XIPH01763_m.5725_Xindex DAHPSRYGASVRIQYPRRVNDERTGKQKDERSERIEELALMVRELLIQFYQSTRFKPTRI

XIPH01058_m.3772_Xindex DAHPSRYAATVRIQQQ-------------QRQEIITDLANMVRELLIQFYRSTRFKPTRI

XIPH01422_m.4808_Xindex DAHPSRYAATVRIQQQ-------------QRQEIITDLANMVRELLIQFYRSTRFKPTRI

XIPH11895_m.23873_Xindex ------------------------------------------------------------

XIPH11139_m.22857_Xindex KYAWMRKGRSV-------------------------------------------------

XIPH05696_m.14049_Xindex ------------------------------------------------------------

XIPH00358_m.1579_Xindex DDSFMRFGASVRIQKHRRESIVAGHLDQLVRDRLVEYHKYVSQEN-AKNKESKKSQPERV

XIPH00282_m.1279_Xindex DTLPAKFGVSVTVQKHRHEAVVYMT--DAIQKRVTE------------FYQQTQRKPERI

ALG1_WBGene00000105_Celegans VVYRDGVSEGQFFNVLQYELRAIREACMMLER-----------GYQPGITFIA-------

ALG2_Cel_WBGene00000106_Celegans VVYRDGVSEGQLFNVLQYELRAIREACVMLES-----------GYQPGITFIA-------

HPO24_WBGene00011945_Celegans IIYRAGIADVTVDEIMQTELRAVRDACAMIEY-----------GFQPGITFIG-------

ALG3_WBGene000119108Celegans IVYRDGVSEGQFSEVLREEIQSIRTACLAIAE----------DFR-PPITYIV-------

ALG4_WBGene00006449_Celegans IVYRDGVSEGQFSEVLREEIQSIRTACLAIAE----------DFR-PPITYIV-------

RDE1_WBGene00004323_Celegans VVYRDGVSDSEMLRVSH----D-ELRSLKSEV-KQFMSERDGEDPEPKYTFIV-------

ZK218.8l_WBGene00013942_Celegans GLYPFVSATGCVLVVDTIKIHDILLKTVNFEKGLHYVNAGTIEYMASEYNYTI-------

Mhap1s0264g09247_Mhapla ------------------------------------------------------------

Mhap1s0000g00097_Mhapla IVYRDGVSEGQFLNVLQSELRSMREACMMLER-----------GYQPGITFIA-------

XIPH00220_m.1024_Xindex IMYRDGVSEGQFFNVLQHELRALREACLMLEH-----------GYQPGITFIA-------

XIPH14718_m.27510_Xindex ----------------------------------------------PKITLIV-------

XIPH03015_m.8656_Xindex VMFRDGVGESMFLKVLTNELRSIREACASIFEYAKSRKLTTQTVYKPPITFIV-------

XIPH03061_m.8764_Xindex VLYRKGVSEGQFYQVLQHELRSMREACMMLES-----------GYQPGITYIT-------

XIPH17950_m.31169_Xindex ------------------------------------------------------------

XIPH03137_m.8937_Xindex VMFRDGVGESMFLKVLTNELRSIREACASIFEYAKSRKLTTQTVYKPPITFIV-------

XIPH00171_m.824_Xindex IMYRDGVSEGQFFNVLQHELRALREACLMLEH-----------GYQPGITFIA-------

XIPH13915_m.26514_Xindex ------------------------------------------------------------

XIPH01240_m.4301_Xindex IMYRDGVSEGQFFNVLQHELRALREACMMLER-----------AYQPGITFIA-------

XIPH28640_m.40226_Xindex ------------------------------------------------------------

XIPH00682_m.2641_Xindex IMYRDGVSEGQFFNVLQHELRALREACLMLEH-----------GYQPGITFIA-------

XIPH01763_m.5725_Xindex VLYRKGVSEGQFYQVLQHELRSMREACMMLES-----------GYQPGITYIT-------

XIPH01058_m.3772_Xindex IMYRDGVSEGQFFNVLQHELRALREACLMLEH-----------GYQPGITFIA-------

XIPH01422_m.4808_Xindex IMYRDGVSEGQFFNVLQHELRALREACLMLEH-----------GYQPGITFIA-------

XIPH11895_m.23873_Xindex ------------------------------------------------------------

XIPH11139_m.22857_Xindex ----------------------VMSDCVFRRV----------VCWPPTRKALSSASAVTI

XIPH05696_m.14049_Xindex ------------------------------------------------------------

XIPH00358_m.1579_Xindex IIYRDGVAEGQFQEVLREELGGVREACRALRA----------DYR-PKLTLII-------

XIPH00282_m.1279_Xindex IVYRDGVGETQFEQVMTTEVNNIMNACTVIGG----------PDYRPKITFIV-------

ALG1_WBGene00000105_Celegans ---VQKRHHTRLFAVDKKD------------------------------QVGKAYNIPPG

ALG2_Cel_WBGene00000106_Celegans ---VQKRHHTRLFAADKAD------------------------------QVGKAFNIPPG

HPO24_WBGene00011945_Celegans ---LDVTHHTRLFAANEKD------------------------------RVGNSQNVPAG

ALG3_WBGene000119108Celegans ---VQKRHHARIFCKFPND------------------------------MVGKAKNVPPG

ALG4_WBGene00006449_Celegans ---VQKRHHARIFCKYQND------------------------------MVGKAKNVPPG

RDE1_WBGene00004323_Celegans ---IQKRHNTRLLRRMEKDKPVVNKDLTPAETDVAVAAVKQWEEDMKESKETGIVNPSSG

ZK218.8l_WBGene00013942_Celegans ---KIKNHFVY--------------SL--------APELSKKNPKVKQSETPRFINPPSG

Mhap1s0264g09247_Mhapla ------------------------------------------------------------

Mhap1s0000g00097_Mhapla ---LQKRHHTRLFAVDKKD------------------------------QVGKAFNIPPG

XIPH00220_m.1024_Xindex ---VQKRHHTRLFAIDKKD------------------------------QVGKAYNIPPG

XIPH14718_m.27510_Xindex ---AQKRHHTRFFPMSQQA------------------------------ACGKAMNVRPG

XIPH03015_m.8656_Xindex ---VQKRHHTRLFCVNEQD------------------------------RVGGGNNVPPG

XIPH03061_m.8764_Xindex ---VQKRHHTRLFCAERKD------------------------------MDGKSGNIPAG

XIPH17950_m.31169_Xindex -----------------------------------------------------RNCCHRI

XIPH03137_m.8937_Xindex ---VQKRHHTRLFCVNEQD------------------------------RVGGGNNVPPG

XIPH00171_m.824_Xindex ---VQKRHHTRLFAIDKKD------------------------------QVGKAYNIPPG

XIPH13915_m.26514_Xindex ------------------------------------------------------------

XIPH01240_m.4301_Xindex ---VQKRHHTRLFAVDKKD------------------------------QVGKAFNIPPG

XIPH28640_m.40226_Xindex ------------------------------------------------------------

XIPH00682_m.2641_Xindex ---VQKRHHTRLFAIDKKD------------------------------QVGKAYNIPPG

XIPH01763_m.5725_Xindex ---VQKRHHTRLFCAERKD------------------------------MDGKSGNIPAG

XIPH01058_m.3772_Xindex ---VQKRHHTRLFAIDKKD------------------------------QVGKAYNIPPG

XIPH01422_m.4808_Xindex ---VQKRHHTRLFAIDKKD------------------------------QVGKAYNIPPG

XIPH11895_m.23873_Xindex --------------MSQQA------------------------------ACGKAMNVRPG

XIPH11139_m.22857_Xindex CPVPVSDSSTNTYR--SEE------------------------------RFSRRNCCHRI

XIPH05696_m.14049_Xindex --------------MSQQA------------------------------ACGKAMNVRPG

XIPH00358_m.1579_Xindex ---AQKRHHTRFFPTVQGQ------------------------------GSGKADNIWPG

XIPH00282_m.1279_Xindex ---VQKRHHTRFFCCNQQD------------------------------MCGKGKNIPPG

ALG1_WBGene00000105_Celegans TTVDVGITHPTEFDFYLCSHAGIQGTSRPSHYHVLWDDNNLTADELQQLTYQMCHTYVRC

ALG2_Cel_WBGene00000106_Celegans TTVDVGITHPTEFDFFLCSHAGIQGTSRPSHYHVLWDDNDLTADELQQLTYQMCHTYVRC

HPO24_WBGene00011945_Celegans TLVETGITVNNLFEFYLVSHAGIQGTSRPTKYVVMWDDNSIPSADIHEMTYQLCHTQSRC

ALG3_WBGene000119108Celegans TTVDTGIVSPEGFDFYLCSHYGVQGTSRPARYHVLLDECKFTADEIQNITYGMCHTYGRC

ALG4_WBGene00006449_Celegans TTVDTGIVSPEGFDFYLCSHYGVQGTSRPARYHVLLDECKFTADEIQSITYGMCHTYGRC

RDE1_WBGene00004323_Celegans TTVDKLIVSKYKFDFFLASHHGVLGTSRPGHYTVMYDDKGMSQDEVYKMTYGLAFLSARC

ZK218.8l_WBGene00013942_Celegans TVVDKLVVSGYKFDFYLNSHHAVLGTSRPAHYTVMYDDMGMSQDEVYKMTDALAFLSARC

Mhap1s0264g09247_Mhapla ------------------------------------------------------------

Mhap1s0000g00097_Mhapla TTVDVGITHPTEFDFYLCSHAGIQGTSRPSHYHVLWDDNNLSADELQQLTYQLCHTYVRC

XIPH00220_m.1024_Xindex TTVDVGITHPTEFDFFLCSHAGIQGTSRPSHYHVLWDDNNMSADELQQLTYQLCHTYVRC

XIPH14718_m.27510_Xindex TVVDGGITNVEQFEFYPCSHFGIQGTSRPIRYHVLYDDNNFSADHIQLMTYYMCHLYARC

XIPH03015_m.8656_Xindex TVVDTEIVHPAEFDFYLCSHLGLQGTSRPTHYHVLWDDSDFSADELQMLTYYLCHTYIRA

XIPH03061_m.8764_Xindex TTVDSGITHPQEFDFYLCSHAGIQGTSRPSHYHVLWDDNNFSSDELQQLTYQLCHTYVRC

XIPH17950_m.31169_Xindex TVVDGGITHPEQFEFYLCSHFGIQGTSRPIRYHVLYDDNNFSADHIQLMTYYMCHLSVFP

XIPH03137_m.8937_Xindex TVVDTEIVHPAEFDFYLCSHKGLQGTSRPGHYQVLWDDSSFTADQLQILTYYLCYTYARA

XIPH00171_m.824_Xindex TTVDVGITHPTEFDFFLCSHAGIQGTSRPSHYHVLWDDNNMSADELQQLTYQLCHTYVRC

XIPH13915_m.26514_Xindex ------------------------------------------------------------

XIPH01240_m.4301_Xindex TAVDVGITHPTEFDFFLCSHAGIQGTSRPSHYHVLWDDNNLSADELQQLTYQLCHTYVRC

XIPH28640_m.40226_Xindex ------------------------------------------------------------

XIPH00682_m.2641_Xindex TTVDVGITHPTEFDFFLCSHAGIQGTSRPSHYHVLWDDNNMSADELQQLTYQLCHTYVRC

XIPH01763_m.5725_Xindex TTVDSGITHPQEFDFYLCSHAGIQGTSRPSHYHVLWDDNNFSSDELQQLTYQLCHTYVRC

XIPH01058_m.3772_Xindex TTVDVGITHPTEFDFFLCSHAGIQGTSRPSHYHVLWDDNNMSADELQQLTYQLCHTYVRC

XIPH01422_m.4808_Xindex TTVDVGITHPTEFDFFLCSHAGIQGTSRPSHYHVLWDDNNMSADELQQLTYQLCHTYVRC

XIPH11895_m.23873_Xindex TVVDGGITNVEQFEFYPCSHFGIQGTSRPIRYHMLYNH---------------SHLYARR

XIPH11139_m.22857_Xindex TVVDGGITHPEQFEFYLCSHFGIQGTSRPIRYHVLYDDNNFSADHIQLMTYYMCHLYARC

XIPH05696_m.14049_Xindex TVVDGGITNVEQFEFYPCSHFGIQGTSRPIRYHMLYNH---------------SHLYARR

XIPH00358_m.1579_Xindex TVIDRDITHPEQFEFYMCSHHGIQGTSRPIRYHVLHDDNNFSADHIQAMTFYLCHVYARC

XIPH00282_m.1279_Xindex TVVDNTVTHPTQFDFFLCSHFGIQGTSRPAHYHVLKDDNQFSPDELQSITYYLCHMYARC

ALG1_WBGene00000105_Celegans TRSVSIPAPAYYAHLVAF------------RARYHLVDREHDSGE-GSQ-----------

ALG2_Cel_WBGene00000106_Celegans TRSVSIPAPAYYAHLVAF------------RARYHLVDRDHGSGEEGSQ-----------

HPO24_WBGene00011945_Celegans TRSVSIPSPVYYAKLVAQ------------RAKILMADENFDMERFRLC-----------

ALG3_WBGene000119108Celegans TRSVSIPTPVYYADLVAT------------RARCHIKRKLGLADNNDCDTNSLSSSLASL

ALG4_WBGene00006449_Celegans TRSVSIPTPVYYADLVAT------------RARCHVKRKLGLADNNDCDTNSRSSTLASL

RDE1_WBGene00004323_Celegans RKPISLPVPVHYAHLSCEKAKELYRTY-----KEHY---IGDYAQPRT-----RHEMEHF

ZK218.8l_WBGene00013942_Celegans RKPISLPAPVHFYVYKLFQC------W-----P---------------------------

Mhap1s0264g09247_Mhapla ------------------------------------------------------------

Mhap1s0000g00097_Mhapla TRSVSVPAPAYYAHLVAF------------RARYHLVDREHDSGE-GSQ-----------

XIPH00220_m.1024_Xindex TRSVSIPSPAYYAHLVAF------------RARYHLVDREHDSGE-GSQ-----------

XIPH14718_m.27510_Xindex TRTVSIPAPIYYADLACR------------RARAHLYTQITDFSSDTLS-----------

XIPH03015_m.8656_Xindex MKSVSIPPPCYYADLVAY------------RARQYLTWKQSGSETAS-------------

XIPH03061_m.8764_Xindex TRSVSIPAPAYYAQWVAF------------RARYHLVDKDHDSGEGSAH-----------

XIPH17950_m.31169_Xindex RQFITPIWPAVAPVLICTIKYRISAAIQFRNGQAQLVKRLEYRRTN*-------------

XIPH03137_m.8937_Xindex TKAVSVPPPIYYADLAAF------------RARKYLAWKTQDLEEGS-------------

XIPH00171_m.824_Xindex TRSVSIPSPAYYAHLVAF------------RARYHLVDREHDSGE-GSQ-----------

XIPH13915_m.26514_Xindex ------------------------------------------------------------

XIPH01240_m.4301_Xindex TRSVSIPAPAYYAHLVAF------------RARYHLVDREHDSGE-GSQ-----------

XIPH28640_m.40226_Xindex ------------------------------------------------------------

XIPH00682_m.2641_Xindex TRSVSIPSPAYYAHLVAF------------RARYHLVDREHDSGE-GSQ-----------

XIPH01763_m.5725_Xindex TRSVSIPAPAYYAQWVAF------------RARYHLVDKDHDSGEGSAH-----------

XIPH01058_m.3772_Xindex TRSVSIPSPAYYAHLVAF------------RARYHLVDREHDSGE-GSQ-----------

XIPH01422_m.4808_Xindex TRSVSIPSPAYYAHLVAF------------RARYHLVDREHDSGE-GSQ-----------

XIPH11895_m.23873_Xindex TRTVCIPAPIYYADLIGFSSDRFRI-----------------------------------

XIPH11139_m.22857_Xindex TRTVSIPAPIYYADLACR------------RARAHLYDKISDFSSDTIS-----------

XIPH05696_m.14049_Xindex TRTVCIPAPIYYADLIGFSSDRFRI-----------------------------------

XIPH00358_m.1579_Xindex TRAVSIPAPIYYADLACR------------RARGHIFDKVSDFSSDTMS-----------

XIPH00282_m.1279_Xindex TRSVSIPAPTYYAHLACA------------RARHHLREVIGDANSDTFS-----------

ALG1_WBGene00000105_Celegans ---PSGT-SED-----------------TTLSNMARAVQVHPDAN-NVMYFA--------

ALG2_Cel_WBGene00000106_Celegans ---PSGTSSED-----------------TTLSSMAKAVQVHPDSN-NVMYFA--------

HPO24_WBGene00011945_Celegans ---GIGRNDGM------------------SFT----------------------------

ALG3_WBGene000119108Celegans LNVRTGSGKGKKSHAPSVDDESYSLPDAASDQILQDCVSVAADFK-SRMYFI--------

ALG4_WBGene00006449_Celegans LNVRTGSGKGKKSYAPSVDDESYSLSDATSDQILQDCVSVATDFK-SRMYFI--------

RDE1_WBGene00004323_Celegans LQT---------------------------------------NVKYPGMSFA--------

ZK218.8l_WBGene00013942_Celegans ------------------------------------------------------------

Mhap1s0264g09247_Mhapla ------------------------------------------------------------

Mhap1s0000g00097_Mhapla ---PSGT-SED-----------------TTLSNMARAVQVHPDAN-STMLGHGHNT----

XIPH00220_m.1024_Xindex ---PSGT-SED-----------------TTMSSMARAIQVHPDAN-NVMYFA*-------

XIPH14718_m.27510_Xindex --TSSTSGADTSST-------------KVSLEEMIRGSAVDNEMK-LTMYFV*-------

XIPH03015_m.8656_Xindex --S--GSSGS-----------------VISLAELQQFVSVKDNISERGMYFV*-------

XIPH03061_m.8764_Xindex ---SSGNSEDR-----------------SSTHSVARAVPVHSDTS-NVMYFACPRS----

XIPH17950_m.31169_Xindex ------------------------------------------------------------

XIPH03137_m.8937_Xindex --VATGTSGGSDSLP------------PELILQLQSYVQVKDDIVAKGMFFV*-------

XIPH00171_m.824_Xindex ---PSGT-SED-----------------TTMSSMARAIQVHPDAN-NVMYFA*-------

XIPH13915_m.26514_Xindex ------------------------------------------------------------

XIPH01240_m.4301_Xindex ---PSGT-SED-----------------TTMSSMARAVQVHPDAN-NVMYFA*-------

XIPH28640_m.40226_Xindex ------------------------------------------------------------

XIPH00682_m.2641_Xindex ---PSGT-SED-----------------TTMSSMARAIQVHPDAN-NVMYFA*-------

XIPH01763_m.5725_Xindex ---SSGNSEDR-----------------SSTHSVARAVPVHSDTS-NVMYFA*-------

XIPH01058_m.3772_Xindex ---PSGT-SED-----------------TTMSSMARAIQVHPDAN-NVMYFA*-------

XIPH01422_m.4808_Xindex ---PSGT-SED-----------------TTMSSMARAIQVHPDAN-NVMYFA*-------

XIPH11895_m.23873_Xindex ----------------------------GCIGIRIESSTVADGLR-LQNVFVVNVLNFYC

XIPH11139_m.22857_Xindex --QRSGTAGETS---------------GISEDELIKSSTVADGLR-FQMYFS*-------

XIPH05696_m.14049_Xindex ----------------------------GCIGIRIESSTVADGLR-LQNVFVVNVLNFYC

XIPH00358_m.1579_Xindex --QRSGSGGETE-T-------------AVSEDELIRSSTVHDALH-MKMYFT*-------

XIPH00282_m.1279_Xindex --DAGS---GDSGT-------------SSSEEDIKKAVDIVDSLR-TKMYFV*-------

ALG1_WBGene00000105_Celegans --------------

ALG2_Cel_WBGene00000106_Celegans --------------

HPO24_WBGene00011945_Celegans --------------

ALG3_WBGene000119108Celegans --------------

ALG4_WBGene00006449_Celegans --------------

RDE1_WBGene00004323_Celegans --------------

ZK218.8l_WBGene00013942_Celegans --------------

Mhap1s0264g09247_Mhapla --------------

Mhap1s0000g00097_Mhapla ---RDTVCLEFL--

XIPH00220_m.1024_Xindex --------------

XIPH14718_m.27510_Xindex --------------

XIPH03015_m.8656_Xindex --------------

XIPH03061_m.8764_Xindex ---EER--------

XIPH17950_m.31169_Xindex --------------

XIPH03137_m.8937_Xindex --------------

XIPH00171_m.824_Xindex --------------

XIPH13915_m.26514_Xindex --------------

XIPH01240_m.4301_Xindex --------------

XIPH28640_m.40226_Xindex --------------

XIPH00682_m.2641_Xindex --------------

XIPH01763_m.5725_Xindex --------------

XIPH01058_m.3772_Xindex --------------

XIPH01422_m.4808_Xindex --------------

XIPH11895_m.23873_Xindex YIVRELVMFIFTI*

XIPH11139_m.22857_Xindex --------------

XIPH05696_m.14049_Xindex YIVRELVMFIFTI*

XIPH00358_m.1579_Xindex --------------

XIPH00282_m.1279_Xindex --------------

PIWI

Cel_WBGene00004178 MASGSGRGRGRGSGSNNSGGKDQKYLGTIQPDLFIRQQGQSKTGSSGQPQKCFANFIPIE 60

XIPH00931_m.3408 ------------------------------------------------------------ 0

Cel_WBGene00004178 MTQSDYSIYQYHVEFEPTVDSKANREKMLRDNNVTDEIGHHFVFDGMILYLKEEWEQNQM 120

XIPH00931_m.3408 ------------------------------------------------------------ 0

Cel_WBGene00004178 IEVQHPIDRSLICIRFKQTNRFLVDDPQTINIFNTIIRRSFDALQLTQLGRNYFNWGDSR 180

XIPH00931_m.3408 ------------------------------------------------------------ 0

Cel_WBGene00004178 AVPDYNMSILPGYETAIRMYEENFMLCVENRFKMVREESMYILFHKELRSCQNNPQRVQE 240

XIPH00931_m.3408 ------------------------------------------------------------ 0

Cel_WBGene00004178 KMNEMYGGTTIITRYNNKLHRYTRLDYSISPLSEFVKDGQSITLKEYFKNQYGIEITVDD 300

XIPH00931_m.3408 ------------------------------------------------------------ 0

Cel_WBGene00004178 QPIIISEGKPKQPGEPPQVSYIVPELCFPTGLTDEMRKDFKMMKEIAKHTRMSPQQRLVE 360

XIPH00931_m.3408 ------------------------------------------MQDLANFMRVPPHRRIEN 18

*:::*:. *: *::*: :

Cel_WBGene00004178 SRKLIVDLSKNEKVMECFKYWGISLGQDLANVQARVLKSEPLQGK---KTYEGKQAEWAR 417

XIPH00931_m.3408 ARIHARNVQQNDDVMALFKHWGLTVGPQLLELDARVLKGEELQCGNRIITYSEDTADWSR 78

:* ::.:*:.** **:**:::* :* :::*****.* ** **. . *:*:*

Cel_WBGene00004178 GVKECGIYRGSNMTNWIVIGPGSGNSGLLSQKFIEEARRLGKILQVQLGEPMCVPIRGIS 477

XIPH00931_m.3408 EQRGAPLNEPVALDEWILIFTEKDRDS--AEQFYKTLCKVGPPMGMDIQEPNIIQLPTDR 136

: . : . : :**:* ..... :::* : ::* : ::: ** : :

Cel_WBGene00004178 PNDYLEGVKGAIKQVDGEDIHMLVVMLADDNKTRYDSLKKFLCVECPIPNQCVNLRTLAG 537

XIPH00931_m.3408 VNDFVDAIRYAVSQFPQT--RIVVPVVPNENKIRYDAIKKLCCVEMPVPSQVVNTMK--- 191

**:::.:: *:.*. :::* :: ::** ***::**: *** *:*.* ** .

Cel_WBGene00004178 KSKDGGENKNLGSIVLKIVLQMICKTGGALWKVNIPLKNTMIVGYDLYHDSTLKGKTVGA 597

XIPH00931_m.3408 ---SLKNRKNLMTIVTKIAMQINVKMDGTLWNVQIPMKNIMVVGYDVYHDTQQKGKSVGA 248

. :.*** :** **.:*: * .*:**:*:**:** *:****:***: ***:***

Cel_WBGene00004178 CVSTTSNDFTQFYSQTRPHENPTQLGNNLTHFVRKALKQYYDSNDQTLPSRLILYRDGAG 657

XIPH00931_m.3408 CLFSTDPLATKYFTQCALHSDHLEQVENLASFLRSGIKKYHE-RNRVLPERIIFYRDGVA 307

*: :*. *::::* *.: : :**: *:*..:*:*:: .::.**.*:*:****..

Cel_WBGene00004178 DGQIPYIKNTEVKLVRDACDAVTDKAAELSNKVQEKIKLAFIIVTKRVNMRILKQGSSLD 717

XIPH00931_m.3408 DGQLPAVYHYELPEVLEALRYFG---------HDYKPKMTFVVISKRMPTRFFEKLP-GG 357

***:* : : *: * :* . : * *::*::::**: *:::: .

Cel_WBGene00004178 NAINPQPGTVVDTTVTRPERMDFYLVPQFVNQGTVTPVSYNIIHDDTDLGPDKHQQLAFK 777

XIPH00931_m.3408 ESDNPPPGTVVDSVITRADRYDFFLVPQCVRQGTVTPVCYNVIYDNSGLKPEHLQRFTFK 417

:: ** ******:.:** :* **:**** *.*******.**:*:*::.* *:: *:::**

Cel_WBGene00004178 LCHLYYNWQGTVRVPAPCQYAHKLAFLTAQSLHDDANGCLRDKLFFL- 824

XIPH00931_m.3408 LCHLYYNWQGTVRVPGPCQYAHKLAYLVGQSLHRPPDPAMCDRLFFL* 464

***************.*********:*..**** : .: *:****

ERI/DICER

ERI1_WBGene00001332_Celegans MSADEPSPEDEKYLESLRDLLKISQEFDASNAKQNDEPEKTAVEVESAETRTDESEKSID

Mhap1s0171g07555_Mhapla ------------------------------------------------------------

XIPH19513_Xindex ------------------------------------------------------------

ERI1_WBGene00001332_Celegans IPREQQLLPSERVEPLKSMVEPEYVKKVIRQMDTMTAEQLKQALMKIKVSTGG-NKKTLR

Mhap1s0171g07555_Mhapla -------MINERVEYLFEF------------FESMSDIELQREMEIIDEDSSELKRSKII

XIPH19513_Xindex ------------------------------------------------------------

ERI1_WBGene00001332_Celegans KRVAQYYRKENALLNRKMEPNADKTARFFDYLIAIDFECTCVEIIYDYPHEIIELPAVLI

Mhap1s0171g07555_Mhapla ATLRDHYRDALIEL-----RKKYSTRKFYNYFIVIDFECSCEENNYDFEHEIIEFPAVMI

XIPH19513_Xindex -----------------------------------------------------------M

:

ERI1_WBGene00001332_Celegans DVREMKIISEFRTYVRPVRNPKLSEFCMQFTKIAQETVDAAPYFREALQRLYTWMRKFNL

Mhap1s0171g07555_Mhapla SVESCN---------------------------IQEDVDKAPTFPIALQLFRGWMAKHGL

XIPH19513_Xindex NTATWTIESEFHRYVRPVAHPQLSTFCTGLTGIVQDMVDNGATLDVTLKQFDEWLKSEGL

.. . *: ** . : :*: : *: . .*

ERI1_WBGene00001332_Celegans ----G-QKNSRFAFVTDGPHDMWKFMQFQCLLSNIRMPHMFRSFINIKKTFKEKFNGL--

Mhap1s0171g07555_Mhapla DGRRNMGKARRFCYITDGPWDIGKFFQMECFRSNQSIPHDFRCFMNIRRSFVNFYTMQSQ

XIPH19513_Xindex LSTDG-TSTKEWTLVTCGDWDLKIMLKNQCDHFGYRRPDYFHRWINVKRPFSY-------

. . .: :* * *: ::: :* . *. *: ::*::: *

ERI1_WBGene00001332_Celegans -------------IK------GNGKSG--IENMLERLDLSFVGNKHSGLDDATNIAAIAI

Mhap1s0171g07555_Mhapla PPRYHCDGNSVHVVEGTTTKEKLFPNGISLNIMLKHLNIQFTGREHCGMDDTLNIAFIVI

XIPH19513_Xindex -------------LT------GAFPKG--MMQMLEMLNLAHQGRHHSGIDDARNIAEVVR

: .* : **: *:: . *..*.*:**: *** :.

ERI1_WBGene00001332_Celegans QMMKLKIELRINQKCSYKENQRSAA---------RKDEEREL------EDAANVDLTSVD

Mhap1s0171g07555_Mhapla KLLEEGAELRQ--KLVFNEKTKAPSCVASTSSSIENDDKKEKNPKNILKMPNEEDKSNGN

XIPH19513_Xindex VLGIRGTSFEL---TSFNKT*---------------------------------------

: .:. :::.

ERI1_WBGene00001332_Celegans ISRRDFQLWMRRLPLKLSSVTRREFINEEYLDCDSCDDLTDDKVKHLHSCDIYEIFDEKT

Mhap1s0171g07555_Mhapla EKIFVWKKWWDEMPYKLVRITRYEFLGDRHLECESCDEGDN-------------------

XIPH19513_Xindex ------------------------------------------------------------

ERI1_WBGene00001332_Celegans SASFTDSKCLIC

Mhap1s0171g07555_Mhapla ------------

XIPH19513_Xindex ------------

DCR1_WBGene00000939_Celegans ------------------------------------------------------------

EFV48890_Tspiralis ------------------------------------------------------------

EFV57735_Tspiralis MGMAASSTNGDAMEDESEMEAFDVVRIADDVIQRLRFGSQDIFKSEDPVIDDMLIESAYR

EFV48479_Tspiralis ------------------------------------------------------------

EFV58865_Tspiralis ------------------------------------------------------------

Mhap1s0258g09126_Mhapla ------------------------------------------------------------

Mhap1s0258g09127_Mhapla ------------------------------------------------------------

XIPH14444_m.27161_Xindex ------------------------------------------------------------

XIPH15429_m.28360_Xindex ------------------------------------------------------------

XIPH19060_m.32298_Xindex ------------------------------------------------------------

DCR1_WBGene00000939_Celegans ------------------------------------------------------------

EFV48890_Tspiralis ------------------------------------------------------------

EFV57735_Tspiralis IQYETLRQDLIATQEEVARLKRQLQERDYAERKKGRSGTVEVGTKIEKHCSRRENQKLSN

EFV48479_Tspiralis ------------------------------------------------------------

EFV58865_Tspiralis ------------------------------------------------------------

Mhap1s0258g09126_Mhapla ------------------------------------------------------------

Mhap1s0258g09127_Mhapla ------------------------------------------------------------

XIPH14444_m.27161_Xindex ------------------------------------------------------------

XIPH15429_m.28360_Xindex ------------------------------------------------------------

XIPH19060_m.32298_Xindex ------------------------------------------------------------

DCR1_WBGene00000939_Celegans ------------------------------------------------------------

EFV48890_Tspiralis ------------------------------------------------------------

EFV57735_Tspiralis ELVKLLLILEFMFTVQYSCLSNSATFRKNNIDMVYGTLNNLVRASKDCVTELERHVSQVL

EFV48479_Tspiralis ------------------------------------------------------------

EFV58865_Tspiralis ------------------------------------------------------------

Mhap1s0258g09126_Mhapla ------------------------------------------------------------

Mhap1s0258g09127_Mhapla ------------------------------------------------------------

XIPH14444_m.27161_Xindex ------------------------------------------------------------

XIPH15429_m.28360_Xindex ------------------------------------------------------------

XIPH19060_m.32298_Xindex ------------------------------------------------------------

DCR1_WBGene00000939_Celegans ------------------------------------------------------------

EFV48890_Tspiralis ------------------------------------------------------------

EFV57735_Tspiralis YDLKHSQDLQYYVKEDLTFVSAKEIDVGNRAVIIVFVPFPQYKQYRRILVRLIHEVEKKF

EFV48479_Tspiralis ------------------------------------------------------------

EFV58865_Tspiralis ------------------------------------------------------------

Mhap1s0258g09126_Mhapla ------------------------------------------------------------

Mhap1s0258g09127_Mhapla ------------------------------------------------------------

XIPH14444_m.27161_Xindex ------------------------------------------------------------

XIPH15429_m.28360_Xindex ------------------------------------------------------------

XIPH19060_m.32298_Xindex ------------------------------------------------------------

DCR1_WBGene00000939_Celegans ------------------------------------------------------------

EFV48890_Tspiralis ------------------------------------------------------------

EFV57735_Tspiralis GGKHVVFVAKRRILPKPTRGKRKITQKQKRPRSRTLAAVHDEYLNDIVFPAEIVGKRIRV

EFV48479_Tspiralis ------------------------------------------------------------

EFV58865_Tspiralis ------------------------------------------------------------

Mhap1s0258g09126_Mhapla ------------------------------------------------------------

Mhap1s0258g09127_Mhapla ------------------------------------------------------------

XIPH14444_m.27161_Xindex ------------------------------------------------------------

XIPH15429_m.28360_Xindex ------------------------------------------------------------

XIPH19060_m.32298_Xindex ------------------------------------------------------------

DCR1_WBGene00000939_Celegans ------------------------MVR-VRADLQCFNPRDYQVELLDKATKKNTIVQLGT

EFV48890_Tspiralis ------------------------------------------------------------

EFV57735_Tspiralis RLDGSRLLKVHLDRNQKAAVEHKVNRMEKEQSGRLENVRLYQKELLECAKKENVIVTLGT

EFV48479_Tspiralis ------------------------------------------------------------

EFV58865_Tspiralis ------------------------MNRLYDFETDFFTPRDHLTRLVDIGRRCNLIAPLGS

Mhap1s0258g09126_Mhapla ------------------------------------------------------------

Mhap1s0258g09127_Mhapla ------------------------MSPPKDFSGKCIPPRDYQVELLDRAKIQNTIISLGT

XIPH14444_m.27161_Xindex ------------------------------------------------------------

XIPH15429_m.28360_Xindex -----------------------MAFRQQEIETENFTPRPYQLELLEKAKKENIIIPLAT

XIPH19060_m.32298_Xindex ------------------------------------------------------------

DCR1_WBGene00000939_Celegans GSGKTFIAVLLLKEYGVQLFAPLDQGG------KRAFF-----VVEKVNLVEQQAIHIEV

EFV48890_Tspiralis ------------------------------------------------------------

EFV57735_Tspiralis GTGKTFIAVMLIREMSESVHQPLKEGG------KRSLF-----IVDKVPLVKQQAEHIRI

EFV48479_Tspiralis ------------------------------------------------------------

EFV58865_Tspiralis AVDRLYVSVMLIREFSSMLRKPCSKNR------RWCVY-----LVDRATSIKSVADRIRI

Mhap1s0258g09126_Mhapla ------------------------------------------------------------

Mhap1s0258g09127_Mhapla GAGKTFVAVLLIKEYAQRLLHRNEKAAFLVNTGYFLTFLIFNNHVNLVELVAQQAEHIEF

XIPH14444_m.27161_Xindex ------------------------------------------------------------

XIPH15429_m.28360_Xindex GSGKTFVAVMLIREYAAETRIPWEQGA------KRSFF-----LVNQVALVQQQAEQIRT

XIPH19060_m.32298_Xindex ------------------------------------------------------------

DCR1_WBGene00000939_Celegans HTSFKVGQVHGQTSSGLWDSKEQCDQFMKR-HHVVVITAQCLLDLIRHAYLKIEDMCVLI

EFV48890_Tspiralis ------------------------------------------------------------

EFV57735_Tspiralis NTNLKVGEFHGALGVDCWSAKIWMEFFDK--FHVLVMTAEIFRNILTHGFIKFDIVNLIV

EFV48479_Tspiralis ------------------------------------------------------------

EFV58865_Tspiralis YTNLNVGEIFCDIHL-ESEAKQYCAAQMEG-NEIFVTTGTHFMKLLDLSFIPRNCYCLVI

Mhap1s0258g09126_Mhapla ------------------------------------------------------------

Mhap1s0258g09127_Mhapla HSSLSVARISGSTIKRKHERGEVEKITRNNQASIVLLKA-----SLFPCFIGDSNYCTSV

XIPH14444_m.27161_Xindex ------------------------------------------------------------

XIPH15429_m.28360_Xindex HTDLKVSEYHSGTNCDRWTSEQWEMELKE--NQVLVMTAQIFLNMITHRFVNMQRANVIV

XIPH19060_m.32298_Xindex ------------------------------------------------------------

DCR1_WBGene00000939_Celegans FDECHHALGSQHPYRSIMVDYKLLKKD---KPVPRVLGLTASLIKAKVAPEKLMEQLKKL

EFV48890_Tspiralis ------------------------------------------------------------

EFV57735_Tspiralis FDECHHATK-QHPYKKIMELYKLCYNNNDSGIQPRILGLTASVMNKKGDEIGLQKAIRNL

EFV48479_Tspiralis ------------------------------------------------------------

EFV58865_Tspiralis FEDCHLAIR-SHPYRNIVKEFLNIEK----EYRPRLLGLTMSLINDEVKGDCLQYGIDTM

Mhap1s0258g09126_Mhapla ------------------------------------------------------------

Mhap1s0258g09127_Mhapla S-----------------------------------------------------------

XIPH14444_m.27161_Xindex ------------------------------------------------------------

XIPH15429_m.28360_Xindex FDECHHATK-RHPYREIMREYENCKD-----QKPRVLGLTASIINEKVTPSKLKRLVYKL

XIPH19060_m.32298_Xindex ------------------------------------------------------------

DCR1_WBGene00000939_Celegans ESAMDSVIETASDLVSLSKYGAKPYEVVIICKDFEIGCLGIPNFDTVIEIFDETVAFVNT

EFV48890_Tspiralis ------------------------------------------------------------

EFV57735_Tspiralis ETTLCSRVVGTSHAELLKLYSADPHIAIVACKNDN--KYDWHELDLFFQHLVRSTQKSLE

EFV48479_Tspiralis ------------------------------------------------------------

EFV58865_Tspiralis QEVLCSKVVL-TMLSPAKKFLKRDTKVVLISYP-STSLFSWDMFDNYILHLLVSPTFILE

Mhap1s0258g09126_Mhapla ---MDSHIETTSNYTQICKYVTKPKQFIICTKDD---CT---NEKFVVDLLERLRSFIEK

Mhap1s0258g09127_Mhapla ------------------------------------------------------------

XIPH14444_m.27161_Xindex ------------------------------------------------------------

XIPH15429_m.28360_Xindex ECALSSKIETTSDMTGPSKYSTKPTTFMLKCRD*--------------------------

XIPH19060_m.32298_Xindex ------------------------------------------------------------

DCR1_WBGene00000939_Celegans TTEFHPD-LDLDPRRPIKDSLKTTRAVFRQLGPWAAWRTAQVWEKELGKII-KSQVL--P

EFV48890_Tspiralis ------------------------------------------MLQSTKNLATRISTRLND

EFV57735_Tspiralis RCGRECEGNVSLYIQHVCDVLAKLDSIMGQLGPWCALQVCKQMLQSTTNLATRISTLLAD

EFV48479_Tspiralis ------------------------------------------------------------

EFV58865_Tspiralis NITLPND-DDMICMQHVIESLNKVKWIHDEMGPWCAWKVCQKQEMQLNRMK-RIKP---G

Mhap1s0258g09126_Mhapla NEDFHSE-LEVDPRRPIFEINP-----------LNTYDK---------------------

Mhap1s0258g09127_Mhapla ------------------------------------------------------------

XIPH14444_m.27161_Xindex ------------------------------------------------------------

XIPH15429_m.28360_Xindex ------------------------------------------------------------

XIPH19060_m.32298_Xindex ------------------------------------------------------------

DCR1_WBGene00000939_Celegans DK---------------TLRFLNMAKTSMITIKRLLEPEMKKIKSIE-ALRPYV-----P

EFV48890_Tspiralis DEDNNLFSLFTSGSDASSSVGLS-VETCLKVTSVKLKRHIGLVDSVQKLKCVFIRQQFVD

EFV57735_Tspiralis DEDNNLFSLFTSGSDASSSVWLEVLKTCLKVTSVKLKRHIGLVDSVQKLKCVFIRQQFVD

EFV48479_Tspiralis ------------------------------------------------------------

EFV58865_Tspiralis TM---------------QYLLIEMGQTYLRCLRKVFENHVKNLKNFS-SLCAYI-----T

Mhap1s0258g09126_Mhapla --------------------------------------------LAN-PKKSYL-----S

Mhap1s0258g09127_Mhapla ------------------------------------------------------------

XIPH14444_m.27161_Xindex ------------------------------------------------------------

XIPH15429_m.28360_Xindex ------------------------------------------------------------

XIPH19060_m.32298_Xindex ------------------------------------------------------------

DCR1_WBGene00000939_Celegans QRVIRLFEILETFNPEFQKERMKLEKAEHLS--AIIFVDQRYIAYSLLLMMRHI-KSWEP

EFV48890_Tspiralis QQQQAVWNCIRQSTPGSLLYSGELNFQLEKNNSRKFENTNQANGPPFQILIDTAKKLQPR

EFV57735_Tspiralis QQQQAVWNCIRQSTPGSLLYSGELNFQLEKNNSRKFENTNGANGPPFQILIDTAKKLQPR

EFV48479_Tspiralis ------------------------------------------------------------

EFV58865_Tspiralis GHVYSFIELLSHNKMRS-VFADDENFPENFC--CIVFFKHRYIAYVYKILLKTLQNLWPD

Mhap1s0258g09126_Mhapla DRMRKLVEILKSYAPSK---REKSGIKDTLF--GLIFVKERFIAFMINNLLRFLVKQNPE

Mhap1s0258g09127_Mhapla ------------------------------------------------------------

XIPH14444_m.27161_Xindex ------------------------------------------------------------

XIPH15429_m.28360_Xindex ------------------------------------------------------------

XIPH19060_m.32298_Xindex ------------------------------------------------------------

DCR1_WBGene00000939_Celegans KFKFVNPDYVVGASGRNLASSDSQ-GLHKRQTEVLRRFHRNEINCLIATSVLEEGVDVKQ

EFV48890_Tspiralis SYSYLEVDYVVGSRMSAEV----AEPSFARQEEVLKNFRHGKLNLLAATSILEEGIDVRH

EFV57735_Tspiralis SYSYLEVDYVVGSRMSAEV----AEPSFARQEEVLKNFRHGKLNLLAATSILEEGIDVRH

EFV48479_Tspiralis ------------------------------------------------------------

EFV58865_Tspiralis MFGYLKVDFLVGYDSET-ADASKE-ALHERQHEVLKKFRTKELNLLLTTRVLAKGIELRG

Mhap1s0258g09126_Mhapla DFGHLKVDFLVGQTGNSETGDEDRRLAARKQEQTLCRFRNGQLNLLVTTNVLEEGIDLRN

Mhap1s0258g09127_Mhapla ------------------------------------------------------------

XIPH14444_m.27161_Xindex ------------------------------------------------------------

XIPH15429_m.28360_Xindex ------------------------------------------------------------

XIPH19060_m.32298_Xindex ------------------------------------------------------------

DCR1_WBGene00000939_Celegans CNLVIKFDRPLDMRSYVQSKGRARRAGSRYVITVEEKDTAACDSDLKDFQQIEKILLSRH

EFV48890_Tspiralis CNYVIRFDTPLTFRSFVQSKGRARQKIAYYTILVQDRFLKSFQEMLNSFVETEKFLKVNG

EFV57735_Tspiralis CNYVIRFDTPLTFRSFVQSKGRARQKIAYYTILVQDRFLESFQEMLNSFVETEKFLKVNG

EFV48479_Tspiralis ------------------------------------------------------------

EFV58865_Tspiralis ANCVIHYDEPESLRSFIYVKNRANKPNSHYFILLSECTSALSASVINTFVDIDKIIRNYS

Mhap1s0258g09126_Mhapla CNLVIRFDPPMDFRSFVQSSGRARKENSAFYMLIEEKNYLEFMMDLTGYAQAEELVLRRY

Mhap1s0258g09127_Mhapla ------------------------------------------------------------

XIPH14444_m.27161_Xindex ------------------------------------------------------------

XIPH15429_m.28360_Xindex ------------------------------------------------------------

XIPH19060_m.32298_Xindex ------------------------------------------------------------

DCR1_WBGene00000939_Celegans RTVNNPI--------------E----DDSDRFEEFDVDSQMEPYVV--------------

EFV48890_Tspiralis RDLNILTRTKDRDRSYSGNNDQVDNCRKEVNLPNGDVDSLVEPYYTYFE---------EN

EFV57735_Tspiralis RDLNILTRTKDRDRSYSGNNDQVDNCRKEVNLPNGDVDSLVEPYYTYFE---------EN

EFV48479_Tspiralis ------------------------------------------------------------

EFV58865_Tspiralis LLD---Y--------------E----DDNL-ELPDDLDDTFKPYYPKKSEEVGDDDEEAD

Mhap1s0258g09126_Mhapla RSGNDFT--------LNGKEEG----NNETKIIQPHLDDVVAPYVVIT------------

Mhap1s0258g09127_Mhapla ------------------------------------------------------------

XIPH14444_m.27161_Xindex ------------------------------------------------------------

XIPH15429_m.28360_Xindex ------------------------------------------------------------

XIPH19060_m.32298_Xindex ------------------------------------------------------------

DCR1_WBGene00000939_Celegans --EKTGATLKMSTAIALINRYCSKLPSDIFTRLVPHNQIIPI---EENGVTKYCAELLLP

EFV48890_Tspiralis GLVKKAACLVLSSAHNVIKRYCNKLRKDRFADSSPKFSVQTILNK--DLSISYVATVQLP

EFV57735_Tspiralis GLVKKAACLVLSSAHNVIKRYCNKLRKDRFADSSPKFSVQTILNK--DLSISYVATVQLP

EFV48479_Tspiralis ------------------------------------------------------------

EFV58865_Tspiralis KLKNNNVCATLANSIRIVNHYCQRLPCDIFSRLVADCHVEKVMSGECAPSPKYRATLKLP

Mhap1s0258g09126_Mhapla --PNGTAKVSLSGAIHLVNRYCSKLPSDIFTRLVPRYTIKTI---SENGQTLYIAELYLP

Mhap1s0258g09127_Mhapla ------------------------------------------------------------

XIPH14444_m.27161_Xindex ------------------------------------------------------------

XIPH15429_m.28360_Xindex ------------------------------------------------------------

XIPH19060_m.32298_Xindex ------------------------------------------------------------

DCR1_WBGene00000939_Celegans INSPIKHAIVLKNPMPNKKTAQMAVALEACRQLHLEGELDDNLLPKGRESIAKLLEHIDE

EFV48890_Tspiralis TTSPLKKKI-EGKPMQNAKLAEMAAAFETAKMLHAMGELNEFLIPPVTVRKE---IELKE

EFV57735_Tspiralis TTSPLKKKI-EGKPMQNAKLAEMAAAFETAKMLHAMGELNEFLIPPVTVRKE---IELKE

EFV48479_Tspiralis ------------------------------------------------------------

EFV58865_Tspiralis INSPGPTVD-------SVMLARRSVALETVKLLHRRGELYDSLMPIGKEMVASLLIA-DE

Mhap1s0258g09126_Mhapla INSPIKEPI-TSKPMASKRLSLMTAALEACKRLHQRKELNDQLLPAGKEIVLDLLGEV--

Mhap1s0258g09127_Mhapla ------------------------------------------------------------

XIPH14444_m.27161_Xindex ------------------------------------------------------------

XIPH15429_m.28360_Xindex ------------------------------------------------------------

XIPH19060_m.32298_Xindex ------------------------------------------------------------

DCR1_WBGene00000939_Celegans EPDEYAPGIAAKVGSSKRKQLYDKKIARALNESFVEADKECFIYAFELERFREAE-----

EFV48890_Tspiralis DEQ---------FFSISSRSYYLKEVPDALYKAIPRADQKSYLYAISISSI---------

EFV57735_Tspiralis DEQ---------FFSISSRSYYLKEVPDALYKAIPRADQKSYLYAISISSI---------

EFV48479_Tspiralis ------------------------------------------------------------

EFV58865_Tspiralis DDEEWPVTGKACPGSSKRRQYYNKAVRAGACKSLIECLKRAIPV-FDEPGYFHAIIIREV

Mhap1s0258g09126_Mhapla DDDEYLPYLPSKMGSSKKKRLYDRKMSKTLSSTLPAQESECILYVMEMKLIKPV-----S

Mhap1s0258g09127_Mhapla ------------------------------------------------------------

XIPH14444_m.27161_Xindex ------------------------------------------------------------

XIPH15429_m.28360_Xindex ------------------------------------------------------------

XIPH19060_m.32298_Xindex ------------------------------------------------------------

DCR1_WBGene00000939_Celegans LTLNPKRRKFEDPFNYEYCFGFLSAKEIPKIPPFPVFLRQGNMKVRLIVAPKK-TTVTAA

EFV48890_Tspiralis -------------ESFLPRLGILVSKPIGNLPGFSVFTDESYIDVEIEFVEET--SYSAH

EFV57735_Tspiralis -------------ESFLPRLGILVSKPIGNLPGFSVFTDESYIDVEIEFVEET--SYSAH

EFV48479_Tspiralis ------------------------------------------------------------

EFV58865_Tspiralis SEICNGEANISQPEVGKRILGIFSSKPIPQIPAFMIYEKTKSFLVEIRPCAST-ITVNKG

Mhap1s0258g09126_Mhapla EERNPKRRKIIDPFESNSAFGFLSSKELPKVPGFPVFQRNGEMIVQIRKAKNQPVRLTFE

Mhap1s0258g09127_Mhapla ------------------------------------------------------------

XIPH14444_m.27161_Xindex ------------------------------------------------------------

XIPH15429_m.28360_Xindex ------------------------------------------------------------

XIPH19060_m.32298_Xindex ------------------------------------------------------------

DCR1_WBGene00000939_Celegans QLQEIQLFHNYLFTQVLQMCKTGNLEFDGTSNAPLNTLIVPLNKRK-DDMSYTINMKYVS

EFV48890_Tspiralis QLDILTSFHCCIFKNYLFREEEDFI-FDPE-NAACSYLIVPLKCEYNSKC--SVDLSTAE

EFV57735_Tspiralis QLDILTSFHCCIFKNYLFREEEDFI-FDPE-NAACSYLIVPLKCEYNSKC--SVDLSTAE

EFV48479_Tspiralis ------------------------------------------------------------

EFV58865_Tspiralis QMAVIAEFNMYIFSEILKLEKYSMK-YQPE-EAENAFFIVP--SIS-TETDYILDWQFIV

Mhap1s0258g09126_Mhapla LFQLICLFHQHIFEDILRIARGGVV-FAPG-HSPIPLLIVPLKKLGTVDLDYEIDRDYLN

Mhap1s0258g09127_Mhapla ------------------------------------------------------------

XIPH14444_m.27161_Xindex ------------------------------------------------------------

XIPH15429_m.28360_Xindex ------------------------------------------------------------

XIPH19060_m.32298_Xindex ------------------------------------------------------------

DCR1_WBGene00000939_Celegans EVVANMENMPRIPKDEVRRQYK-------------FNAEDYKDAIVMPWYRNLEQPVF-Y

EFV48890_Tspiralis KIINWSKDVSHIPQQR-SEKFI-------------MNSSSYFNAVVYPWYKNKNDRDFYY

EFV57735_Tspiralis KIINWSKDVSHIPQQR-SEKFI-------------MNSSSYFNAVVYPWYKNKNDRDFYY

EFV48479_Tspiralis ------------------------------------------------------------

EFV58865_Tspiralis EVVNYWDRVPRRPDEEQRKNFVFDISNLVKIGFVFFNAFRYKDAVVMPWYRSQEMSHC-Y

Mhap1s0258g09126_Mhapla W---DIREPPTTPSDEIRKQYV-------------FEESSYLNAVVSPWYRSEDQSAF-Y

Mhap1s0258g09127_Mhapla ------------------------------------------------------------

XIPH14444_m.27161_Xindex ------------------------------------------------------------

XIPH15429_m.28360_Xindex ------------------------------------------------------------

XIPH19060_m.32298_Xindex ------------------------------------------------------------

DCR1_WBGene00000939_Celegans YVAEILPEWRPSSKFPDTHFETFNEYFIKKYKLEIYDQNQSLLDVDFTST-RLNLLQPRI

EFV48890_Tspiralis FVTAVDNTCSPMSPFPLKMYRSFAEYFEVTKQVKVLDKNQPLISVKM-------------

EFV57735_Tspiralis FVTAVDNTCTPMSPFPLKMYRSFAEYFEVTKQVKVLDKNQPLLSVKMVSFKRLNLLCEKP

EFV48479_Tspiralis ------------------------------------------------------------

EFV58865_Tspiralis FVLNVDEGLTPLTKFPDDEYESFKSYFWQKYGLEIYHDDQPLLNVDYTAS-RLNRLFPRR

Mhap1s0258g09126_Mhapla YVAEIMTDQFPSSSFPDEKFTCFNQYFMSKYQLEIYNQKQNLLDVDHTSA-RMNLLLPRA

Mhap1s0258g09127_Mhapla ------------------------------------------------------------

XIPH14444_m.27161_Xindex ------------------------------------------------------------

XIPH15429_m.28360_Xindex ------------------------------------------------------------

XIPH19060_m.32298_Xindex ------------------------------------------------------------

DCR1_WBGene00000939_Celegans QNQPRRSRTVSNSSTSNIPQASASDSKESNTSVPHSSQRQILVPELMDIHPISATLWNVI

EFV48890_Tspiralis ------------------------------------------------------------

EFV57735_Tspiralis ISILL----------------------FDFD-DDANSCHCKLVPELVIIHPMPASMWRCL

EFV48479_Tspiralis ------------------------------------------------------------

EFV58865_Tspiralis QARRESLE-------------------ADYDTMSVVAQKQKLVPELVDIHPIPASTWRCL

Mhap1s0258g09126_Mhapla ITGKSALRS------------------------LDPSQRQILVPELLHIHPLSATLWSII

Mhap1s0258g09127_Mhapla ------------------------------------------------------------

XIPH14444_m.27161_Xindex ------------------------------------------------------------

XIPH15429_m.28360_Xindex ------------------------------------------------------------

XIPH19060_m.32298_Xindex ------------------------------------------------------------

DCR1_WBGene00000939_Celegans AALPSIFYRVNQLLLTDELRETILVKAFGKEKTKLDDNVEWNSLAYATEYEEK-----Q-

EFV48890_Tspiralis ------------------------------------------------------------

EFV57735_Tspiralis IFLPTVLYRMNHLLIAEQLRLQILREAMFPGE-VLEENCEIQPLNKDWYLLASRLNDMCI

EFV48479_Tspiralis ------------------------------------------------------------

EFV58865_Tspiralis QYLPSILYRLSSLLLANELRLQVLLDEYNNDPTP--DGYCWAPIDKAWLKETMRMTDHGC

Mhap1s0258g09126_Mhapla VTLPTILYRLNSLLLADEFRSKVLEDALKLGS-QTPSDFEWTPLQYVTPNDDQNQ-----

Mhap1s0258g09127_Mhapla ------------------------------------------------------------

XIPH14444_m.27161_Xindex ------------------------------------------------------------

XIPH15429_m.28360_Xindex ------------------------------------------------------------

XIPH19060_m.32298_Xindex ------------------------------------------------------------

DCR1_WBGene00000939_Celegans --TIIVKKIQQLRDLN--QKSIEDQE---------------RETRENDKIDDGEELFNIG

EFV48890_Tspiralis ------------------------------------------------------------

EFV57735_Tspiralis VDNQLPVK---LSKLSVNSNSA---------------------------AA----ATATG

EFV48479_Tspiralis ------------------------------------------------------------

EFV58865_Tspiralis PWAKMVNKSSKNSGINCSSNSIDSGKNKNNNNNNNSNNLSNSNIKSEDSARFEQMDFEIS

Mhap1s0258g09126_Mhapla ---KSIRNLDQLRKINQQEKE-----------NEVAMECDTVEEKGNETAASGINDFEIG

Mhap1s0258g09127_Mhapla ------------------------------------------------------------

XIPH14444_m.27161_Xindex ------------------------------------------------------------

XIPH15429_m.28360_Xindex ------------------------------------------------------------

XIPH19060_m.32298_Xindex ------------------------------------------------------------

DCR1_WBGene00000939_Celegans VWDPEEAV-----RIG---------------VEISSRDDRMDGEDQDT------------

EFV48890_Tspiralis ------------------------------------------------------------

EFV57735_Tspiralis SIDPETQALLEEFDVRFCNACP--------TVPVDDQLDILTGNSVA-------------

EFV48479_Tspiralis ------------------------------------------------------------

EFV58865_Tspiralis VWEPVQEF-----VFN--------------GFTDDGKHDCLDANSADFGSDQLPMSDGQQ

Mhap1s0258g09126_Mhapla VWDPTPNI-----DNILDEPPPVIHNAPVNGL-IPGRRNGLRGVIAARDEE-------LS

Mhap1s0258g09127_Mhapla ------------------------------------------------------------

XIPH14444_m.27161_Xindex ------------------------------------------------------------

XIPH15429_m.28360_Xindex ------------------------------------------------------------

XIPH19060_m.32298_Xindex ------------------------------------------------------------

DCR1_WBGene00000939_Celegans ----VGLTQGL-HDGNISDEDDELPFVMHDYTARLTSNR--------NGIGAWSGSESIV

EFV48890_Tspiralis ------------------------------------------------------------

EFV57735_Tspiralis -------------ESHTAESNDECLFDTH-FSLQLLSSI---------------------

EFV48479_Tspiralis ------------------------------------------------------------

EFV58865_Tspiralis SFEDCLMQMGD-YDGSMLDSDEEIYFDT-EFSRKFMNVMSLHGSYGQHPFGLL-FGQPIE

Mhap1s0258g09126_Mhapla EIIAVGNDTTIHNYGDISDDDD-VAAEYDKFKF------LMHNKMTTSDIGEL-GEMDVR

Mhap1s0258g09127_Mhapla ------------------------------------------------------------

XIPH14444_m.27161_Xindex ------------------------------------------------------------

XIPH15429_m.28360_Xindex ------------------------------------------------------------

XIPH19060_m.32298_Xindex ------------------------------------------------------------

DCR1_WBGene00000939_Celegans PSGWGDWDGPEPDNS----PMPFQILGGPGGLNVQALMADVGRVFDPSTASSSLSQTVQE

EFV48890_Tspiralis ------------------------------------------------------------

EFV57735_Tspiralis ------------------------------------------------------Q-----

EFV48479_Tspiralis ------------------------------------------------------------

EFV58865_Tspiralis PSGWEVDDVPTMPAADEHCDVGLHFVSAGQSLNVSTLLADVRDADEKVRGLQQQQPNAKE

Mhap1s0258g09126_Mhapla PAGWNDDSNVNVIQI-ENESLPLTISTNNPHINIASLMNDLEKNYAAFGTSTSTANNNKV

Mhap1s0258g09127_Mhapla ------------------------------------------------------------

XIPH14444_m.27161_Xindex ------------------------------------------------------------

XIPH15429_m.28360_Xindex ------------------------------------------------------------

XIPH19060_m.32298_Xindex ------------------------------------------------------------

DCR1_WBGene00000939_Celegans STVSPPKQLTKEEEQFKKLQNDLLKQAKERLEALEMSEDMEKPRRLEDTVNLEDYGDDQE

EFV48890_Tspiralis ------------------------------------------------------------

EFV57735_Tspiralis -----PQQQPLEKVDF---------------------------------GIVESDGES--

EFV48479_Tspiralis ------------------------------------------------------------

EFV58865_Tspiralis EDTVPTSNAT------------------------------------AQKVPVDRNGTEAK

Mhap1s0258g09126_Mhapla NTPVTPSATTTDKTVIQRK--------ELNLDSLN---------------VIDQNDPSIK

Mhap1s0258g09127_Mhapla ------------------------------------------------------------

XIPH14444_m.27161_Xindex ------------------------------------------------------------

XIPH15429_m.28360_Xindex ------------------------------------------------------------

XIPH19060_m.32298_Xindex ------------------------------------------------------------

DCR1_WBGene00000939_Celegans NQEDENTPTNFPKTIDEEIEELSIGARKKQEIDDNAAKTDVLERENCEVLP------V--

EFV48890_Tspiralis ------------------------------------------------------------

EFV57735_Tspiralis ---------DFFVAAPALPE-----------------------S----------EL----

EFV48479_Tspiralis ------------------------------------------------------------

EFV58865_Tspiralis -----ITSRHKIQAVDTLAE--------EETVDLNMFKRAVYNRKGNVLMNGNESWKNFT

Mhap1s0258g09126_Mhapla RQNEDILPEEVFWGMDEMEENIN-----------NEGTSKIIQTKSADSISPTRDLRD--

Mhap1s0258g09127_Mhapla ------------------------------------------------------------

XIPH14444_m.27161_Xindex ------------------------------------------------------------

XIPH15429_m.28360_Xindex ------------------------------------------------------------

XIPH19060_m.32298_Xindex ------------------------------------------------------------

DCR1_WBGene00000939_Celegans ---AINEKSRSFSFEKESKAINGRLIRQRSEEYVSHIDSDIGLGVSPCLLLTALTTSNAA

EFV48890_Tspiralis ------------------------------------------------------------

EFV57735_Tspiralis ---------NLFVEPSDV-------------SDTATAPAQHRPGPQCRDVLRALTLRKAQ

EFV48479_Tspiralis ------------------------------------------------------------

EFV58865_Tspiralis NEQMLTLDSSWYEWPGEFR-TAALLPAGETCTFLEIANEETPFGPNPREVLHAITASAVA

Mhap1s0258g09126_Mhapla ------LEKQKF-------EITAPELNWMPFSFMWNLVDQNPYGVSPALLLQALTTSSAA

Mhap1s0258g09127_Mhapla ------------------------------------------------------------

XIPH14444_m.27161_Xindex ------------------------------------------------------------

XIPH15429_m.28360_Xindex ------------------------------------------------------------

XIPH19060_m.32298_Xindex ------------------------------------------------------------

DCR1_WBGene00000939_Celegans DGMSLERFETIGDSFLKFATTDYLYHTLLDQHEGKLSFARSKEVSNCNLYRLGKKLGIPQ

EFV48890_Tspiralis ------------------------------------------------------------

EFV57735_Tspiralis DMFDLESMEALGDSFLKFIVSLHVFIKETNWNEGRLTSLRSEIVSNTNLFNLGKQKLLQA

EFV48479_Tspiralis ------------------------------------------------------------

EFV58865_Tspiralis ETFNLEGLEILGDSFLKYVTTVYCYKAYSKMHEGKLSLLRSRMISNYNLYKLGKRKNIPQ

Mhap1s0258g09126_Mhapla DGINLERLETIGDSFLKMAVTNYFYHKHTEQHEGKLSFARSKEVSNSHLFYLGRQRGIPL

Mhap1s0258g09127_Mhapla ------------------------------------------------------------

XIPH14444_m.27161_Xindex ------------------------------------PIFRSKLVCNYNLYKLGKRKGLAE

XIPH15429_m.28360_Xindex ------------------------------------------------------------

XIPH19060_m.32298_Xindex ------------------------------------------------------------

DCR1_WBGene00000939_Celegans LIVANKFDAHDSWLPPCYIPTC-DFKAPNTDDAEEK-------DNEIERILDG-QVI---

EFV48890_Tspiralis ------------------------------------------------------------

EFV57735_Tspiralis KLTAVPFDPTAQWLPPCFRSLAALESG-----YESVNE-----------LIDEGD-----

EFV48479_Tspiralis ------------------------------------------------------------

EFV58865_Tspiralis YMIAIKFDPSDTWLPPCYVPLNCEVQDTAIE-EEDK-------LME-QRLMNDESVV---

Mhap1s0258g09126_Mhapla LIETLKFDPHVNWLPPCYASTS-EFHAVNPFDYTDLDEEQDQREVPMEGVETNKTVDQQQ

Mhap1s0258g09127_Mhapla ------------------------------------------------------------

XIPH14444_m.27161_Xindex MMVASAFEPKDNWIPPGYRPLERNGENVGVE-EEDK-------SMEM--ALEEET-----

XIPH15429_m.28360_Xindex ------------------------------------------------------------

XIPH19060_m.32298_Xindex ------------------------------------------------------------

DCR1_WBGene00000939_Celegans -EEKPENKTGWDIGGD--VSKSTTDGIETITFPKQARVGNDDISP----LPYNLLTQQHI

EFV48890_Tspiralis ------------------------------------------------------------

EFV57735_Tspiralis -SKKKNE------------------AL-------KANTPTPVVVAERYEMCSLNRTHQVI

EFV48479_Tspiralis ------------------------------------------------------------

EFV58865_Tspiralis -EQQRNV----ETTGR-----QATTGSK-----STKWVPEDLSQL----VPFNLLAQQGI

Mhap1s0258g09126_Mhapla KNNKETIATGWGTLDDDRQNYKCENGVETLTFPQQTKSEIPDLPP----MPYNMLTQQWI

Mhap1s0258g09127_Mhapla ------------------------------------------------------------

XIPH14444_m.27161_Xindex -----------GACG-------------------QEEQVEQMASQ----ISYSMTTQQYI

XIPH15429_m.28360_Xindex ------------------------------------------------------------

XIPH19060_m.32298_Xindex ------------------------------------------------------------

DCR1_WBGene00000939_Celegans SDKSIADAVEALIGVHLLTLGPNPTLKVMNWMGLKVIQKDQKSDVP-------SPL--LR

EFV48890_Tspiralis ------------------------------------------------------------

EFV57735_Tspiralis YDKSIADCVEALVGCYLLEAGMRPAIKLLKWFGIDIDGNLMNLLSSSSSS---SSSSSTA

EFV48479_Tspiralis ---------------------MRPAIKLLKWFGIDIDGNLMNLLSSSSSSSSSSSSSSTA

EFV58865_Tspiralis SDKGVADCVEALIGAYLLFCGTRRTLDFLHWLGLKVEDEVSLRGFSRCIV-SPTPISFAN

Mhap1s0258g09126_Mhapla SDKSIADAVEALIGAHLIQLGQSSTLKFMNWLGIKVLTDISS-----------LPSPLLR

Mhap1s0258g09127_Mhapla ------------------------------------------------------------

XIPH14444_m.27161_Xindex ADKSIADTVEAMIGVYLLTYGPEKTTRFLQWFGLNPTADDDVWSKQ-------ARSPLLR

XIPH15429_m.28360_Xindex ------------------------------------------------------------

XIPH19060_m.32298_Xindex ------------------------------------------------------------

DCR1_WBGene00000939_Celegans FI-------DTPTNPNASLNFLNNLWQQFQFTQLEEKIGYRFKE-------RAYLVQAFT

EFV48890_Tspiralis ------------------------------------------------------------

EFV57735_Tspiralis FC-------SENCVLIGPEAKIHSIWTAYDLNSFEAKIGYRFTN-------KAYLIQALT

EFV48479_Tspiralis FC-------SENCVLIGPEAKIHSIWTAYDLNSFEAKIGYRFTN-------KAYLIQALT

EFV58865_Tspiralis FDLYGVTKSALINNSADSEACLKTLWNRFSLSQFEDIIGYRFKD-------RSLLVQALT

Mhap1s0258g09126_Mhapla FI-------DTPEDPNLSLKHLALFYEKFDFATVENNIGYKFANKVVFIRGGAYLVQAFT

Mhap1s0258g09127_Mhapla ------------------------------------------------------------

XIPH14444_m.27161_Xindex YV-------------QDPEAHLEQLWQRYNLTSFESQIGYEFKD-------KAFLVQAFT

XIPH15429_m.28360_Xindex ------------------------------------------------------------

XIPH19060_m.32298_Xindex ------------------------------------------------------------

DCR1_WBGene00000939_Celegans HASYIN--NRVTGCYQRLEFLGDAVLDYMITRYLFEDSRQYSPGVLTDLRSALVNNTIFA

EFV48890_Tspiralis ------------------------------------------------------------

EFV57735_Tspiralis HSSYNEVETPVTDSYERLEFLGDAILDYLISRHLYSSKRIRSPGLLSDLRAALVNN----

EFV48479_Tspiralis HSLYNEVETPVTDSYERLEFLGDAILDYLISRHLYSSKRIRSPGLLSDLRAALVNN----

EFV58865_Tspiralis HSTYFY--NEVTDCYQRLEFLGDAVLDHLITRHLYEDKRMHSPGMLTDLRSAL-------

Mhap1s0258g09126_Mhapla HASYYN--NRVTGCYQRLEFLGDAVLDYMITRFLYEHKRQYSPGVLTDLRSALVNNTIFA

Mhap1s0258g09127_Mhapla ------------------------------------------------------------

XIPH14444_m.27161_Xindex HASYNY--NRVTDCYQRLEFLGDAVLDYMITQTLFQYSQHHSPGVLTDLRSALVNNTIFA

XIPH15429_m.28360_Xindex ------------------------------------------------------------

XIPH19060_m.32298_Xindex ------------------------------------------------------------

DCR1_WBGene00000939_Celegans SLAVKFEFQKHFIAMCPGLYHMIEKFVKLCSERNF--DTNFNAEMYM----VTTEEEIDE

EFV48890_Tspiralis ------------------------------------------------------------

EFV57735_Tspiralis ----------YFLYFNAELLSVNERFVLAMRGLKE--SVNFHNELYMMEEEQDDDEKETS

EFV48479_Tspiralis ----------YFLYFNAELLSVNERFVLAMRGLKE--SVNFHNELYMMEEEQDDDEKETS

EFV58865_Tspiralis ----------YFMYLCPGLMMMIEKFVKTLNIIKE--NANFDREVRVHLLNLYLLEGEEE

Mhap1s0258g09126_Mhapla SLAVKYSFHKHFVMICPPLYQMVEKFVNFCKQKDFLHCANFDDEIFMLT--EEEIDEEDL

Mhap1s0258g09127_Mhapla ------------------------------------------------------------

XIPH14444_m.27161_Xindex SLAVKYNFHKHFMALSPVINTITTKFVGL-------------------------------

XIPH15429_m.28360_Xindex ------------------------------------------------------------

XIPH19060_m.32298_Xindex -----------------------------IEERRQ--ESNFD-------EELYLLNEEDC

DCR1_WBGene00000939_Celegans GQEEDIEVPKAMGDIFESVAGAIYLDSGRNLDTTWQVIFHMMRGTI----------ELCC

EFV48890_Tspiralis ------------------------------------------------------------

EFV57735_Tspiralis EFAEHVEVPKPLGDIFESVAGAIFLDSHCSLATVWQVYYNMIAEEIGKQRSDFTSFNKCL

EFV48479_Tspiralis EFAEHVEVPKPLGDIFESVAGAIFLDSHCSLATVWQVYYNMIAEEIGKQRSDFTSFNKCL

EFV58865_Tspiralis NAEEQVEVPKALGDIFESVAGAIYLDSGCSLHTVWCIYYNMLREEI----------EKCC

Mhap1s0258g09126_Mhapla VSEEDVEVPKAMGDIFESIAGAVYLDCGMDLDIVWRVFYNLMRDVI----------QKCC

Mhap1s0258g09127_Mhapla ------------------------------------------------------------

XIPH14444_m.27161_Xindex ------------------------------------------------------------

XIPH15429_m.28360_Xindex ------------------------------------------------------------

XIPH19060_m.32298_Xindex EDEEDIEVPKALGDMFESVAGAIYLDSGGSLNAVWRAYYRMMKDQI----------EKCC

DCR1_WBGene00000939_Celegans ANPPRSPIRELMEFEQSKVRFSKMERILESGKVRVTVEVVNNMRFTGMGRNYRIAKATAA

EFV48890_Tspiralis ------------------------------------------------------------

EFV57735_Tspiralis CHPPISPVRHLLELEPERVQFNILDREEGNSGVHVQVVVTGKGSFIGSGKSYRTAKHAAA

EFV48479_Tspiralis CHPPISPVRHLLELEPERVQFNILDREEGNSGVHVQVVVTGKGSFIGSGKSYRTAKHAAA

EFV58865_Tspiralis LNPPISPIRDLLELEPDRVKFSRVERNAVEGKVKVAVTVEGKGRFVGAGRSYRIAKSTAA

Mhap1s0258g09126_Mhapla ENPPQSPVRELFERKNCRAKFSKLERKLETGKVRVTVTVNDNLQFTGMGRSYRIAKCTAA

Mhap1s0258g09127_Mhapla ------------------------------------------------------------

XIPH14444_m.27161_Xindex ------------------------------------------------------------

XIPH15429_m.28360_Xindex ------------------------------------------------------------

XIPH19060_m.32298_Xindex ANPPKSPIRELLELEPEKAKFAKLERDVVECKVRVTVDVYGKGRFIGVGRNYRIAKCTAA

DCR1_WBGene00000939_Celegans KRALKYLHQIEQQRRQSPSLTTV

EFV48890_Tspiralis -----------------------

EFV57735_Tspiralis KKAVRELYPTSNHFII-------

EFV48479_Tspiralis KKAVRELYPTSNHFII-------

EFV58865_Tspiralis KRALRYLKGSPQMSLISKIH---

Mhap1s0258g09126_Mhapla KRALQHLRKLDAAKNK-------

Mhap1s0258g09127_Mhapla -----------------------

XIPH14444_m.27161_Xindex -----------------------

XIPH15429_m.28360_Xindex -----------------------

XIPH19060_m.32298_Xindex KRALKYLKLLKARQTEETGERK*

DICER related complex

PASH1_WBGene00011908_Celegans MEQESGGKKSNEQLLSEREAIMRQLAMLGSGPEDLDVDDEEEDEEEEVEKMEEGVEETME

EFV57499_Tspiralis ------------------------------------------------------------

Mhap1s0197g08100_Mhapla MDSDNSD--NA---------------------DEFT-----VDELEQLEQMRNALLAELH

XIPH05737_Xindex ------------------------------------------------------------

PASH1_WBGene00011908_Celegans NASVVKETTPVKDENIVEDSNSSEELFPYLDSPPEDKIQEISANDTEIDQIDSPIRFEGV

EFV57499_Tspiralis ------------------------------------------------------------

Mhap1s0197g08100_Mhapla ------GDEDQEEDQICDEGNAEEEMNGFPNNNEENNFNSDNANI-SEDIHQNEHSFAGV

XIPH05737_Xindex ------------------------------------------------------------

PASH1_WBGene00011908_Celegans KNPAQKMDVDGEEGTSTDARPVSGSSGSSVGEQI--------RAPVPVLKTIMDRIDLDK

EFV57499_Tspiralis ------------------------------------------------------------

Mhap1s0197g08100_Mhapla IQDEDDEDSNSEEE--FDQEEISKHIDNLLEEPIKDSAKTTPKHLSKRFKRVLEYRANDH

XIPH05737_Xindex ------------------------------------------------------------

PASH1_WBGene00011908_Celegans SNPLPEGWTVISHQSGMPVYYHKFTRVVTHSKPYLVE-GIVRDHEIPVSSIPCLYKKIMD

EFV57499_Tspiralis ------------------------------------------------------------

Mhap1s0197g08100_Mhapla FNVLPDGWVEITHASGLPVYLHKPTRVCTFSRPYFIGRSSIRKHKVPESAITCLYQKKYQ

XIPH05737_Xindex ------------------------------------------------------------

PASH1_WBGene00011908_Celegans ELHENVEK-QSSKC--------------------PMTYEESQSMLEIPVKELRMSPDRYQ

EFV57499_Tspiralis -------------MKNEAAQNRQAAQTVDADLVKKLNIPETYVQTIDDYNKRNVSAEELH

Mhap1s0197g08100_Mhapla DEVEASLKAQEPSVVNEDEVDKDLREDSSNILSAKLITPDVRVKTELDQRKMQLTSDQLY

XIPH05737_Xindex ------------------------------------------------------------

PASH1_WBGene00011908_Celegans KYCEKRFKFKQITVHRYINPAEKEGVVLKKRMN--TIL---KKRGFDADYDQLKKNN---

EFV57499_Tspiralis EYAKKIFDFETIEVKRFKSWADHRAYHKKLKREKNELLMSAVITGYEDDFSDVVEHQQQQ

Mhap1s0197g08100_Mhapla NYAKNRFKFKEICIYRFGKWTEARNFYKKRKMR---------------------------

XIPH05737_Xindex ------------------------------------------------------------

PASH1_WBGene00011908_Celegans -------------KPGDVLLSSS---------------------TGAILIDLTPCPTNIN

EFV57499_Tspiralis QHQQQQHQQQVKEKNSEVEWAASTTGCGQQESSSNFNLSLSSRPKLPPNVKLITVPNYNN

Mhap1s0197g08100_Mhapla ----------------QLLVGGEARDCGQIKTGF---KTREERPGLPSDVKLITVPSLEI

XIPH05737_Xindex ------------------PVTGTQPAC-----PMANGCDATGRPSLPANVKLITVPDMEK

. :.* *

PASH1_WBGene00011908_Celegans KRSGSKKPYLLNPMGKTTVAVLNEFVQRLAKGTLLYEIEDTRNIHCPYKATALLTMKMCT

EFV57499_Tspiralis N-RSLKKQFVLNPQGKTSIAVLHEYVQKVLRGLVKYEFSETRNATNPFAAVVKLITPNTN

Mhap1s0197g08100_Mhapla DSKPNKRLFYLNPQGKTSVSILHEFVQKALKCTVRYFFSETRSSATPYHCAVKLVLNNTQ

XIPH05737_Xindex GSKPQQKPFFMNPQGRTSIAVLHEYVQKVMKGNVSYEFTETRNAANPYCAVVRIATHDRQ

:: : :** *:*::::*:*:**: : : * : :**. *: ... :

PASH1_WBGene00011908_Celegans LREM-AGQCK----------------ESL--------------VVLSEIAANDENSTTYS

EFV57499_Tspiralis TVKQTGPSNGLGAQ----------------------------GAVA-----GDGAAEATA

Mhap1s0197g08100_Mhapla TTPQSTGRKRFHNRQQNQQPTAEIVKKKLALMHEDFNKINSEQQLTSNTDAENKSDDKAV

XIPH05737_Xindex RASAVSSQNEGGAN----------------------------ES----------------

PASH1_WBGene00011908_Celegans QGLLPDLRRFPVGSGVGANKKTARLVAARDALLKLIPKLRVSEDNVCDGMVE--------

EFV57499_Tspiralis AAPSEMTDWVTIGVGNGNSKKNAKLEAARQALKVLIPGVDFNLDGQPDST----------

Mhap1s0197g08100_Mhapla DDLSPDSEFVVLGEGFGPGKKQAKMIAAKAAVEKLVPGVEFDADGIACNSNSKNELAENT

XIPH05737_Xindex GKGTGEMVWAEYGKGEGNSKKNAKLNAAKQALRVLVPGIEFNADGVADLP----------

* * * .** *:: **: *: *:* : .. *.

PASH1_WBGene00011908_Celegans -------------------------EDGTQQGFEELFKKVKIDSPNLVQMCTQCAIPKPY

EFV57499_Tspiralis ---------------------------GEQDEATKLFDLIAINDSRVPELCSKAGQPMPF

Mhap1s0197g08100_Mhapla PTSSNNPNLITSCPLMASGTSVGSSITGGDRNDLHIFDMIGVTDTRIPELCARAGQPSPY

XIPH05737_Xindex ----------------------------PQDDAGKLFDLIKISDSRVPELSAKAGQPTPF

: .:*. : : . .: ::.::.. * *:

PASH1_WBGene00011908_Celegans NLLRDAVSRSLRWNGMELVMKKEMIGNGSQLSKVILILGDI-QEEAEAVGVKQATQMASQ

EFV57499_Tspiralis IILQECIKRNASLSASKINMNIERVKHQ--LHAFTLSVGTQ-EVRVLCMNKKEGKQKASQ

Mhap1s0197g08100_Mhapla LVLQEYLKRHSAFGDTAINLTSRLLRHQ--RHEFKLSIGEELSVKVISGNKREGKQIAAQ

XIPH05737_Xindex VILQECLKRHSSFGDTTIDVGIKRLKHQ--QHEFTMKVAKH-EVKVQCTNKKEGKQRAAQ

:*:: :.* . : : . : : . : :. . .. . . ::..* *:*

PASH1_WBGene00011908_Celegans RLFKKMHPELLTYGSFLEIYGRLDDKSKIDNAKKQHDEVVRLPDTGN-------------

EFV57499_Tspiralis AMLERLHPHIKSWGALIRLYGYGAQ-RQMNASRKEKANIVKMQAESKQQQQQQHRSGATG

Mhap1s0197g08100_Mhapla AMLKKLHPEVETWGSILKLYGYEAQ-QKFRDARKNKDSVVKLQGIQDEANVRQFQ-----

XIPH05737_Xindex AMLEKLHPQITTWGSLIRLYGYGAQ-RKMQETRKEKDSIIRLQSQNKQHQTGDEPN----

:::::**.: ::*:::.:** : :: ::*:: .:::: .

PASH1_WBGene00011908_Celegans ---LLAPNFIVLSKLSEEMKNISLVYPPRKFLYGLATNSTGIKHDIRNVL----TQTLMA

EFV57499_Tspiralis DDWSSADKAKLWQSIEKGMMKVWFKEQ---------AN--GHLKPGHFF---WD---Y-Q

Mhap1s0197g08100_Mhapla ------PNSLILEKLRNEMLKLSGDLI---------KNCSRQQNDGEEPLAKFRRSDFCS

XIPH05737_Xindex ----IA----ILEKLKQEMQKLYEQRT---------MVDNSQCSSGIAA-----------

: ..: : * ::

PASH1_WBGene00011908_Celegans TLPPPPPQFFPMIGGPPLMHPTFSTSAPPPPPPPQPMEYGYNPMKQMPSRKRGRHDDSSS

EFV57499_Tspiralis HLPSASTSTAAT------ADARSAAS----------------------------------

Mhap1s0197g08100_Mhapla ELPVLSEEKLSE------LSAKFMSERPELQKCLPSVKF---------------------

XIPH05737_Xindex -------SAVKN------IDI*--------------------------------------

.

PASH1_WBGene00011908_Celegans PSHQKPHK

EFV57499_Tspiralis --------

Mhap1s0197g08100_Mhapla --------

XIPH05737_Xindex --------

Ribonuclease

Xrn-2_WBGene00006964_Celegans MGVPAFFRWLTKKYPATVVNANEDRQRDQDGNRVPVDCTQPNPNFQEFDNLYLDMNGIIH

EFV54322_Tspiralis MGVPAFFRWLSRKYPSIVMNCIEDTPRDVDGTTVPVDNTQPNPHGIEFDTFYLDMNGIIH

EFV6015_Tspiralis MGVPRFFRWLSERYPGLSQLVVES-------------------QIPSYDNLYLDFNGIIH

Mh1s0122g06368_Mhapla MGVPAFFRWLSRKYVSIIVDAVEERRKEVEGIKIPVDCTQPNLNYQEFDILYLDMNGIIH

XIPH01179_m.4106_Xindex ----------------------------------------------------------IY

XIPH04762_m.12274_Xindex ------------------------------------------------------------

XIPH11887_m.23861_Xindex ------------------------------------------------------------

Xrn-2_WBGene00006964_Celegans PCTHPEDRPA--PKNEDEMFALIFEYIDRIYSIVRPRRLLYMAIDGVAPRAKMNQQRSRR

EFV54322_Tspiralis PCCHPEDKPA--PKSEEEMMVAIFEYIDRLMCIVRPRRLLYMAIDGVAPRAKMNQQRTRR

EFV6015_Tspiralis NCSHPNSPDATFRC------------TEGLFQLIKPRKVLFIAVDGVAPRAKMNQQRSRR

Mh1s0122g06368_Mhapla PCTHPEDRPA--PKTEEEMFILIFEYVDRLFSIVRPRRLLYMAIDGVAPRAKMNQQRSRR

XIPH01179_m.4106_Xindex ACSHPNDEDPHFRITEEQIFRDIFRYIEVLYRIIRPRKVFFMAVDGVAPRAKMNQQRGRR

XIPH04762_m.12274_Xindex ------------------------------------------------------------

XIPH11887_m.23861_Xindex ------------------------------------------------------------

Xrn-2_WBGene00006964_Celegans FRASKEMAEKEASIEEQRNRLMAEGIAVPPKKKEEAHFDSNCITPGTPFMARLADALRYY

EFV54322_Tspiralis FRASKEAAEKEEQIRQIREDLRAQGIPLPAESTDKQHFDSNCITPGTPFMARLAICLRYY

EFV6015_Tspiralis FMSAKEAEDSRLKAI-------RNGEVI----PDSDPFDSNCITPGTEFMERLHIHLKYF

Mh1s0122g06368_Mhapla FRAAKEAAEKREQIASIRKRLENEGVPLPPPRKEEEHFDSNCITPGTPFMARLSTALRYY

XIPH01179_m.4106_Xindex FMSARTAQELIERAK-------SKGEKL----PTEERFDSNCITPGTHFMAKLHEQLKYF

XIPH04762_m.12274_Xindex ------------------------------------------------------------

XIPH11887_m.23861_Xindex ------------------------------------------------------------

Xrn-2_WBGene00006964_Celegans IHDRVTNDASWANIEIILSDANVPGEGEHKIMDYVRKQRGNPAHDPNTVHCLCGADADLI

EFV54322_Tspiralis IHERLNTDPAWQNLLVILSDASVPGEGEHKIMDYIRHQRACASHDPNTHHVLCGADADLI

EFV6015_Tspiralis INLKLSSDPLWQNVDVYYSGHDCPGEGEHKILAFIRFMRSQADYDSNTTHCIYGLDADLI

Mh1s0122g06368_Mhapla IHKRITYDPAWMKIQVILSDANAPGEGEHKIMDYIRRQRASPSHDPNTVHCLCGADADLI

XIPH01179_m.4106_Xindex VNMKITTDDMWQGIRIYLSGHDVPGEGEHKIMDFIRHERSQPDYDPNTRHCLYGLDADLI

XIPH04762_m.12274_Xindex ------------------------------------------------------------

XIPH11887_m.23861_Xindex ------------------------------------------------------------

Xrn-2_WBGene00006964_Celegans MLGIATHEANFNIIREEFVPNQPRACDLCGQYGHELKECRGAENETDLGDDYCKPEQREK

EFV54322_Tspiralis MLGLATHEPNFTIIREEFVPNLPRPCEICNNYGHTMQDCQGLSILENENEEAHRPVLKKT

EFV6015_Tspiralis FLGMAMHEPYFSILREEVVYDSRSKKRYI-----------------------KCDDDDDN

Mh1s0122g06368_Mhapla MLGLATHEANFNIIREEFVPNQPKACELCGQYGHELEHCQGLARVE-AGPDQVDPLKKEK

XIPH01179_m.4106_Xindex MLGVCSHEPYFCLLREEVKFVRPSKKLNV-----------------------RTANAEQT

XIPH04762_m.12274_Xindex ------------------------------------------------------------

XIPH11887_m.23861_Xindex ------------------------------------------------------------

Xrn-2_WBGene00006964_Celegans NFIFLRIPVLREYLEKELSMP---NLPFKFDVERALDDWVFLCFFVGNDFLPHLPSLEIR

EFV54322_Tspiralis QFIFIRLSVLREYLQRELEMP---NIKFKYDFERCVDDWVFMCFFVGNDFLPHLPSLEIR

EFV6015_Tspiralis N-NLLRF----AYGQFAYGRRSEQVKPDVYDEEKIIDDWIFMSFFIGNDFIPNVPMLITN

Mh1s0122g06368_Mhapla NFIFIRLPVLREYLERELFMP---NLPFAYDLERAIDDWVMMCFFVGNDFLPHLPSLEIR

XIPH01179_m.4106_Xindex TFHLLHLSLLRDYIELEFSTVKHQL-QFDFDLESIIDDWVLMGFLVGNDFIPHLPNLHIH

XIPH04762_m.12274_Xindex -----------------------------------------MCFFVGNDFLPHLPSLEIR

XIPH11887_m.23861_Xindex ------------------------------------------------------------

Xrn-2_WBGene00006964_Celegans EGAIDRLIKLYKEMVYQMKGYLTKDGIPELDRVEMIMKGLGRVEDEIFKRRQQDEERFQE

EFV54322_Tspiralis EGAIDRLVKLYKDCVYRTGGYLTENGFVNLKRVQLIMSELGKVEDEIFRQRQEREAMDNI

EFV6015_Tspiralis ENALPNVWLAYQNAFPNMDAC----GWLESKRAQRVRF------------------HFLS

Mh1s0122g06368_Mhapla ENAIDRLVKLYKNMVYQTGGWLTCDGVVNIDRVKMIMHELGKVEDQIFRERQLRELKFKE

XIPH01179_m.4106_Xindex SNALPTLYKAYMKTLPELGGYINEGGFLNLPRFEKFMAILAQFDRDSFRDEYEDL-MWLE

XIPH04762_m.12274_Xindex EGAIDRLIKLYKDCVYKTGGYLTDSGFVELSRVQLILSELGAVEDEIFRSRQQNELEFRR

XIPH11887_m.23861_Xindex ------------------------------------------------------------

Xrn-2_WBGene00006964_Celegans N-----QRNKKARMQMYGGGGRGGRGRGRGRGQQPAFVPTHGILA----PMAAPMHHSGE

EFV54322_Tspiralis FLLDWIQFSNKAKMRR----------MQAENFDAPAFIPQNAFAP----TP------IGE

EFV6015_Tspiralis SSPM-------------LFIAFSF-RM------HDSRAVEKIFLRLQ---QITSFSSQSE

Mh1s0122g06368_Mhapla SQKQRKRRAKEA---------------------AEMLMPYNASLIAPQPISSQNLNKSGE

XIPH01179_m.4106_Xindex SKSS-------------GGPKFGS-RD------VRESVPRKIVLVEEEPKPLVPYVDSEN

XIPH04762_m.12274_Xindex RQKERRQRMKAATA--------------------PSYIPGNKFGAFAPNQHQGMARMSGG

XIPH11887_m.23861_Xindex ------------------------------------------------------------

Xrn-2_WBGene00006964_Celegans STRQMASEARQTAMKFT--------------------------NDANETA--A--ANLK-

EFV54322_Tspiralis SPLPL-SNAKRTAMEMR--------------------------QAA--------------

EFV6015_Tspiralis TA--NGS---SDSCVL---QSLVV-------FSEKLPDTCSCALE---------------

Mh1s0122g06368_Mhapla EIRKMAADDRKQ-MQLAAEQAQKLKSLLTPVAVTTIGSKRKMEQTLNDSPSGS--PPIK-

XIPH01179_m.4106_Xindex EGLGTGSNSNSDKSSSP--RAEAL---AESTLEGTSGEDLDCADYEEDEDISHLPPELQD

XIPH04762_m.12274_Xindex EARQMAYDMRVA-------------------------------NKANEDAARSLKAQLG-

XIPH11887_m.23861_Xindex ------------------------------------------------------------

Xrn-2_WBGene00006964_Celegans -----A------LLNVKGEES------PADIASRKRKAEQPL-IKPEEEEDEGPKDDIRL

EFV54322_Tspiralis ----------------------------MAVTSSTKREFNGA-ANAEDANDEGPLDEVRL

EFV6015_Tspiralis ---NDDGTLFDDDDD-DDEDSEDA----VDMRSETCDGLYEVSVL--TEE----------

Mh1s0122g06368_Mhapla -----DGRVVEDKSTLDGPKPEIVFTTPSGGKAPLAKGAVGA-LMNDDSDEEETTDEIRL

XIPH01179_m.4106_Xindex LIRRSDANIKNSESSADDDMTEE--------DAKMSKGLVNYELCGYDEDDDSWSPDMEK

XIPH04762_m.12274_Xindex -----GGP-VA--------------DTPRPDGQGGAKRTHDQ-VKADSDEEQEPVDEVRL

XIPH11887_m.23861_Xindex ------------------------------------------------------------

Xrn-2_WBGene00006964_Celegans YESGWKDRYYRAKFDVGSDDIEFRHRVAWAYVEGLCWVLRYYYQGCASWDWYFPYHYAPF

EFV54322_Tspiralis WECGWKDRYYLVKFQCSPKDLEFRHHVANCYVEGLCWVLRYYYQGCCSWKWYFPFHYSPF

EFV6015_Tspiralis ---------------------------------ESDMSLHSLFRK--FRASFYGFHYAPF

Mh1s0122g06368_Mhapla YEEGWKERYYRAKLHVEESDTELRKNVVCAYIEGLSWVLLYYYQGCASWTWYFPYHYAPF

XIPH01179_m.4106_Xindex AFRRFKSNYYMDKMSYNDVTAEVLRDQAVQYVRAIQWNLHYYYNGVCSWSWYYPHHYAPY

XIPH04762_m.12274_Xindex WEAGWKERYYQAKFEISSEDRNFRYKVAQAYVEGLCWVLRYYYQGVPDWSWFYPYYYAPF

XIPH11887_m.23861_Xindex ------------------------------------------------------------

Xrn-2_WBGene00006964_Celegans ASDFETVGEFQPDFTRPTKPFNPLEQLMSVFPAASKQHLPVEWQKLMI---QDDSPIIDL

EFV54322_Tspiralis ASDFLNIGDLKIDFSEKTMPIKPLEQLMSVFPAASSKHLPKSWAALMHDPVRDKSTIIDM

EFV6015_Tspiralis VSDLCDFNVGEITFEL-GEPFLPFQQLLAVLPPSSRKLLPEPYQDLMV---NPDSPLSYA

Mh1s0122g06368_Mhapla ASDFYLAGDYKPDFSKSTRPFKPLEQLMGVFPAASRAHIPKGWHWLMT---DEKSPIIDF

XIPH01179_m.4106_Xindex LSDVKNFSDLEIHFEL-GEPFVPFQQLLAVLPAASRKLLPSGYQALMT---NADSPLIDF

XIPH04762_m.12274_Xindex ASDFMDIGQLKPHFNLDSEPVTPLVQLMSVFPAASRTHLPEAWSNLMV---TKESPIIDF

XIPH11887_m.23861_Xindex ------------------------------------------------------------

Xrn-2_WBGene00006964_Celegans YPADFRIDLNGKKYAWQGVALLPFVDETRLLATLQSVYPTLTAEEKQRNTRGPNRIFIGR

EFV54322_Tspiralis YPSDFKVDLNGKRYAWQGVVLLPFVDAERLNEALEVVYPDLTEEERFRNKQGNDLLFISS

EFV6015_Tspiralis YPEDVERDMNGKKYEWECILKLPFIDEEILFDAVYPFNDALKDDEKRRNRFGKCYLYRYS

Mh1s0122g06368_Mhapla YPDDFEIDLNGKKYAWQGVALLPFVDEKRLLDALNLVYDILGDEERARNDTGPDRLFAGK

XIPH01179_m.4106_Xindex YPSHFETDLNGKKNDWEAVILIPFIKEDRLLEAMRPLDAMLAKEEKARNKPGKHLLYTTA

XIPH04762_m.12274_Xindex YPSDFKVDLNGKKFDYW*------------------------------------------

XIPH11887_m.23861_Xindex ------------------------------------------------------------

Xrn-2_WBGene00006964_Celegans NHKSF---EFF-QQVAESK-----------------------------------------

EFV54322_Tspiralis KHEAF---DFI-QSIYEGD-----------------------------------------

EFV6015_Tspiralis GSHGERRTTTFRSSLPDIFPDIENCTCEYSYIQRNFNN----------------------

Mh1s0122g06368_Mhapla QHGLF---ELM-HSVHQLTKENNGNSIVTSSVER-------SRISTGNSQSVESERSQSN

XIPH01179_m.4106_Xindex I----QDQGYYRSSLVGVFPDLDHNKAMCKELDRNAFRLQPSQIRKGRLPGIRSE-----

XIPH04762_m.12274_Xindex ------------------------------------------------------------

XIPH11887_m.23861_Xindex ------------------------------------------------------------

Xrn-2_WBGene00006964_Celegans ------------------------------------------------------------

EFV54322_Tspiralis ------------------------------------------------------------

EFV6015_Tspiralis ------------------------------------------------------------

Mh1s0122g06368_Mhapla EEDSNERSQSTEESNDRSQSNEGDSNERSQSNEEETNEEQSHSTGEEQSRITSNETNSGE

XIPH01179_m.4106_Xindex ------------------------------------------------------------

XIPH04762_m.12274_Xindex ------------------------------------------------------------

XIPH11887_m.23861_Xindex ------------------------------------------------------------

Xrn-2_WBGene00006964_Celegans ---------SDDLVPLDPTLLNGVSGKIAYDSTATAPGLPFVSPVN-HDECQDLPTNCGI

EFV54322_Tspiralis --------MSAEWLNMDPSLCNGISLMVKPYKFHVPVGKTVHSP---LPQCNDVENNHVL

EFV6015_Tspiralis ------------------------------------------------------------

Mh1s0122g06368_Mhapla NNEIVVAKDEKQWVIIDASKAYGMAGEIHPDESAIDYGVDYVSMFRGSIEFGDIKKNSCV

XIPH01179_m.4106_Xindex ------------------SYVLGFSTLK-----HLPYT--GEVKFMGVQVFQQVSKNESV

XIPH04762_m.12274_Xindex ------------------------------------------------------------

XIPH11887_m.23861_Xindex -------------------------------------------MRSVLPGFPDVMNNHVA

Xrn-2_WBGene00006964_Celegans C-VLYEDPEYPQDYIFPALRLDGA----KEPEKTLKPDDWNDRRDGRYQPQVGFNRNAPR

EFV54322_Tspiralis S-VFCLNPQFPDDYIFSTARLSGACCLFRDPQPVLKPKDWDDDRDGRYRPVTGFVQSAVT

EFV6015_Tspiralis ------------------------------------------------------------

Mh1s0122g06368_Mhapla M-VQFRDPQFPPRFQFKACRLDGI----KELPRALKPQNYDDRRQGPYRPQIGFSRDIPR

XIPH01179_m.4106_Xindex LVKLLEEPRCSQTLAETADRLVGKEVFVNYPHLIV-----------------GLVTGVSN

XIPH04762_m.12274_Xindex ------------------------------------------------------------

XIPH11887_m.23861_Xindex -VVLYQHPIFPRGFRFSAARLKGA----VDPPSTLKPQDWNDR-NEKYRPMIGFSRDVPR

Xrn-2_WBGene00006964_Celegans GSLDQSGHRQVHHYVRGGGGGGGGYRGNSYDDRRGG--------------GGGGGGY---

EFV54322_Tspiralis AQLNRASKRILE------------------------------------------------

EFV6015_Tspiralis ------------------------------------------------------------

Mh1s0122g06368_Mhapla ASLSSSGHRTLDHYVTQQNRRSGPASLFAYQAQQPQQVQASPQQQLPWHSGAAQQQY---

XIPH01179_m.4106_Xindex ---------ESERYMKDNSKDVNHVSLN-------------EKDAKAWNDVAEESKT---

XIPH04762_m.12274_Xindex ------------------------------------------------------------

XIPH11887_m.23861_Xindex AELDIAGKRMLRAGIGAGPPGSNRYEPYS-----RPQYETQNYQDRSYHNANDRHYRNSS

Xrn-2_WBGene00006964_Celegans ---NDR--QDFG----RNYGG----RDGGGPQRYHDQQQ-QRQGGY-----------QGG

EFV54322_Tspiralis ------------------------------------------------------------

EFV6015_Tspiralis ------------------------------------------------------------

Mh1s0122g06368_Mhapla ---QQLMPSDFQQPLPLNFMQMAMPPPPLPPQHWVNPMQQQQQQPYVGQWGQPPSLLQNQ

XIPH01179_m.4106_Xindex ---T*-------------------------------------------------------

XIPH04762_m.12274_Xindex ------------------------------------------------------------

XIPH11887_m.23861_Xindex DPHRSQMGSQYRQGAPYNPNYHRSYESQGGQPHWNQQ-----------------------

Xrn-2_WBGene00006964_Celegans GYGGGYG-----GG--GGGGGGGGGGSYHQPYNQDQRRGGRGGGGGPPGYQRPPYRGGG-

EFV54322_Tspiralis ------------------------------------------------------------

EFV6015_Tspiralis ------------------------------------------------------------

Mh1s0122g06368_Mhapla NYGGNNNSDTWRRRSDDGGGGSSRSNQQHLPYHNNQYGRS-----SVRNYNNDDNRNNSN

XIPH01179_m.4106_Xindex ------------------------------------------------------------

XIPH04762_m.12274_Xindex ------------------------------------------------------------

XIPH11887_m.23861_Xindex ----GRSGADWRGQNPHGPP------PPRYPPHQDP------------------YTN---

Xrn-2_WBGene00006964_Celegans --GGGYH-GNSSWR-

EFV54322_Tspiralis ---------------

EFV6015_Tspiralis ---------------

Mh1s0122g06368_Mhapla SSQREYR-SNQRRQ-

XIPH01179_m.4106_Xindex ---------------

XIPH04762_m.12274_Xindex ---------------

XIPH11887_m.23861_Xindex --QGHYERRQKTWR*
