# Supplementary material for: In Vitro Acquisition of Specific Small Interfering RNAs Inhibits the Expression of Some Target Genes in the Plant Ectoparasite Xiphinema index
Source: Int J Mol Sci. 2019 Jul 3;20(13):3266. doi: 10.3390/ijms20133266 (PMC6651894; doi:10.3390/ijms20133266)
Supplement: Supplementary file 1 [file ijms-20-03266-s001.zip › Table S1 Marmonier IJMS revised MS.docx]

**Table S1:** Selection of candidate genes for RNAi experiments in *X. index*

| **Candidate genes**^1^ | **Orthologs in *X. index***  **Accession number** |
| --- | --- |
| ***laminin*** | CV581502 |
| ***piccolo protein*** | CV127411 |
| ***cysteine rich venom protein*** | CV127457 |

^1^gene annotated in root-knot nematode genomes and potentially encoding similar proteins in *X. index*
