# Supplementary material for: In Vitro Acquisition of Specific Small Interfering RNAs Inhibits the Expression of Some Target Genes in the Plant Ectoparasite Xiphinema index
Source: Int J Mol Sci. 2019 Jul 3;20(13):3266. doi: 10.3390/ijms20133266 (PMC6651894; doi:10.3390/ijms20133266)
Supplement: Supplementary file 1 [file ijms-20-03266-s001.zip › Table S2 Marmonier IJMS revised MS.docx]

**Table S2:** Primer sequences used for the RT-qPCR analysis

| **Gene** | **Sequence (5’---3’)** | **Sense** |
| --- | --- | --- |
| ***elongation factor*** | TCA-CTG-GTA-CGT-CTC-AAG-CCG-ACT-G | forward |
|  | AGT-GTA-GGC-GAG-CAG-ACC-GTG-TTC | reverse |
| ***18S*** | ACC-ACA-CTT-CTT-AGA-GGG-ACA-ACC | forward |
|  | AGG-CGA-CGG-CAC-ATA-CTG-ATT-C | reverse |
| ***tubulin*** | GGC-ACC-TAC-CGT-CAA-CTA-TTC-CAT-C | forward |
|  | ACG-AAC-ACG-ATC-CAG-CAC-CAA-ATC | reverse |
| ***NADH dehydrogenase*** | ATT-TTA-GCC-TTA-ATA-ATC-GGT-CTA-ACC-C | forward |
|  | TTA-TAC-ATA-GTG-AGG-TGC-TAG-TAA-AGC-C | reverse |
| ***laminin*** | CTC-CGA-ATA-GTG-CTG-TAG-AAG-A | forward |
|  | TGA-TCC-ATT-ACG-CCG-TAT-GAT | reverse |
| ***piccolo protein*** | CGA-TCG-GCC-GGT-TTC-TGT-GT | forward |
|  | CAC-TGG-ATG-GCC-GTC-TTG-CT | reverse |
| ***cysteine rich venom protein*** | TGT-GAT-CTG-TGA-GGC-TAC-CAA | forward |
|  | TGA-CAA-CCA-GCA-ACG-CAT-T | reverse |
