# Supplementary material for: In Vitro Acquisition of Specific Small Interfering RNAs Inhibits the Expression of Some Target Genes in the Plant Ectoparasite Xiphinema index
Source: Int J Mol Sci. 2019 Jul 3;20(13):3266. doi: 10.3390/ijms20133266 (PMC6651894; doi:10.3390/ijms20133266)
Supplement: Supplementary file 1 [file ijms-20-03266-s001.zip › Table S4 Marmonier IJMS revised MS.docx]

**Table S4:** Primer sequence used for siRNA synthesis

| **Candidate gene** | **siRNA** | **siRNA sequence** | **Start position on the target gene** | **Size (bp)** | **% GC** |
| --- | --- | --- | --- | --- | --- |
| ***Laminin*** | L1 | sense : 5’-CUACGAACGAGUCAAUUUAdTdT- 3’  antisense : 3’-dTdTGAUGCUUGCUCAGUUAAAU-5’ | 269 | 21 | 33 |
|  | L2 | sense : 5’-UGGAUCAUUCUUUCAAUCGdTdT-3’  antisense : 3’-dTdTACCUAGUAAGAAAGUUAGC-5’ | 497 | 21 | 33 |
|  | L3 | sense : 5’-UUCGACACUUCCGAUUUAUdTdT-3’  antisense : 3’-dTdTAAGCUGUGAAGGCUAAAUA-5’ | 535 | 21 | 33 |
| ***Piccolo protein*** | P1 | sense : 5’-GGAAUUUCGAAUAUGAAAAdTdT-3’  antisense : 3’-dTdTCCUUAAAGCUUAUACUUUU-5’ | 156 | 21 | 24 |
|  | P2 | sense : 5’-AUCGUGACGUGGCAAAAAGdTdT-3’  antisense : 3’-dTdTUAGCACUGCACCGUUUUUC-5’ | 94 | 21 | 43 |
|  | P3 | sense : 5’-ACCAUUUACGUAUUUUUGGdTdT-3’  antisense : 3’-dTdTUGGUAAAUGCAUAAAAACC-5’ | 192 | 21 | 29 |
| ***Cysteine rich venom protein*** | V1 | sense : 5’-UAUUUACGUUACGAUAAAAdTdT-3’  antisense : 3’-dTdTAUAAAUGCAAUGCUAUUUU-5’ | 53 | 21 | 19 |
|  | V2 | sense : 5’-GUUAGAAAAUAGAUAAUGUdTdT-3’  antisense : 3’-dTdTCAAUCUUUUAUCUAUUACA-5’ | 342 | 21 | 19 |
|  | V3 | sense : 5’-CGUCGCCUAUUCCAUUUCUdTdT-3’  antisense : 3’-dTdTGCAGCGGAUAAGGUAAAGA-5’ | 26 | 21 | 42 |
| **Negative control** | C- | sense : 5’-UAGAGCUAACACUGAACUUdTdT-3’  antisense : 3’-dTdTAUCUCGAUUGUGACUUGAA-5’ | / | 21 | 33 |
