# Supplementary material for: In Vitro Acquisition of Specific Small Interfering RNAs Inhibits the Expression of Some Target Genes in the Plant Ectoparasite Xiphinema index
Source: Int J Mol Sci. 2019 Jul 3;20(13):3266. doi: 10.3390/ijms20133266 (PMC6651894; doi:10.3390/ijms20133266)
Supplement: Supplementary file 1 [file ijms-20-03266-s001.zip › Table S6 Marmonier IJMS revised MS.docx]

**Table S6**: List of *X. index* putative proteins involved in gene silencing

| ***C. elegans ortholog***^1^ | ***X. index* protein accession number** | **Presence of PF02170 (PAZ) domain** | **Presence of PF02171 (PIWI) domain** | ***X. index* putative protein size**^2^ |
| --- | --- | --- | --- | --- |
| ALG  RDE-1  ZK218,8 | XIPH00171_m.824 | no | YES | 424 |
|  | XIPH00220_m.1024 | YES | YES | 808 |
|  | XIPH00682_m.2641 | YES | YES | 807 |
|  | XIPH01058_m.3772 | no | YES | 424 |
|  | XIPH01240_m.4301 | no | YES | 302 |
|  | XIPH01422_m.4808 | YES | YES | 616 |
|  | XIPH01763_m.5725 | YES | YES | 570 |
|  | XIPH03015_m.8656 | no | YES | 488 |
|  | XIPH03061_m.8764 | YES | YES | 601 |
|  | XIPH03137_m.8937 | no | YES | 167 |
|  | XIPH05696_m.14049 | no | YES | 130 |
|  | XIPH11139_m.22857 | no | YES | 228 |
|  | XIPH11895_m.23873 | no | YES | 130 |
|  | XIPH13915_m.26514 | YES | no | 282 |
|  | XIPH14718_m.27510 | no | YES | 167 |
|  | XIPH17950_m.31169 | no | YES | 113 |
|  | XIPH28640_m.40226 | YES | no | 145 |
| ALG-3/ALG4 | XIPH00282_m.1279 | YES | YES | 927 |
|  | XIPH00358_m.1579 | YES | YES | 995 |
| PRG-1 | XIPH00931_m.3408 | no | YES | 464 |
| ERI-1 | XIPH19513_m.32755 | no | no | 169 |
| DCR-1 | XIPH14444_m.27161 | no | no | 275 |
|  | XIPH15429_m.28360 | no | no | 231 |
|  | XIPH19060_m.32298 | no | no | 154 |
| PASH-1 | XIPH05737_m.14136 | no | no | 347 |
| Xrn-2 | XIPH01179_m.4106 | no | no | 814 |
|  | XIPH04762_m.12274 | no | no | 320 |
|  | XIPH11887_m.23861 | no | no | 204 |
| **12** ***X. index* protein carrying Argonaute domain(s) without grouping with *C. elegans* proteins** | | | | |
|  | XIPH01514_m.5059 | YES | YES | 593 |
|  | XIPH06907_m.16176 | no | YES | 428 |
|  | XIPH02759_m.8084 | YES | YES | 626 |
|  | XIPH03384_m.9480 | YES | no | 579 |
|  | XIPH08308_m.18504 | YES | no | 363 |
|  | XIPH20626_m.33839 | no | YES | 132 |
|  | XIPH06697_m.15830 | no | YES | 226 |
|  | XIPH03189_m.9049 | no | YES | 254 |
|  | XIPH12779_m.25056 | no | YES | 138 |
|  | XIPH13497_m.25977 | no | YES | 174 |
|  | XIPH02154_m.6680 | no | YES | 307 |
|  | XIPH05239_m.13176 | no | YES | 355 |

^1^names of genes are indicated for the nematode model species C. elegans.

^2^size of the putative protein in amino acids are indicated for *X. index.*
