# Supplementary material for: In Vitro Acquisition of Specific Small Interfering RNAs Inhibits the Expression of Some Target Genes in the Plant Ectoparasite Xiphinema index
Source: Int J Mol Sci. 2019 Jul 3;20(13):3266. doi: 10.3390/ijms20133266 (PMC6651894; doi:10.3390/ijms20133266)
Supplement: Supplementary file 1 [file ijms-20-03266-s001.zip › Table S7 Marmonier IJMS revised MS.docx]

**Table S7**: List of the 12 species used for functional comparative annotation of *X. index* putative proteins involved in gene silencing

| Nematode species | Parasite | | Protein databases and references |
| --- | --- | --- | --- |
|  | Plant | Animal |  |
| *Xiphinema index* | ✓ |  | [[1](#_ENREF_1)]  Sequence Read Archive (SRA) accession numbers PRJEB22758 |
| *Longidorus elongatus* | ✓ |  |  |
| *Caenorhabditis elegans* |  |  | WORMBASE  caenorhabditis_elegans-romain-2015.pep.fa |
| *Trichinella spiralis* |  | ✓ | WORMBASE PARASITE  trichinella_spiralis.PRJNA12603.WBPS8.protein.20170323.fa |
| *Globodera pallida* | ✓ |  | WORMBASE PARASITE  globodera_pallida.v1.0.AA.20170327.fa |
| *Globodera rostochiensis* | ✓ |  | WORMBASE PARASITE  globodera_rostochiensis.nGr.v1.1.augustus.manual.20150323.fa |
| *Bursaphelenchus xylophilus* | ✓ |  | WORMBASE PRJEA64437 version:WBPS8 bursaphelenchus_xylophilus.PRJEA64437.WBPS8.protein.20170327.fa |
| *5 Meloidogyne spp.* | | | |
| *M. incognita* | ✓ |  | [[2](#_ENREF_2), [3](#_ENREF_3)]  meloidogyne.inra.fr |
| *M. javanica* | ✓ |  |  |
| *M. arenaria* | ✓ |  |  |
| *M. hapla* | ✓ |  | WORMBASE PARASITE  meloidogyne_hapla.v1.2015.fpa |
| *M. floridensis* | ✓ |  | WORMBASE PARASITE  meloidogyne_floridensis.v1.2015.fpa |

1. Danchin, E. G. J.; Perfus-Barbeoch, L.; Rancurel, C.; Thorpe, P.; Da Rocha, M.; Bajew, S.; Neilson, R.; Guzeeva, E. S.; Da Silva, C.; Guy, J.; Labadie, K.; Esmenjaud, D.; Helder, J.; Jones, J. T.; den Akker, S. E., The Transcriptomes of Xiphinema index and Longidorus elongatus Suggest Independent Acquisition of Some Plant Parasitism Genes by Horizontal Gene Transfer in Early-Branching Nematodes. *Genes* **2017,** 8, (10).

2. Blanc-Mathieu, R.; Perfus-Barbeoch, L.; Aury, J. M.; Da Rocha, M.; Gouzy, J.; Sallet, E.; Martin-Jimenez, C.; Bailly-Bechet, M.; Castagnone-Sereno, P.; Flot, J. F.; Kozlowski, D. K.; Cazareth, J.; Couloux, A.; Da Silva, C.; Guy, J.; Kim-Jo, Y. J.; Rancurel, C.; Schiex, T.; Abad, P.; Wincker, P.; Danchin, E. G. J., Hybridization and polyploidy enable genomic plasticity without sex in the most devastating plant-parasitic nematodes. *PLoS genetics* **2017,** 13, (6), e1006777.

3. Pratx, L.; Rancurel, C.; Da Rocha, M.; Danchin, E. G. J.; Castagnone-Sereno, P.; Abad, P.; Perfus-Barbeoch, L., Genome-wide expert annotation of the epigenetic machinery of the plant-parasitic nematodes Meloidogyne spp., with a focus on the asexually reproducing species. *BMC genomics* **2018,** 19, (1), 321.
